# SLEEPYLAND: trust begins with fair evaluation of automatic sleep staging models

**DOI:** 10.1038/s41746-025-02237-2

**Published:** 2025-12-16

**Authors:** Alvise Dei Rossi, Matteo Metaldi, Michal Bechny, Irina Filchenko, Julia van der Meer, Markus H. Schmidt, Claudio L. A. Bassetti, Athina Tzovara, Francesca D. Faraci, Luigi Fiorillo

**Affiliations:** 1 Institute of Digital Technologies for Personalized Healthcare ∣ MeDiTech, University of Applied Sciences and Arts of Southern Switzerland, Lugano, Switzerland; 2 Faculty of Informatics, Universitá della Svizzera Italiana, Lugano, Switzerland; 3 Institute of Informatics, University of Bern, Bern, Switzerland; 4 Sleep Wake Epilepsy Center ∣ NeuroTec, Inselspital, Bern University Hospital, University of Bern, Bern, Switzerland; 5 Neurocenter of Southern Switzerland, Ente Ospedaliero Cantonale, Lugano, Switzerland

**Keywords:** Biomedical engineering, Computer science, Software, Statistics

## Abstract

Automatic sleep staging with deep learning has advanced considerably, yet clinical adoption remains hindered by limited generalization, model bias, and inconsistent evaluation practices. We present SLEEPYLAND, an open-source framework comprising ~ 220,000 h of in-domain and ~ 84,000 h of out-of-domain polysomnographic recordings, spanning diverse ages, disorders, and hardware configurations. We release pre-trained state-of-the-art models, evaluating them across single- and multi-channel EEG/EOG setups. We introduce SOMNUS, an ensemble that integrates models via soft-voting, achieving robust performance across 24 datasets (macro-F1, 68.7–87.2%), outperforming individual models in 94.9% of cases and exceeding prior state-of-the-art. Exploiting the Bern-Sleep-Wake-Registry (*N* = 6633), we show that while SOMNUS improves generalization, no model architecture consistently minimizes model demographic/clinical bias. On multi-annotated datasets, SOMNUS surpasses the best human scorer (macro-F1, 85.2% vs 80.8% on DOD-H, and 80.2% vs 75.9% on DOD-O), more closely reproducing consensus. Finally, ensemble disagreement metrics predict scorer ambiguity (ROC-AUC 82.8%), providing reliable proxies for human uncertainty.

## Introduction

Polysomnography (PSG) is the gold standard of sleep diagnostics, widely used for common disorders such as sleep breathing disorders, narcolepsy, and sleep-related movement disorders. PSG involves the recording of multiple biosignals during the whole night. These may include electroencephalographic activity (electroencephalogram—EEG), eye movements (electrooculogram—EOG), muscle activity (electromyogram EMG—derivations for the chin and legs), body position (video camera and accelerometer), heart rhythm (electrocardiogram), breathing (respiratory airflow, oxygen saturation, and respiratory effort indicators), as well as other vital parameters. Sleep staging is the procedure of extracting sleep cycle information from PSG signals. Wakefulness and sleep stages (i.e., NREM1, NREM2, NREM3 and REM) can be identified mainly analyzing the EEG, EOG and EMG signals. Sleep staging is performed worldwide by sleep experts, most commonly according to the AASM scoring manual (version 2.4)^1^.

Many machine learning (ML) and deep learning (DL) based algorithms have been proposed to tackle the sleep staging task^2–8^, achieving very good results in terms of overall accuracy. In particular, DL based staging algorithms have recently shown higher performance compared to the traditional ML approaches. However, none of these algorithms has ever been introduced into the daily clinical routine^8^. This gap is largely due to several interrelated challenges. *Which algorithm should I use, and how can I fairly compare their performance?* There is a lack of standardized protocols for training, validating, and testing these algorithms, making direct performance comparisons across studies difficult and often unreliable. *Will this algorithm work well beyond the dataset it was trained on?* Only a few of these algorithms have been evaluated on out-of-domain data, resulting in limited evidence of their ability to generalize well across different hardware configurations or sleep labs. *Can the model learn from and adapt to data from diverse sources and patient populations?* Sleep data is heterogeneous, varying across acquisition systems, clinical sites, and patient characteristics, including those with different sleep-wake disorders. Models must be trained on and evaluated against this diversity, quantifying their bias against the heterogeneity, e.g., clinical confounders^9–11^. *How can we account for differences in human scorers?* Inter- and intra-scorer variability is a major challenge, highlighting the need for systems that can be personalized or adapted to individual physicians’ scoring styles^12^.

Addressing these challenges requires—as a first concrete step—access to large, diverse datasets to train and validate algorithms robustly. Sleep data are difficult to store, manage, and, most importantly, share with the sleep community for study research purposes (up to 1GB for each PSG). In this regard, the National Sleep Research Resource (NSRR)^13,14^ has emerged as a resource of primary importance, providing a centralized repository of harmonized sleep data from multiple cohorts and clinical trials. To date, the NSRR provides the opportunity to search and download large amounts of harmonized sleep data from multiple cohorts, clinical trials, and other data sources (up to 10TB currently stored on the resource). The NSRR repository currently hosts 36 datasets, including an archive of almost 53k individuals represented, i.e., PSG recordings, actigraphy, questionnaires and demographic data. Most importantly, all datasets are harmonized, i.e., they are preprocessed and formatted using a common standard, facilitating integration and analysis across heterogeneous sources.

The NSRR ORD community has recently provided an open source software *Luna* (https://zzz.bwh.harvard.edu/luna/)^14^, a C/C++ and R based open-source library able to further manually annotate and analyze the large numbers of NSRR sleep studies. *Luna* is mainly focused on the overall automation of sleep analysis (e.g., time-frequency analysis, hypnograms, sleep patterns detection, etc.). The NSRR ORD community also released the *Moonlight* (https://remnrem.net/) tool, a useful interactive viewer for PSG data implemented on top of the *Luna* package. Combrisson et al. also developed *Sleep*^15^, an open-source python-based graphical user interface designed for the visualization, scoring, and analysis of sleep data. *Sleep* allows to dynamically display polysomnographic data, including spectrograms, hypnograms, and topographic maps. It also integrates automated detection algorithms for essential sleep features, such as spindles, K-complexes, slow waves, and REM events, alongside essential signal processing tools like re-referencing and filtering. As *Luna*, *Sleep* is compatible as well with common formats like the European Data Format (EDF).

To date, the extensive NSRR environment does not yet include advanced DL-based staging models, nor has any open-source tool been released to systematically compare and evaluate recently proposed models. There is a clear need for a user-friendly and robust platform to benchmark high-performing algorithms, particularly as researchers seek generalizable and reliable solutions. To address the challenges mentioned above, and enhance the usability of the recently implemented sleep staging models, we introduce SLEEPYLAND, an open-source repository that centralizes and benchmarks high-performing ML- and DL-based sleep staging algorithms. Built on the foundation of multiple-diverse sleep datasets, including the highest number of PSG recordings from the NSRR repository, SLEEPYLAND facilitates the rigorous evaluation of different models while ensuring generalizability across different domains. To make our tool accessible to non-technical users, we also developed a user-friendly graphical user interface (GUI), lowering the entry barrier for researchers interested in testing and exploring its capabilities without requiring advanced computational expertise. The GUI runs locally on the user’s device, ensuring that all data remains private and secure, with no data leaving the user’s environment.

SLEEPYLAND key contributions can be summarized as follows:

(i) Benchmarking, ensembling and generalization. We propose a unified protocol to train, validate and test high-performing sleep staging algorithms on the same set of data. For the first time, we publicly release all the models pre-trained on the largest collection of PSG recordings to date, enabling researchers to evaluate models on their own data and benchmark novel approaches using a common framework. We assess the generalizability of multiple algorithms and their ensemble, i.e., SOMNUS (Soft-voting Over Multiple Networks for Unified Sleep-staging), on the largest out-of-domain evaluation dataset used in literature to date. We demonstrate that SOMNUS leads to consistently superior performance across all evaluation settings. SOMNUS combines predictions from multiple architectures and channel configurations, and achieves macro-F1 scores that are better than any individual model. The ensemble mitigates weaknesses specific to network architecture and signal channel configurations, yielding consistently superior and more stable performance across diverse test conditions. SOMNUS surpasses previous state-of-the-art methods, even including cases where compared models were trained in-domain while SOMNUS treated the same data as out-of-domain.
(ii) Model-bias on performance and clinical markers. Exploiting the ensemble of the models, we quantify the bias on performance and prediction-derived clinical markers across demographics and clinical subgroups, including age, gender, apnea-hypopnea index (AHI) and periodic limb movement index (PLMI), on a huge out-of-domain dataset. SOMNUS does not reduce or compensate for demographic or clinical model bias. All models—including ensembles—show comparable bias patterns with respect to age, gender, AHI, and PLMI. No architecture seems to consistently minimizes errors in derived clinical markers.
(iii) Model-ensemble versus human-ensemble. We tested our tool on out-of-domain datasets scored by multiple human experts. We find that SOMNUS exceeds individual experts in alignment with the consensus, consistently aligning more closely with collective annotations than with any single expert. Its internal variability, quantified via entropy and model-to-model divergence, reliably identifies ambiguous regions, offering a data-driven proxy for human uncertainty in sleep staging.

## Results

### Datasets and experiments

We train and evaluate three state-of-the-art deep learning models, including U-Sleep^9^, DeepResNet^10^, and SleepTransformer^16^ using harmonized subsets of the NSRR repository—comprising up to $27{\prime} 494$ recordings (details in Supplementary Notes: Datasets). These models were chosen for their strong performance—comparable to inter-scorer agreement—and for their training on large, heterogeneous datasets. They further capture complementary architectural paradigms: U-Sleep uses a fully convolutional design, DeepResNet incorporates recurrent components, and SleepTransformer employs attention-based mechanisms, each analyzing signals with different methodological principles. The NSRR data are processed to facilitate fair benchmarking and generalization across the different models. Our experimental framework includes both single-channel (EEG or EOG) and multi-channel (e.g., EEG and EOG) configurations. We currently exclude the chin EMG channel mainly because not all recordings include at least one EMG channel (this choice allows us to retain the maximum number of recordings and ensure consistency in the amount of data across all experiments). In both single- and multi-channel configurations, we consider all possible EEG and EOG derivations available within each specific dataset (details in Supplementary Table 1). The data pre-processing and the data sampling across all the datasets is implemented as described in ^9^, with minor adjustments to align with SLEEPYLAND’s modular architecture. The signals are resampled to 128 Hz and rescaled per channel and per subject, such that for each channel the EEG signal has a median of 0 and an interquartile range (IQR) of 1. Values with an absolute deviation from the median exceeding 20 times the IQR are clipped. No additional filtering was applied to the signals during the pre-processing procedure. Signal segments outside the range of the scored hypnogram are trimmed. Recordings originally scored according to the Rechtschaffen and Kales (R&K) rules^17^ yield six scoring classes—awake, N1, N2, N3, N4, and REM. To conform to the AASM standard, we merge stages N3 and N4 into a single N3 stage. Additionally, during training, loss contributions from epochs labeled as MOVEMENT and UNKNOWN are masked to avoid confounding the learning process. To enhance the robustness and heterogeneity of the benchmark datasets, and to test the performance of the models across different clinical subpopulations, we exploit the Bern Sleep-Wake Registry (BSWR)^18^—out-of-domain real-world dataset. The dataset comprises $8410$ recordings from patients and healthy subjects aged 0−91 years, collected at the Department of Neurology, Inselspital, University Hospital Bern, in a clinical routine between 2000 and 2021. This dataset, approved for secondary use by the cantonal ethics committee (KEK-Nr. 2022-00415), uniquely spans the full spectrum of sleep-wake disorders, including cases with multiple comorbidities and non-sleep-related conditions. Unlike publicly available datasets, the BSWR provides an exceptionally diverse PSG dataset, making it a critical addition to our benchmarking efforts. Additionally, to evaluate the performance of the ensemble of the sleep staging models compared to the human experts, we leverage the Dreem Open Dataset-Healthy (DOD-H) and Dreem Open Dataset-Obstructive (DOD-O)^19^. These datasets consist of 80 PSG recordings, each scored by five independent physicians from three different sleep centers following AASM guidelines (version 2.4)^1^.

Table 1 provides an overview of the demographic and dataset statistics, with additional details available in Supplementary Notes: Datasets.

**Table 1:** Overview of the demographic and dataset statistics

ID Dataset	PSGs	Age (y)	%(F/M)	BMI
^13,37^ ABC (*✓*)	132	48.8 ± 9.8	43/57	38.9 ± 3.0
^13,38^ APOE (*✓*)	712	45.7 ± 13.6	41/59	27.2 ± 6.5
^13,39^ APPLES (*✓*)	1094	50.1 ± 12.9	37/63	32.1 ± 7.8
^13,40^ CCSHS (*✓*)	515	17.7 ± 0.4	50/50	25.1 ± 5.9
^13,41^ CFS (*✓*)	730	41.7 ± 20.0	55/45	32.4 ± 9.5
^13,42,43^ CHAT (*✓*)	1638	6.6 ± 1.4	52/48	19.0 ± 4.9
^13,44^ HOMEPAP (*✓*)	246	46.5 ± 11.9	43/57	37.2 ± 8.9
^13,45^ MESA (*✓*)	2056	69.4 ± 9.1	54/46	28.7 ± 5.6
^13,22^ MNC-CNC (*✓*)	78	28.5 ± 16.9	49/51	23.2 ± 11.5
^13,22^ MNC-DHC (*✓*)	83	33.4 ± 14.8	50/50	24.8 ± 4.9
^13,22^ MNC-SSC (*✓*)	767	45.4 ± 13.8	41/59	23.9 ± 6.5
^13,46,47^ MROS (*✓*)	3930	76.4 ± 5.5	0/100	27.2 ± 3.9
^13,48,49^ MSP (*✓*)	105	26.8 ± 5.9	100/0	42.4 ± 6.6
^13,50^ NCHSDB (*✓*)	3950	8.8 ± 5.9	44/56	22.7 ± 9.9
^13,51^ SHHS (*✓*)	8444	63.1 ± 11.2	52/48	28.2 ± 5.1
^13,52,53^ SOF (*✓*)	453	82.8 ± 3.1	100/0	27.7 ± 4.6
^13,54^ WSC (*✓*)	2569	56.4 ± 8.1	46/54	31.7 ± 7.1
**OOD Dataset**	**PSGs**	**Age (y)**	**%(F/M)**	**BMI**
^18^ BSWR	8410	47.9 ± 18.4	34/66	−
^9^ DCSM *✓*	255	−	−	−
^19^ DOD-H *✓*	25	35.3 ± 7.5	24/76	23.8 ± 3.4
^19^ DOD-O *✓*	55	45.6 ± 16.5	36/64	29.6 ± 6.4
^55,56^ PHYS *✓*	994	55.2 ± 14.3	33/67	−
^55,57^ SEDF-SC *✓*	153	58.8 ± 22.0	53/47	−
^55,57^ SEDF-ST *✓*	44	40.2 ± 17.7	68/32	−

Datasets used for in-domain (ID) training (i.e., training, validation and test set split) and out-of-domain (OOD) testing. Missing values are due to study design or anonymized data. Datasets directly available online are identified by *✓*, while datasets that require approval from a Data Access Committee are marked by (*✓*). BSWR is a private dataset.

Below, we outline the experiments conducted in this work, providing readers with a clear overview of the structure and content of the following results:

(i) Benchmarking, ensembling and generalization. We train the sleep staging models on all available NSRR datasets in both single-channel and multi-channel configurations. In Supplementary Table 2 we summarize the data split sets. We then fairly evaluate their performance both on in-domain (ID) datasets and out-of-domain (OOD) datasets (e.g., BSWR). In-domain (ID) datasets refer to data that closely matches the distribution of the training data, while out-of-domain (OOD) datasets come from a different distribution, often representing new or unseen scenarios not encountered during training. We examine how aggregating predictions from multiple models enhances the robustness of sleep stage classification, especially in cases where individual models produce inconsistent results. Given the large number of models in SLEEPYLAND, we focus on the performance achievable using the SOMNUS configuration, which combines predictions from all available model architectures and channel configurations. The experiments provides further insights into the generalizability of the different models, and their consistency across sleep studies. In addition, we explicitly evaluate how exposure to broader, more diverse training data shapes generalization and OOD performance, highlighting the critical role of data diversity in achieving robust generalization.
(ii) Model-bias on performance and clinical markers. As part of our external validation, we apply a previously proposed framework^20,21^ to the large and clinical rich OOD BSWR dataset. This analysis measures how each sleep staging model’s performance and its predictions of clinical markers (such as REM latency) may be biased by demographic factors (like age and gender) and clinical variables (such as AHI and PLMI). Evaluations are based on majority-vote predictions from each sleep staging architecture and their SOMNUS ensemble (details on the experimented models in the subsection below). The framework enables detection of systematic biases (e.g., age- or gender-related) and supports detailed assessment of how performance and prediction errors vary across subpopulations, facilitating comparison of models’ generalizability in real-world clinical settings.
(iii) Model-ensemble versus human-ensemble. We evaluate the SOMNUS ensemble on two OOD multi-scorer datasets, DOD-H and DOD-O^19^, comparing its ability to predict the consensus-derived hypnogram against, (1) hypnogram of individual human scorers, and (2) hypnogram predicted from a baseline model, i.e., SimpleSleepNet^19^, originally trained on the consensus itself. We then assess whether SOMNUS—and its constituent models—tend to align more closely with the consensus than with individual human scorers. This evaluation is conducted both in terms of discrete agreement (Cohen’s *κ*) and similarity between predicted and consensus-derived hypnodensity graphs^22^, quantified exploiting the Averaged Cosine Similarity (ACS) metric^12^. In addition, we compare the degree of agreement among SLEEPYLAND models to that observed among human scorers using the soft-agreement metric proposed by Guillot et al.^19^. Finally, we characterize ensemble variability using the entropy of the soft-voting output and the pairwise divergence between individual model predictions, and investigate their utility as indicators of regions with elevated human scoring uncertainty.

### Benchmarking, ensembling and generalization

In SLEEPYLAND, we evaluate the performance of all model architectures trained in both single-channel and multi-channel configurations. For each model, we predict the full hypnogram of all the PSGs exploiting all the available channel derivations for that record. The ensembling models, i.e., SOMNUS models, are constructed by combining the predicted probabilities from different subsets of model architectures and channel configurations available in SLEEPYLAND, using a soft-voting approach. We consider ensembling across two dimensions: the network architecture and the channel configurations, either considering EEG and EOG derivations jointly, or separately, i.e., using only EEG or only EOG signals. When ensembling is restricted to a fixed architecture or a fixed channel configuration, the corresponding fixed dimension is indicated as a subscript of SOMNUS (e.g., SOMNUS_U-Sleep_ refers to the ensemble of all models based on the U-Sleep architecture, varying only in channel configuration setup). When no subscript is specified, the ensemble incorporates variation across both network architectures and channel configurations. Given the large number of experiments across all the models, we do not show the results for all channel derivations.

In Table 2 we report the performance achievable using the SOMNUS configuration, which combines predictions from all available network architectures and channel configurations. For each ID and OOD dataset we report the mean and standard deviation of F1 scores for each sleep stage and the macro F1 (MF1) score across all stages (computed as the unweighted average of the class-wise F1 scores). The results are reported on a recording-level considering the majority-vote across channel derivations. Overall, the SOMNUS ensemble consistently maintains strong performance across all evaluation settings, achieving macro-F1 scores ranging from 68.7% to 87.2% on ID datasets and from 69.4% to 83.6% on OOD datasets. Although we believe metrics across individual recordings to be the more clinically appropriate, in Supplementary Table 3 we also presents SOMNUS results in the overall (dataset-level) metric format, enabling straightforward comparison with previous and future studies that evaluate performance across all epochs in a dataset rather than on a per-recording basis.

**Table 2:** SOMNUS performance overview

ID Dataset	MF1	W	N1	N2	N3	REM
ABC	78.9 ± 8.7	89.5 ± 4.5	60.5 ± 8.7	86.6 ± 6.8	66.6 ± 28.8	89.5 ± 20.1
APOE	72.7 ± 10.4	87.7 ± 7.6	43.8 ± 18.0	85.5 ± 9.9	57.6 ± 32.1	86.5 ± 13.6
APPLES	74.0 ± 9.3	91.2 ± 5.1	50.6 ± 15.7	84.6 ± 13.5	36.1 ± 32.5	87.4 ± 15.0
CCSHS	87.2 ± 4.9	97.3 ± 1.7	64.9 ± 14.1	91.9 ± 4.8	88.1 ± 9.4	93.8 ± 3.5
CFS	81.7 ± 8.0	96.3 ± 3.9	53.9 ± 16.2	88.8 ± 9.9	78.1 ± 22.3	91.6 ± 8.2
CHAT	84.2 ± 4.3	96.0 ± 3.1	58.9 ± 11.4	85.8 ± 7.8	89.9 ± 7.2	90.1 ± 5.7
HOMEPAP	75.5 ± 8.3	90.8 ± 7.8	41.6 ± 15.5	82.7 ± 9.1	74.4 ± 25.3	90.4 ± 11.1
MESA	75.2 ± 9.2	95.3 ± 6.1	51.0 ± 14.6	85.2 ± 11.1	53.6 ± 29.7	90.6 ± 7.1
MNC-CNC	78.6 ± 6.1	80.5 ± 15.0	53.6 ± 13.0	83.8 ± 10.2	86.2 ± 6.7	89.1 ± 4.0
MNC-DHC	81.3 ± 6.1	97.8 ± 1.4	51.6 ± 14.4	85.1 ± 8.3	82.2 ± 9.1	89.7 ± 5.4
MNC-SSC	68.7 ± 10.7	80.7 ± 12.6	31.2 ± 17.3	86.3 ± 6.6	59.2 ± 31.4	85.5 ± 16.8
MROS	75.2 ± 8.4	95.8 ± 3.5	45.9 ± 16.1	88.0 ± 5.5	57.5 ± 29.1	88.0 ± 14.3
MSP	78.7 ± 7.6	92.8 ± 5.6	52.0 ± 9.7	88.8 ± 5.3	68.0 ± 26.4	89.4 ± 11.5
NCHSDB	75.9 ± 7.5	86.0 ± 10.6	33.5 ± 16.5	85.6 ± 12.6	89.8 ± 9.4	85.3 ± 14.6
SHHS	77.9 ± 8.8	93.5 ± 7.5	46.1 ± 20.7	87.3 ± 7.6	72.5 ± 21.7	90.4 ± 8.9
SOF	77.1 ± 7.1	95.1 ± 4.7	41.6 ± 15.8	85.6 ± 8.0	71.0 ± 19.3	92.8 ± 7.1
WSC	74.0 ± 9.8	89.5 ± 10.6	50.0 ± 16.6	90.5 ± 5.6	48.6 ± 29.0	88.4 ± 13.9
**OOD Dataset**	**MF1**	**W**	**N1**	**N2**	**N3**	**REM**
BSWR	71.6 ± 11.2	83.4 ± 14.0	41.0 ± 17.1	81.1 ± 12.4	66.7 ± 28.2	86.8 ± 16.9
DCSM	80.3 ± 8.3	98.3 ± 2.4	50.0 ± 15.1	85.7 ± 9.7	78.6 ± 19.6	89.5 ± 14.4
DOD-H	83.6 ± 6.6	89.3 ± 9.4	57.2 ± 17.1	91.5 ± 3.8	85.7 ± 16.3	94.2 ± 4.8
DOD-O	79.2 ± 7.9	92.1 ± 5.6	52.6 ± 13.5	89.2 ± 6.2	70.3 ± 27.6	92.7 ± 7.0
PHYS	69.4 ± 9.7	74.4 ± 16.0	38.7 ± 15.6	83.7 ± 10.2	66.0 ± 26.7	85.5 ± 16.6
SEDF-SC	74.0 ± 8.1	98.4 ± 1.2	37.5 ± 14.0	83.3 ± 8.2	61.0 ± 28.1	88.0 ± 8.5
SEDF-ST	75.6 ± 7.6	79.3 ± 10.8	48.1 ± 15.3	87.2 ± 5.7	73.3 ± 24.3	90.2 ± 7.9
**Avg ID**	77.5 ± 4.5	91.5 ± 5.4	48.9 ± 8.9	86.6 ± 2.4	69.4 ± 15.7	89.3 ± 2.3
**Avg OOD**	76.2 ± 5.1	87.9 ± 9.3	46.4 ± 7.5	86.0 ± 3.6	71.7 ± 8.4	89.6 ± 3.1

MF1 (%) and Class-Wise F1 score (%) on recording-level for soft unweighted ensemble of all models (majority-vote across channel derivations).

To contextualize these outcomes, in Table 3 we compare SOMNUS with state-of-the-art results from automatic sleep scoring methods^9,10,16,22–25^. In these comparisons, we select the performance metric that each original study reported as their primary measure—i.e., dataset-level or recording-level accuracy, Cohen’s Kappa, and macro-F1—thereby ensuring a fair evaluation. SOMNUS consistently meets or surpasses these established methods, providing strong performance in cross-dataset (out-of-domain) testing scenarios, demonstrating its superior generalization capability to previously unseen data. Even in comparisons where the state-of-the-art models had been trained directly on a specific dataset (in-domain) while SOMNUS treated it as out-of-domain, SOMNUS often matches or closely approaches their performance. These gains are primarily driven by the large amount and diversity of training data, combined with the unified training framework used in SLEEPYLAND. In addition, the complementary predictions from heterogeneous network architectures and channel configurations contribute to more stable and robust performance across sleep stages.

**Table 3:** Comparison of SOMNUS with state-of-the-art automatic sleep scoring methods

Model	Dataset	Metric	SOTA vs SOMNUS
YASA^23^	CCSHS^⋆^	Acc	0.90 (*✓*) vs **0.93** (*✓*)
	MESA^⋆^	Acc	0.84 (*✓*) vs **0.89** (*✓*)
	DOD-H^•^	Acc	0.87 (✗) vs **0.91** (✗)
		MF1	0.79 (✗) vs **0.84** (✗)
	DOD-O^•^	Acc	0.84 (✗) vs **0.89** (✗)
		MF1	0.74 (✗) vs **0.81** (✗)
U-Sleep^9^	ABC^⋆^	MF1	0.77 (*✓*) vs **0.83** (*✓*)
	CCSHS^⋆^	MF1	0.85 (*✓*) vs **0.88** (*✓*)
	CFS^⋆^	MF1	0.82 (*✓*) vs **0.83** (*✓*)
	CHAT^⋆^	MF1	**0.85** (*✓*) vs **0.85** (*✓*)
	DCSM^⋆^	MF1	0.81 (*✓*) vs **0.82** (✗)
	HOMEPAP^⋆^	MF1	**0.78** (*✓*) vs **0.78** (*✓*)
	MESA^⋆^	MF1	**0.79** (*✓*) vs **0.79** (*✓*)
	MROS^⋆^	MF1	0.77 (*✓*) vs **0.79** (*✓*)
	PHYS^⋆^	MF1	**0.79** (*✓*) vs 0.73 (✗)
	SEDF-SC^⋆^	MF1	**0.79** (*✓*) vs 0.75 (✗)
	SEDF-ST^⋆^	MF1	0.76 (*✓*) vs **0.78** (✗)
	SHHS^⋆^	MF1	**0.80** (*✓*) vs **0.80** (*✓*)
	SOF^⋆^	MF1	0.78 (*✓*) vs **0.79** (*✓*)
	DOD-H^⋆^	MF1	0.82 (✗) vs **0.86** (✗)
	DOD-O^⋆^	MF1	0.79 (✗) vs **0.82** (✗)
Stephansen et al.^22^	WSC^•^	Acc	**0.86** (*✓*) vs **0.86** (*✓*)
	DOD-H^•^	Acc	0.86 (✗) vs **0.91** (✗)
		MF1	0.79 (✗) vs **0.84** (✗)
	DOD-O^•^	Acc	0.85 (✗) vs **0.89** (✗)
		MF1	0.70 (✗) vs **0.81** (✗)
DeepResNet^10^	MROS^⋆^	Acc	0.87 (*✓*) vs **0.90** (*✓*)
		Kappa	0.79 (*✓*) vs **0.85** (*✓*)
	SHHS^⋆^	Acc	0.87 (*✓*) vs **0.88** (*✓*)
		Kappa	0.81 (*✓*) vs **0.83** (*✓*)
	WSC^⋆^	Acc	**0.86** (*✓*) vs **0.86** (*✓*)
		Kappa	0.77 (*✓*) vs **0.79** (*✓*)
	SSC^⋆^	Acc	0.81 (*✓*) vs **0.82** (*✓*)
		Kappa	0.70 (*✓*) vs **0.72** (*✓*)
SleepTransformer^16^	SHHS^⋆^	Acc	**0.88** (*✓*) vs **0.88** (*✓*)
		MF1	**0.80** (*✓*) vs **0.80** (*✓*)
		Kappa	**0.83** (*✓*) vs **0.83** (*✓*)
	SEDF-SC^⋆^	Acc	0.85 (*✓*) vs **0.92** (✗)
		MF1	**0.79** (*✓*) vs 0.75 (✗)
		Kappa	0.79 (*✓*) vs **0.84** (✗)
L-SeqSleepNet^24^	SHHS^⋆^	Acc	**0.88** (*✓*) vs **0.88** (*✓*)
		MF1	**0.80** (*✓*) vs **0.80** (*✓*)
		Kappa	**0.83** (*✓*) vs **0.83** (*✓*)
SleepFM^25^	MESA^⋆^	MF1	0.78 (*✓*) vs **0.79** (*✓*)
	MROS^⋆^	MF1	0.75 (*✓*) vs **0.79** (*✓*)
	SHHS^⋆^	MF1	0.78 (*✓*) vs **0.80** (*✓*)

^*^ denotes dataset-wise (overall) evaluation, and ^•^ denotes recording-wise evaluation. The last column reports results for each state-of-the-art (SOTA) models or SOMNUS model, with in-domain status indicated by *✓*(dateset used during training) or ✗(dataset not used during training). Best results for each row are in bold. *Note:* SOMNUS metrics are computed using the same aggregation (mean, median, etc.) as reported in the original publication of the compared SOTA model; inconsistencies with SOMNUS values in other tables of this manuscript are due to this adaptation.

Due to space constraints, the evaluations of the individual models, changing in network architecture and channel configurations, are instead reported in Supplementary Tables 4–19.

Overall, SOMNUS achieves higher median recording-wise macro F1 performance than individual models (i.e., specific architecture and channel configuration) in 94.9% of cases. Among these, it is significantly better in 72.7% of comparisons, and in the remaining cases, it is never significantly worse (Wilcoxon one-sided paired test, adjusted for multiple comparisons). This advantage is further illustrated in Supplementary Fig. 9, which displays the distribution of median performance differences between SOMNUS and individual models across datasets. Additionally, Supplementary Table 22 reports similar per-sleep-stage analyses, showing that SOMNUS consistently outperforms individual models across all sleep stages.

#### Achieving and assessing true generalization

The amount and diversity of training data are central to enhancing the generalization capabilities of sleep staging models^8,10,22^. To confirm this within our unified framework, where other potential confounders such as data splits, preprocessing, training procedures, and data sampling strategy are held constant, we compare two versions of SOMNUS: one trained on all the NSRR datasets and another trained on a reduced subset, excluding APOE, APPLES, MNC, MSP, NCHSDB, and WSC. The exclusion is based purely on the temporal sequence of data availability—these datasets were added to NSRR after 2022 and treated as additional sources not included in earlier analyses. As shown in Supplementary Fig. 10, the more comprehensively trained SOMNUS version achieves better generalization on 5 out of 7 OOD datasets and improved performance on newly included NSRR datasets, although with a slight drop in performance on the shared training datasets, likely reflecting their reduced representation within the extended training pool. This pattern is further supported by results reported in Supplementary Table 20 (and clearly visible in the EEG-only subplots in Supplementary Figs. 1–2), where an EEG-only SleepTransformer model (typically among the best performing individual models in our benchmarking) trained exclusively on SHHS fails entirely to generalize to other datasets. Its median macro-F1 score approaches that of the counterpart trained on the full NSRR data only for SHHS itself, with substantial and sometimes dramatic performance declines for other datasets, particularly in OOD evaluations.

Together, these insights reinforce a crucial point: *achieving and assessing true generalization depends on training and evaluating models across increasingly larger and more diverse PSG datasets. Single-dataset training and evaluation, even with well-designed architectures, do not provide meaningful insights into true generalizability*.

### Model-bias on performance and clinical markers

#### BSWR: demographics and clinical variables

For a detailed assessment of robustness of individual sleep staging architectures, we exploit the Generalized Additive Models for Location, Scale, and Shape (GAMLSS) framework^20,21^, applied on the BSWR clinical database. The evaluation is conducted on a subset of $6{\prime} 633$ recordings with complete information for bias-inducing covariates *X*, i.e., age, gender, apnea-hypopnea index (AHI), periodic limb movement index (PLMI), and the relevant performance/markers outcomes. This subset has a mean age of 47.9 years (*SD* = 18.2; range: 0−88), with females representing 35.1% of the sample. The mean AHI is 17.7 events/hour (*SD* = 19.2; range: 0 − 141), and the mean PLMI is 12.1 events/hour (*SD* = 21.9; range: 0−204). Based on two-sided t-test, females are younger (mean difference: −4.1; 95% *CI*: [ − 5.02, −3.16]), have lower AHI (mean difference: − 8.6; 95% *CI*: [ − 9.48, − 7.73]), and also have lower PLMI (mean difference: − 5.0; 95%*CI*: [ − 5.99, − 4.01]), in comparison to males. Using Pearson’s coefficient, age shows moderate positive correlations with AHI (*r* = 0.30, 95% *CI*: [0.28, 0.33]) and PLMI (*r* = 0.30, 95% *CI*: [0.28, 0.32]), while AHI and PLMI are weakly correlated (*r* = 0.056, 95% *CI*: [0.032, 0.080]).

#### Age, gender, AHI and PLMI bias

**Fig. 1:**
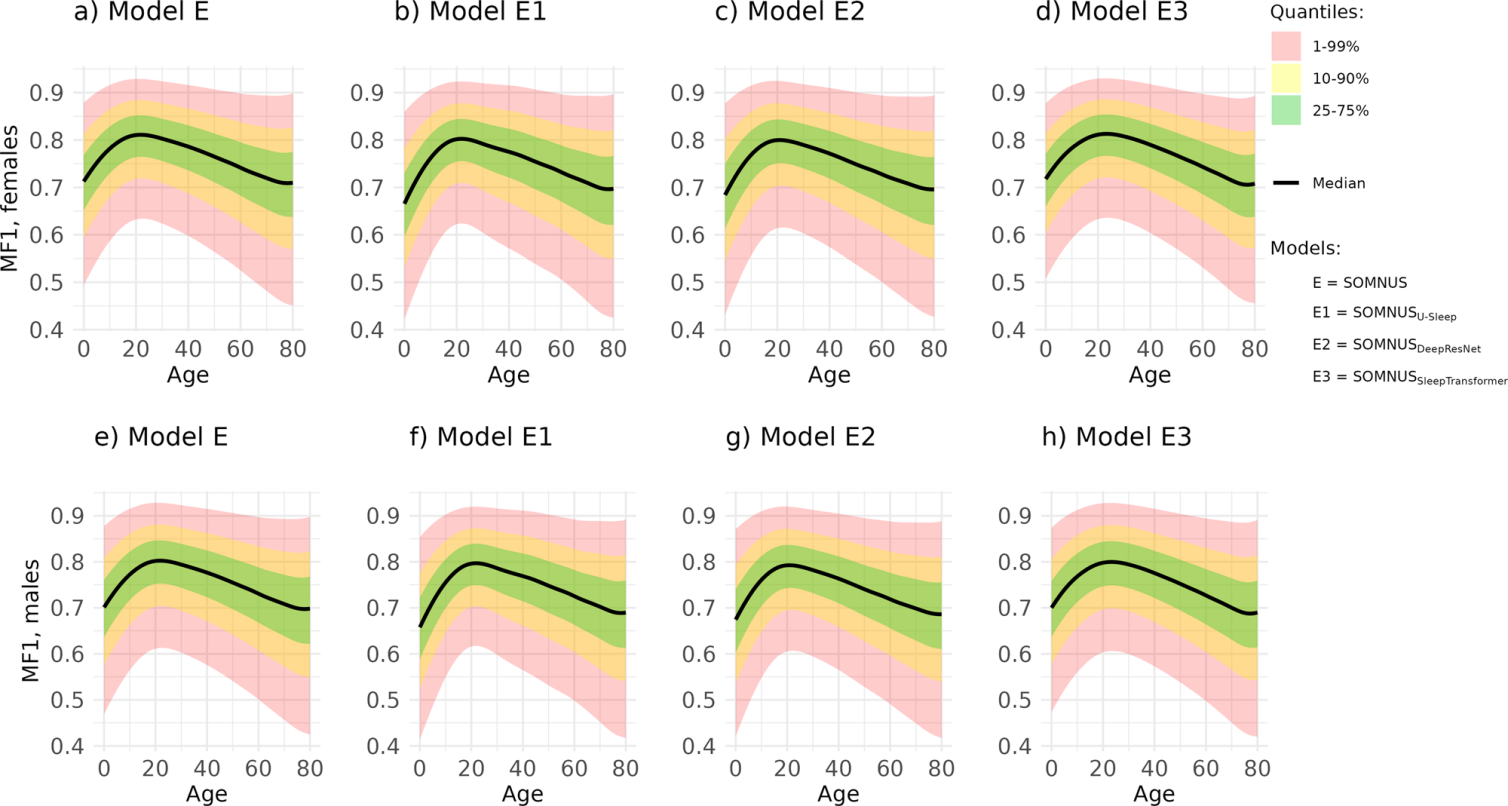
Age-conditioned expected distributions of MF1-scores quantiles for males and females. Age-conditioned expected distributions of MF1-scores quantiles for females (**a**–**d**) and males (**e**–**h**) (under an optimistic scenario of AHI = PLMI = 0) across four sleep staging models, i.e., SOMNUS (Model E), SOMNUS_U--Sleep_ (Model E1), SOMNUS_DeepResNet_ (Model E2), and SOMNUS_SleepTransformer_ (Model E3).

In Table 4 we report the bias/effects of the predictors *X*, i.e., age, gender, AHI, PLMI, on both the average (location parameter, *μ*) and variability (scale parameter, *σ*) of the outcomes. Specifically, we consider model performance metrics—MF1 and Class-Wise F1 scores—and clinically relevant sleep markers such as total sleep time (TST) and wake after sleep onset (WASO). The effect of each variable is modeled as a linear term in the distributional parameters (except for age), while the parameters for the *inflated Beta distributions* (*ν*, *τ*) are kept constant. To clarify, the inflated Beta distribution allows us to appropriately model our outcomes, including the possibility of values exactly at the boundaries (0 or 1), by accounting for excess zeros or ones in the data; in this context, it helps capture rare cases of perfect or failed predictions. We identified which variables were most important for each outcome in the model. In the results tables, the presence of a numeric value indicates that the corresponding variable—i.e., gender, AHI, or PLMI—was retained in the final model for that parameter; blank cells indicate that the variable was excluded. Bold values flag the best outcomes—either higher performance, lower variability, or bias correction. If a predictor/covariate has no detectable effect on a metric, that setting is considered unbiased (and therefore most desirable). We do not report the age related predictor (modeled exploiting a cubic spline—see Fig. 1), mainly because a significant non-linear effect on *μ* and *σ* is always identified for all the performance and clinical markers.

**Table 4:** Bias on performance metrics and on prediction-derived clinical markers

		(Intercept)				Gender				AHI				PLMI			
		E	E1	E2	E3	E	E1	E2	E3	E	E1	E2	E3	E	E1	E2	E3
MF1	*μ*	1.12	1.06	1.05	**1.13**	−0.06	**−0.03**	−0.04	−0.09	−0.05	−0.05	−0.05	−0.05	−0.02	−0.02	−0.02	−0.02
	*σ*	−1.51	−1.46	−1.47	**−1.53**	0.06			0.08					0.01	0.01		0.02
	*ν*	**−22.54**	**−22.54**	**−22.54**	−21.54												
	*τ*	−22.63	−22.63	−22.63	**−21.63**												
F1_W_	*μ*	**1.93**	**1.93**	1.90	1.90	−0.24	**−0.23**	−0.24	−0.24	−0.07	**−0.06**	−0.07	−0.07	0.01	0.01	0.01	0.01
	*σ*	−0.97	−0.98	**−0.99**	−0.96	0.18	0.20	0.19	**0.17**	0.02	**0.01**	0.02	0.03				
	*ν*	**−8.80**	−8.11	**−8.80**	**−8.80**												
	*τ*	−23.63	−24.49	−24.49	**−8.80**												
F1_N1_	*μ*	−0.28	**−0.22**	−0.26	−0.39		0.03	0.03		−0.03	**−0.02**	**−0.02**	−0.04	−0.02	−0.02	**−0.01**	−0.02
	*σ*	−0.75	−0.79	**−0.83**	−0.72	**−0.05**	−0.03	−0.04	−0.04				0.02				
	*ν*	−4.82	−5.10	**−5.58**	−4.03												
	*τ*	−22.63	−24.17	**−20.63**	−23.62												
F1_N2_	*μ*	**1.68**	1.59	1.58	1.67		0.04	0.04		−0.09	−0.09	−0.09	−0.09	−0.03	−0.03	**−0.02**	−0.04
	*σ*	−1.35	−1.31	−1.32	**−1.36**					**0.05**	**0.05**	**0.05**	0.06	0.02	**0.01**	**0.01**	0.03
	*ν*	**−24.47**	−23.54	−24.14	−24.14												
	*τ*	−24.49	**−23.63**	−24.18	−24.18												
F1_N3_	*μ*	1.03	0.78	0.76	**1.25**	−0.14	**−0.05**	−0.09	−0.20	−0.03	−0.03	−0.03	−0.03	**−0.02**	−0.03	−0.03	−0.03
	*σ*	−0.34	−0.21	−0.22	**−0.47**	0.10			0.15	0.02	0.02	0.02	**0.01**	**0.01**	**0.01**	**0.01**	0.02
	*ν*	−2.88	−2.64	−2.71	**−3.10**												
	*τ*	−8.75	−7.63	−24.13	**−7.37**												
F1_REM_	*μ*	**2.09**	2.03	2.04	2.00					−0.05	**−0.04**	−0.05	**−0.04**				
	*σ*	**−0.95**	−0.91	−0.94	−0.85		0.05		**−0.04**	0.02	**0.01**	0.02	**0.01**	**0.04**	**0.04**	0.05	**0.04**
	*ν*	−4.06	−4.16	**−4.23**	−4.03												
	*τ*	−4.65	−4.77	−5.07	**−4.60**												
TST	*μ*	−9.05	**−8.11**	−8.98	−9.96	−1.37	**−0.99**	−1.35	−1.84	−1.95	**−1.69**	−1.77	−2.28	−0.47			−0.50
	*σ*	**2.86**	2.88	2.88	2.88	**0.08**	0.10	**0.08**	0.10	0.03	**0.02**	0.04	0.04	0.04	0.04	**0.03**	0.04
WASO	*μ*	5.70	**5.52**	5.87	6.23	1.91	**1.73**	1.94	2.22	1.53	**1.24**	1.42	1.77				
	*σ*	2.78	2.82	2.80	**2.77**	**0.08**	0.09	0.09	0.10	0.06	**0.05**	0.06	0.07	0.04	**0.03**	**0.03**	0.04
N1	*μ*	−27.54	**−25.77**	−25.81	−28.76	−1.70	**−1.25**	−1.47	−2.06	−5.91	**−5.47**	−5.56	−6.30	−1.53	−1.40	**−1.35**	−1.65
	*σ*	3.03	3.01	**3.00**	3.07					0.10	0.10	0.10	0.10	0.05	0.05	**0.04**	**0.04**
N2	*μ*	36.55	43.38	43.47	**22.68**	−3.77	**−5.37**	−4.34	−1.99	3.68	3.10	**2.99**	4.33	1.09	**0.89**	1.01	1.17
	*σ*	3.39	3.46	3.45	**3.36**			−0.03		**0.05**	**0.05**	**0.05**	0.06	0.01		0.01	0.02
N3	*μ*	−22.69	−28.76	−30.13	**−9.59**	5.54	**7.09**	6.37	3.27	0.44	0.72	**0.79**		0.17	0.23	0.19	**0.25**
	*σ*	**3.26**	3.34	3.32	3.29	−0.05	**−0.07**	**−0.07**	−0.03	**−0.04**	**−0.04**	**−0.04**	−0.03	0.01	0.01		0.01
REM	*μ*	4.33	**2.60**	3.21	5.61	−0.65		**−0.88**	−0.73	−0.19			**−0.27**	**−0.37**	−0.32	−0.29	−0.42
	*σ*	**2.42**	2.43	2.39	2.47	−0.03	−0.03	**−0.04**	**−0.04**			0.01	−0.01	0.02	0.02	0.02	0.02
REML	*μ*	1.80	−1.01	**−0.57**	0.78		1.74			1.10	0.50	0.80	0.71	0.35			0.93
	*σ*	3.76	3.88	3.76	**3.75**		**−0.09**	0.05		**0.03**	0.04	0.04	0.04	**0.02**	0.03	**0.02**	0.03
AwH	*μ*	0.32	**0.25**	0.34	0.40	0.23	**0.21**	0.23	0.24	0.08	**0.06**	0.07	0.10	−0.02	−0.02	−0.02	**−0.03**
	*σ*	**0.03**	0.05	**0.03**	0.05	0.15	0.14	**0.13**	0.17	0.12	**0.11**	0.12	0.12	0.01	0.01	0.01	
TrH	*μ*	−4.93	−4.65	**−4.48**	−4.76	**0.41**	0.32	**0.41**	0.36	−0.18	**−0.06**	−0.14	−0.10	0.09	**0.13**	0.07	0.01
	*σ*	1.76	1.82	1.79	**1.72**				0.03	0.12	0.12	0.12	0.12	0.03	0.03	0.03	0.03

Gender, AHI, and PLMI modeled as predictors of performance metrics for four sleep staging models, i.e., SOMNUS (**E**), SOMNUS_U–Sleep_ (**E1**), SOMNUS_DeepResNet_ (**E2**), and SOMNUS_SleepTransformer_ (**E3**)—exploiting the GAMLSS framework. Gender, AHI, and PLMI entered as linear terms. Bias estimated for the location (*μ*, expectation) and scale (*σ*, variability) parameters, while the inflated–Beta shape parameters (*ν*, *τ*) fixed at their intercepts. Bold values flag the best outcomes—either higher performance, lower variability, or bias correction.

In this section, for simplicity, we will refer to the four architecture dependent ensemble sleep staging models—for which we quantify the bias—as follows: SOMNUS (*E*), SOMNUS_U−Sleep_ (*E*1), SOMNUS_DeepResNet_ (*E*2), and SOMNUS_SleepTransformer_ (*E*3).

Performance of all sleep staging models at baseline (defined as a 50−year-old female with AHI = PLMI = 0) is comparable, with macro-F1 scores, ranging from 0.74 to 0.76 the latter also exhibiting the highest consistency (i.e., lowest variability). Probabilities of complete misclassification or perfect scoring are negligible across models, with log-inflation parameters near − 22, and corresponding probabilities < 10^−9^. Performance and precision decrease with increasing AHI, PLMI, and male gender, with *E*3 showing the largest gender-related MF1 drop. Stage-wise F1-scores are highest for REM and wake ( ≈ 0.87−0.89), followed by N2 ( ≈ 0.83−0.84), N3 ( ≈ 0.68−0.78), and N1 ( ≈ 0.40−0.45), which also shows the most variability. *E*3 yields the best N3 performance and matches others on REM and N2. Gender especially affects wake and N1 variability, while AHI and PLMI amplify variability in N2, N3, and REM. Across age, MF1 and F1 for N1, N2, and N3 (in older adults) decline at both ends of the lifespan, with REM and N3 showing wide uncertainty bands (refer to Fig. 1, and Supplementary Figs. 3−4).

Sleep parameters prediction (i.e., TST, WASO, and REM duration) by all models at baseline (defined as a 50-year-old female with AHI = PLMI = 0) shows limited bias, with absolute deviations < 10 min. TST is slightly underestimated (−10 to − 8 min), WASO overestimated ( + 5 to + 6 min), i.e., likely linked to higher sensitivity of sleep staging models in detecting (micro)arousals, and REM modestly overestimated (+2 to +6 min), leading to consistent underestimation of sleep efficiency. Biases are more pronounced in stage durations: N1 and N2 are overestimated (N1: + 26 − 29 min; N2: + 23 − 43 min), while N3 is underestimated ( − 30 min in DeepResNet; − 10 min in SleepTransformer). REM latency (REML) shows small, model-dependent fluctuations. All models underestimate stage transitions (TrH: − 4.5 to − 5 /h) and overestimate awakenings (AwH: + 0.25 to + 0.40 /h), suggesting oversmoothing and sensitivity to microarousals. Based on GAMLSS, *E*1 performs best for TST, WASO, N1, REM, and AwH; *E*2 for REML and TrH; and *E*3 for N2 and N3. *E* seems to not minimize any baseline bias.

Increased AHI and PLMI generally worsen bias and variability but partially correct underestimation in N3 and REM due to their natural reduction in sleep-disordered individuals. Male gender amplifies most biases, but reduces N3 and REM errors, reflecting naturally lower amounts in older males. Age-related trends are non-linear and consistent across models (refer to Supplementary Figs. 5–8). WASO is underestimated in children and overestimated in older adults. N1 is increasingly underestimated at age extremes, N2 is overestimated in middle age, N3 is underestimated in youth and REM is slightly overestimated in children. Overall, variability rises in children and elderly, especially for N3 and REM. As a result, TST is slightly overestimated in children and underestimated in adults, compensating for WASO biases. REM latency remains generally stable with increased variability at age extremes. Awakenings are overestimated and transitions underestimated, especially in middle-aged subjects, with variability sharply increasing in older adults. These trends likely reflect age-related changes in sleep depth and fragmentation. No model architecture consistently minimizes bias across markers.

### Model-ensemble versus human-ensemble

Evaluating automatic sleep staging algorithms against human experts requires careful consideration of the intrinsic variability between scorers. Disagreements among trained sleep technologists are well-documented^26^, particularly at transitions between sleep stages^22^, and any robust automatic method must demonstrate consistent performance in the face of such ambiguities. Notably, medical consensus consistently outperforms individual expert judgment^27^, indicating that reliable models should aim to replicate consensus-based scoring rather than emulate any single scorer’s decisions^12^. To investigate this aspect in the context of SLEEPYLAND models, we leverage the multi-scored Dreem Open Datasets introduced by Guillot et al.^19^. These datasets include polysomnographic recordings from 25 healthy individuals (DOD-H) and 55 patients diagnosed with obstructive sleep apnea (DOD-O), each independently annotated by five experienced sleep technologists from different clinical centers.

#### Benchmarking against human experts and SimpleSleepNet

**Fig. 2:**
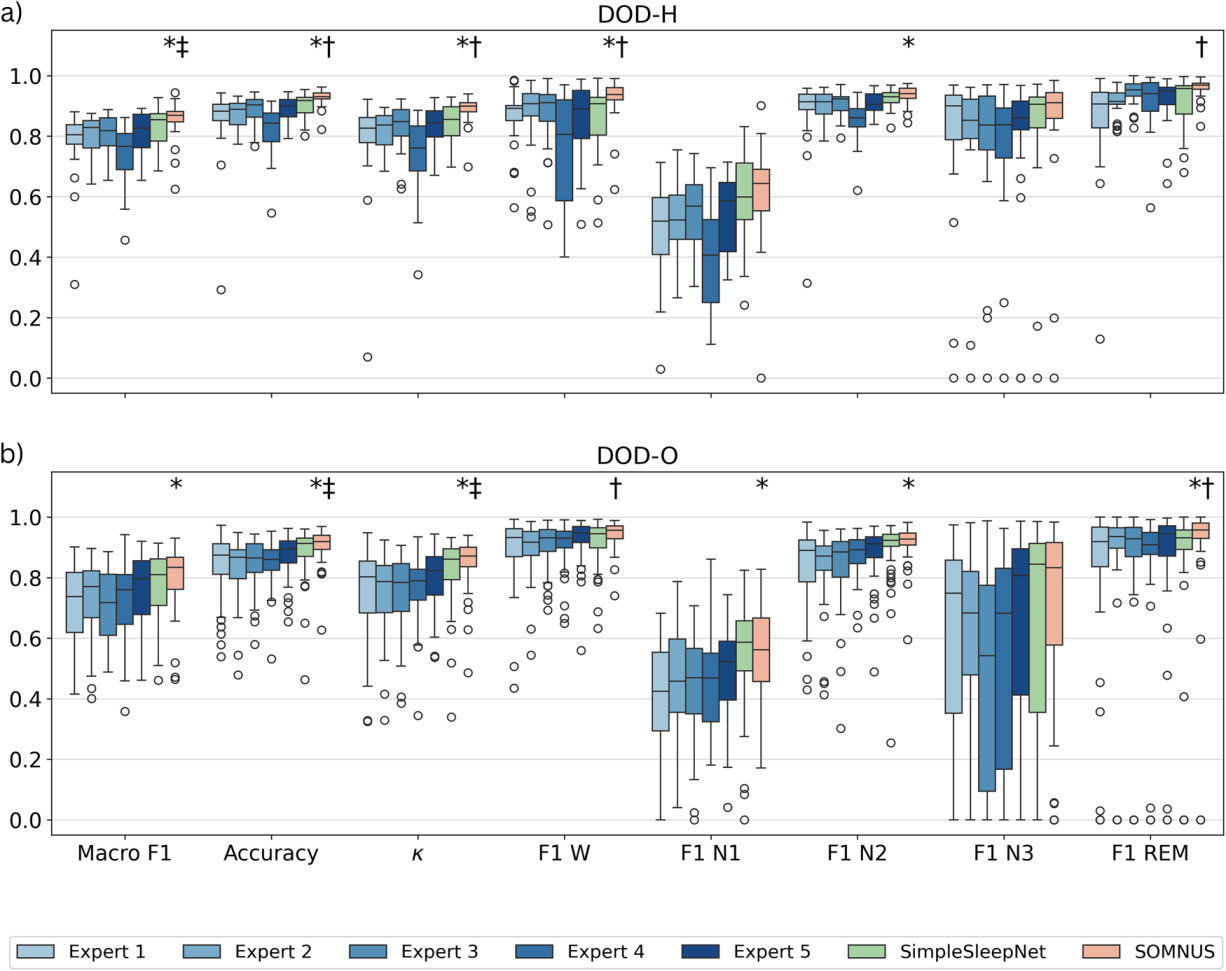
Distributions of recording-wise SOMNUS performance metrics for the OOD multi-scored dataset, relative to consensus labels. Boxplots show the distributions of recording-wise performance metrics for the DOD-H (**a**) and DOD-O (**b**) datasets. Results are shown with respect to consensus annotations. Statistical significance markers above each boxplot indicate superior performance of SOMNUS: *: *p*_*adj*_ < 0.05 vs all experts, †: *p*_*adj*_ < 0.05 vs SimpleSleepNet, ‡: 0.05≤*p*_*adj*_ < 0.10 vs SimpleSleepNet.

We evaluate SOMNUS, our ensemble of high-performing models, against two benchmarks: (i) the individual human scorers, along with their average performance, and (ii) SimpleSleepNet, a model originally developed and trained specifically on DOD-H and DOD-O^19^. SOMNUS was never trained on DOD-H or DOD-O, making this a true out-of-domain evaluation that tests its robustness to inter-scorer variability. In Table 5 we report average performance across metrics, whilst in Fig. 2 we illustrate the distribution of recording-level performance metrics for both datasets. Evaluation protocols closely follow those defined in Guillot et al.^19^.

**Table 5:** Performance comparison with expert consensus on DOD-H and DOD-O datasets

	Scorer	MF1	*κ*	W	N1	N2	N3	REM
DOD-H	Expert 1	77.8 ± 11.6	78.7 ± 16.3	86.2 ± 9.7	49.2 ± 14.4	87.8 ± 12.6	80.5 ± 24.3	85.0 ± 17.2
	Expert 2	80.1 ± 6.7	81.5 ± 6.5	86.7 ± 12.1	52.2 ± 11.4	90.3 ± 4.6	79.9 ± 22.9	91.2 ± 4.7
	Expert 3 (Best)	80.8 ± 6.1	83.0 ± 7.6	87.7 ± 10.6	55.2 ± 13.3	90.5 ± 4.4	76.4 ± 25.1	94.4 ± 4.2
	Expert 4	73.7 ± 10.6	73.3 ± 12.9	75.2 ± 17.9	40.3 ± 16.5	84.7 ± 6.9	76.8 ± 21.8	91.6 ± 8.9
	Expert 5	80.4 ± 7.5	82.8 ± 7.2	85.6 ± 12.0	54.7 ± 11.8	91.1 ± 3.7	78.9 ± 24.8	91.8 ± 8.0
	Experts (Avg)	78.6 ± 9.2	79.9 ± 11.4	84.3 ± 13.6	50.3 ± 14.7	88.9 ± 7.6	78.5 ± 23.9	90.8 ± 10.3
	SimpleSleepNet	82.4 ± 7.1	84.6 ± 6.5	86.1 ± 11.5	59.8 ± 14.4	92.4 ± 3.1	82.5 ± 23.0	91.4 ± 8.3
	SOMNUS	**85.2** ± **6.6**	**88.9** ± **4.7**	**91.8** ± **7.7**	**60.9** ± **16.9**	**93.5** ± **3.2**	**84.1** ± **22.7**	**95.8** ± **3.4**
DOD-O	Expert 1	71.4 ± 12.7	74.9 ± 15.3	89.7 ± 10.4	41.0 ± 16.7	83.8 ± 12.9	60.0 ± 32.1	82.6 ± 25.9
	Expert 2	73.5 ± 11.9	75.3 ± 12.2	89.5 ± 8.3	45.8 ± 16.1	84.0 ± 11.4	60.4 ± 29.7	87.9 ± 21.7
	Expert 3	70.6 ± 11.4	75.4 ± 12.4	90.9 ± 7.2	44.5 ± 16.5	84.5 ± 12.0	46.6 ± 33.3	86.5 ± 21.8
	Expert 4	72.2 ± 12.1	76.4 ± 10.6	91.0 ± 7.3	44.6 ± 15.2	87.4 ± 6.7	53.7 ± 33.5	84.3 ± 24.0
	Expert 5 (Best)	75.9 ± 11.4	80.5 ± 9.5	92.7 ± 6.9	48.5 ± 15.3	88.3 ± 8.5	63.7 ± 33.9	86.5 ± 22.4
	Experts (Avg)	72.7 ± 12.1	76.5 ± 12.3	90.8 ± 8.2	44.9 ± 16.2	85.6 ± 10.7	56.9 ± 33.1	85.6 ± 23.3
	SimpleSleepNet	77.6 ± 11.4	82.3 ± 11.2	91.7 ± 7.4	**55.4** ± **16.8**	89.7 ± 10.5	64.8 ± 36.0	86.5 ± 22.5
	SOMNUS	**80.2** ± **10.1**	**85.4** ± **8.0**	**94.3** ± **4.3**	55.3 ± 13.7	**91.6** ± **5.9**	**68.5** ± **32.0**	**91.1** ± **18.6**

MF1 (%) and Class-Wise F1 score (%) on recording-level of the experts and of the models on DOD-H and DOD-O with respect to the soft-consensus among the experts. Recording-level mean ± standard deviation of MF1 (%) and Class-Wise F1 score (%), and Cohen’s *κ* for individual human scorers, their average (Avg), SimpleSleepNet, and SOMNUS on DOD-H and DOD-O. All metrics are computed with respect to the expert-derived consensus. Best results for each metric and dataset are in bold.

**DOD-H**. SOMNUS significantly outperforms all human experts across all global metrics: Macro F1 score (*p*_*adj*_ = 0.002, *r* = 0.70), Accuracy (*p*_*adj*_ < 0.001, *r* = 0.82), Cohen’s *κ* (*p*_*adj*_ < 0.001, *r* = 0.82), as well as for the staging of Wake (*p*_*adj*_ = 0.008, *r* = 0.64) and N2 (*p*_*adj*_ = 0.020, *r* = 0.59). No significant differences are observed for N1, N3, or REM sleep, although small to moderate effect sizes consistently favor SOMNUS. When compared to SimpleSleepNet, SOMNUS achieves significantly higher Accuracy (*p*_*adj*_ = 0.025, *r* = 0.57), Cohen’s *κ* (*p*_*adj*_ = 0.020, *r* = 0.59), and improved classification of Wake (*p*_*adj*_ = 0.008, *r* = 0.64) and REM stages (*p*_*adj*_ = 0.030, *r* = 0.55). For Macro F1 score, SOMNUS is marginally superior (*p*_*adj*_ = 0.096, *r* = 0.44). No significant differences are found for NREM stages, though median performances again trend in favor of SOMNUS.

**DOD-O**. SOMNUS consistently outperforms all individual human experts in terms of Macro F1 score (*p*_*adj*_ < 0.001, *r* = 0.53), Accuracy (*p*_*adj*_ < 0.001, *r* = 0.54), Cohen’s *κ* (*p*_*adj*_ < 0.001, *r* = 0.52), and in the determination of N1 (*p*_*adj*_ = 0.001, *r* = 0.50), N2 (*p*_*adj*_ < 0.001, *r* = 0.55), and REM sleep (*p*_*adj*_ < 0.001, *r* = 0.51). No significant differences are found for the Wake and N3 stages. When compared to SimpleSleepNet, SOMNUS achieves slightly higher overall performance, although the differences are only weakly significant for both Accuracy (*p*_*adj*_ = 0.059, *r* = 0.32) and Cohen’s *κ* (*p*_*adj*_ = 0.050, *r* = 0.34). As observed for the healthy cohort, SOMNUS clearly outperforms SimpleSleepNet in the classification of Wake (*p*_*adj*_ = 0.026, *r* = 0.37) and REM sleep (*p*_*adj*_ < 0.001, *r* = 0.62).

#### Alignment with human consensus

To assess whether SOMNUS tends to replicate a consensus of human scorers rather than the style of individual experts, we evaluate the inter-scorer agreement using Cohen’s *κ*, comparing the model’s predictions both to individual annotations and to the consensus labels. Additionally, we quantify the similarity between SOMNUS predicted hypnodensity graphs and those computed by the soft-consensus using the ACS metric^12^. In Table 6 we report both the Cohen’s *κ* agreement scores and the ACS values for DOD-H and DOD-O datasets. In both cases, the agreement with the consensus is significantly higher than with any individual scorer (*p*_*adj*_ < 0.001), suggesting that SOMNUS predictions are more closely aligned with collective scoring patterns. Moreover, the high mean ACS values observed, where a value of 1 would imply perfect agreement, indicate that SOMNUS not only matches the consensus labels, but also closely captures the underlying probability distributions reflecting scorer uncertainty.

**Table 6:** Agreement metrics between SOMNUS and human annotations

Dataset	Expert 1	Expert 2	Expert 3	Expert 4	Expert 5	Consensus	ACS
DOD-H	78.8 ± 17.0	81.7 ± 6.1	81.0 ± 10.2	74.7 ± 12.1	82.1 ± 8.7	88.9 ± 4.7	0.947 ± 0.021
DOD-O	74.9 ± 13.6	77.5 ± 10.3	73.0 ± 12.7	78.9 ± 9.7	79.6 ± 9.6	85.4 ± 8.0	0.939 ± 0.024

Agreement of SOMNUS with individual human scorers, their consensus (Cohen’s *κ*, mean ± std), and soft-consensus, quantified using Averaged Cosine Similarity (ACS), on the DOD-O and DOD-H datasets.

Bold values for each table now explained in corresponding caption.

We further investigate whether the observed alignment with consensus was specific to SOMNUS or extended to other model configurations. In Supplementary Table 21 we report Cohen’s *κ* and ACS scores between various ensemble models, individual models, and the human consensus across DOD-H and DOD-O datasets. The consensus-oriented behavior is consistent across all models. Nonetheless, combining predictions across different architectures and channel configurations, as done in SOMNUS, yields additional gains, producing the highest levels of consensus alignment across both datasets. We observe that the inter-model soft-agreement^19^ within SLEEPYLAND exceeds the soft-agreement observed among human experts. On DOD-H, the average soft-agreement across human scorers is 0.894 ± 0.028, compared to 0.944 ± 0.019 among the models. Similarly, on DOD-O, models show slightly higher agreement (0.902 ± 0.081) compared to human scorers (0.885 ± 0.014).

#### Ensemble disagreement as an indicator of scoring ambiguity

Building on the observation that SLEEPYLAND models align more closely with the consensus than with individual scorers, we further examine whether model-ensemble variability can serve as a proxy for highlighting regions of ambiguity in sleep staging. This is particularly important to promote human-in-the-loop workflows, where flagged uncertain segments—identified by the model—can be selectively reviewed and corrected by clinicians, ultimately improving scoring accuracy and clinical reliability. We characterize ensemble disagreement exploiting two complementary metrics: the *entropy* of the soft-voting ensemble output; and the *pairwise cosine distance* between predictions of individual models. The *entropy* and the *pairwise cosine distance* are computed against the ground-truth soft-consensus (i.e., level of agreement among human scorers at each epoch). This analysis explores to what extent variability within the ensemble corresponds to regions of human uncertainty and whether inter-scorer disagreement can be predicted from model-derived indicators.

**Fig. 3:**
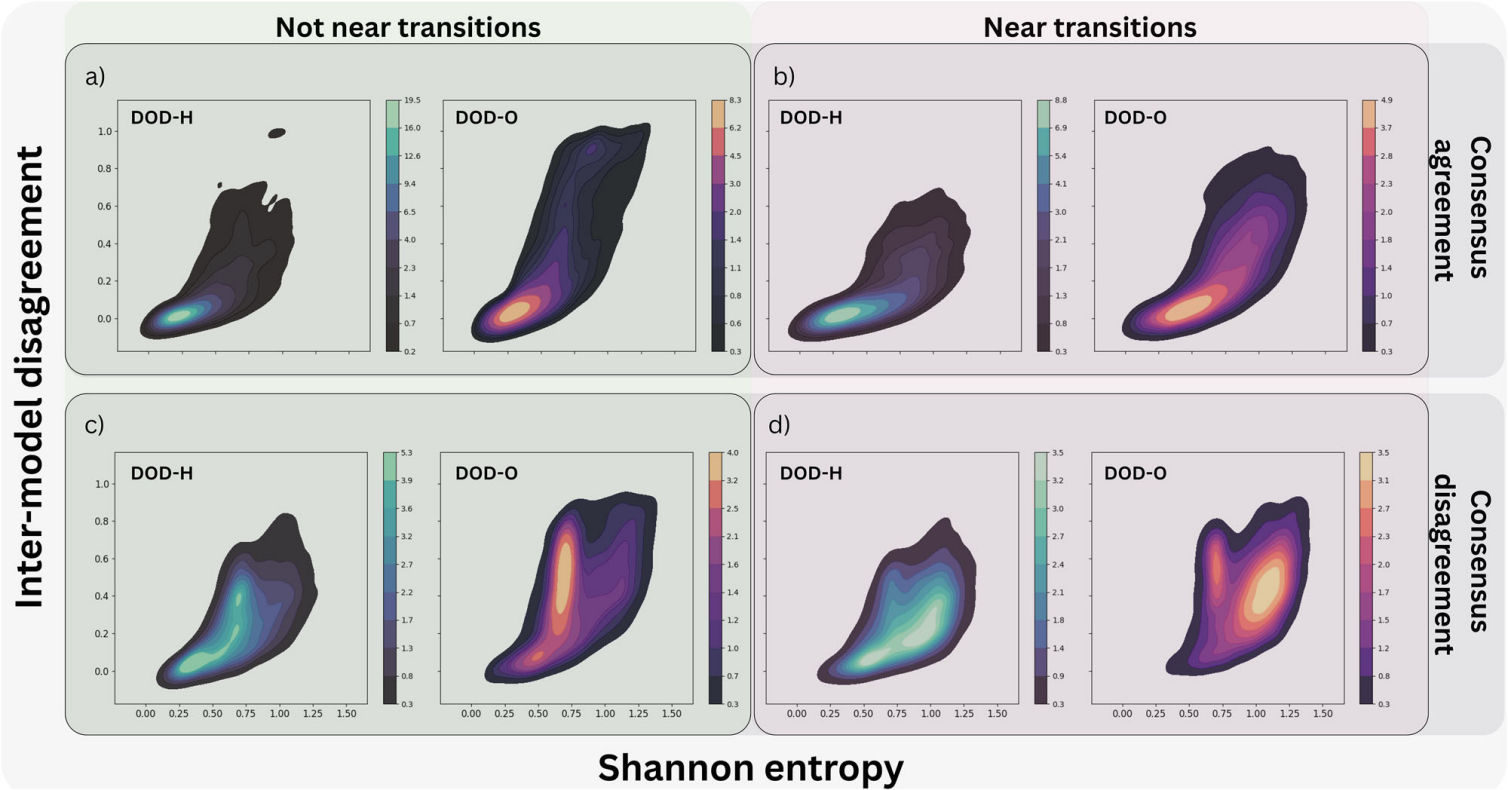
Joint distributions of ensemble entropy and inter-model disagreement. Kernel density estimates illustrate the relationship between Shannon entropy and inter-model disagreement across sleep epochs, stratified by consensus agreement and proximity to sleep stage transitions (**a**–**d**). Columns represent proximity to transitions, and rows correspond to consensus conditions (full agreement vs. disagreement). Panels display distributions separately: **a** not near transitions and consensus agreement; **b** near transitions and consensus agreement; **c** not near transitions and consensus disagreement; **d** near transitions and consensus disagreement—with subplots DOD-H (left) and DOD-O (right). The contours are drawn at iso-proportions of the density, meaning that each curve corresponds to a level set such that a proportion p of the density lies below it. We used 10 levels and set the threshold parameter to 0.05. Inter-model disagreement is represented by the first principal component derived from summary statistics (mean, standard deviation, maximum) of pairwise cosine distances among ensemble constituents.

Fig. 3 shows the joint distribution of ensemble *Shannon entropy* and *inter-model disagreement*, stratified by ground-truth consensus agreement (full agreement vs. disagreement) and proximity to sleep stage transitions—defined as either within one minute of a transition (*near*) or outside that window (*not near*)—based on the consensus hypnogram. We observe a substantial increase in both ensemble entropy and inter-model disagreement in epochs where the soft-consensus reveals disagreement among human scorers. This trend holds across both DOD-H and DOD-O datasets. In regions distant from sleep stage transitions, consensus disagreement is associated primarily with increased inter-model disagreement, particularly in DOD-O where inter-model soft-agreement is lower and performances are more variable. In contrast, entropy shows a more marked increase near transitions, reflecting the intrinsic ambiguity of these periods. Importantly, elevated inter-model disagreement persists even during transitions, suggesting that while entropy is especially sensitive to local uncertainty at boundaries, disagreement among components of the ensemble also captures broader ambiguities beyond stage shifts.

We tested whether ensemble entropy and inter-model disagreement could predict human consensus disagreement, finding that exploiting both led to improved performance. *Simple logistic regression models using features from both sources of variability yielded the highest performance, with mean ROC AUCs of 82.3% on DOD-H and 82.8% on DOD-O, significantly outperforming counterparts using entropy alone (DOD-H: 81.4%, DOD-O: 75.1%) or features derived from pairwise cosine distance alone (DOD-H: 80.8%, DOD-O: 81.6%)*.

## Discussions

SLEEPYLAND is the first tool to fairly evaluate automatic sleep staging models by exploiting an unprecedented scale of harmonized, diverse datasets, including both in-domain and out-of-domain populations. SLEEPYLAND represents the first open-source platform providing sleep staging models pre-trained on ~ 220,000 h of harmonized PSG data from diverse populations covering 24 distinct clinical cohorts. Our framework is unique for two primary research-related reasons and three additional strengths that are crucial in clinical practice. First, the system exploits a standardized evaluation protocol across different architectures and signal configurations, eliminating dataset selection bias prevalent in prior studies. Second, we release the model weights—pre-trained on the largest amount of data to date—openly, which lets researchers quickly fine-tune the network for specific, underrepresented groups (such as children with narcolepsy or older adults with mild sleep apnea) without the expense of starting training from scratch. Third, the system can run directly in a sleep lab, each clinic can test it on its own patients while keeping all data on-site and private. Privacy of sensitive sleep recordings is implicit, since data remain locally in the intranet of the clinics or research institute. Four, predictions of individual PSG recordings can be performed “on-the-fly” in the morning after the data collection, and transferred back to the local clinical or research tool for re-scoring within few minutes. Technicians with no advanced informatics or programming skills can perform all these steps. Five, the sleep staging (hypnogram), together with the epoch-wise scoring certainty score (hypnodensity graph plus, e.g., entropy-derived confidence) is provided to the physicians.

Overall, we show that deep learning models, when trained on such heterogeneous data, can generalize robustly across a wide spectrum of clinical populations and recording protocols. The models leverage any available EEG and/or EOG derivation, offering flexibility in deployment. While this study deliberately focused on conventional AASM-derivations to ensure a controlled comparison across models, our previous work^11^ has demonstrated that unconventional derivations can be equally informative for a deep learning model—i.e., we can still encode useful representations and patterns. The strong performance observed here with single-channel models is not restricted to PSG lab-based montages. In principle, retraining and expanding SOMNUS with unconventional derivations could substantially increase its applicability—reaching higher performances—to existing wearable technologies, such as headbands or in-ear EEG devices, which often provide limited or non-standard signals. Consistent with prior work, majority voting across available EEG and/or EOG derivations reliably outperforms individual channel configurations, and often surpasses the best-performing single-channel setup. Theoretically there is no need for an exhaustive channel selection. The SOMNUS ensemble, which combines predictions across both architectures and channel configurations via soft-voting, consistently exceeds previously reported state-of-the-art performance across a wide range of datasets and evaluation scenarios. SOMNUS demonstrate robust generalization capabilities in both in-domain and out-of-domain contexts. It also surpasses all previously reported state-of-the-art results on the out-of-domain multi-scored Dreem Open Datasets, consistently outperforming (without ever having seen the data before) both all individual human experts, and the originally proposed model^19^ with the data in-training. Our ensemble model not only aligns more closely with the consensus, but also exhibits greater consistency, with significantly lower variability in per-recording performance. SOMNUS outperforms individual models across datasets in nearly 95% of cases, and it is never significantly inferior, highlighting the value of ensemble approaches for robust, plug-and-play deployment in diverse settings. Our benchmarking shows that while certain individual models may outperform others on specific recordings/datasets, ensembling consistently output more reliable results across all the recordings/datasets. *Unless there is clear evidence that a particular model is exceptionally well-suited to a specific dataset, ensemble methods remain the most dependable option for maintaining high and consistent performance across a wide range of evaluation scenarios*. Furthermore, exploiting ensembling approaches allows to complement uncertainty quantification methods based on softmax-derived metrics, by incorporating the available inter-model disagreement between ensemble components. We also observe that the proposed mean recording-level disagreement metrics are all significantly higher in a sleep-disordered cohort compared to an healthy one, suggesting a potential association between model uncertainty and the presence of sleep disorders. This observation highlights that representations derived from SLEEPYLAND models could offer informative cues for a variety of sleep-related downstream applications. While we acknowledge that more sophisticated ensembling strategies, such as stacked ensembles or meta-learners, might yield even better performance, and that more precise uncertainty quantification might be achievable through dedicated auxiliary confidence networks^28^, our focus in this work is on promoting readily available ensembling techniques within SLEEPYLAND. The simplicity of the SOMNUS approach requires no additional training or architecture-specific tuning, making it particularly well-suited for an open-access platform. In this way users can easily define custom ensembles across different subsets of models.

*SLEEPYLAND emphasizes that advances in automated sleep staging are driven more by expanding and diversifying the training data than by introducing new model architectures*. Exposure to such heterogeneous data and diverse scorer styles during training inherently promotes consensus-level generalization and mitigates scorer-specific biases, even without explicitly incorporating multi-annotated data. Models within SLEEPYLAND surpass previous methods that directly leverage multiple annotations during training^12,19^, even achieving superior alignment with soft-consensus. This underscores that large-scale, heterogeneous training alone can be sufficient to learn the generalizable representations necessary to match expert-level consensus. As more PSG data from varied populations and recording settings become publicly available, such as through continued expansion of NSRR, we anticipate further improvements in generalizability. Moreover, while single-dataset experiments can provide useful proof-of-concept validations for model designs, robust claims of generalization demand comprehensive evaluation across multiple, diverse datasets. Moving forward, *adopting multi-dataset training and rigorous in-domain and out-of-domain evaluation should become the standard practice to advance the field and ensure models are truly robust and clinically applicable*.

Considerable variability in macro-F1 scores persists across datasets, driven largely by specific sleep phases, e.g., N1 and N3 stage performance. Datasets with more fragmented or atypical sleep—such as those with narcolepsy (e.g., MNC-SSC data), or multiple comorbidities—remain particularly challenging. Our analysis reveals that automated sleep staging inherits biases rooted in both physiological variability and data limitations. While ensemble approaches like SOMNUS improve robustness, they fail to resolve fundamental disparities linked to demographic and clinical factors. Here, four key insights emerge: (1) Performance degrades non-linearly at pediatric and elderly extremes, driven by AASM scoring rule differences between age groups and age-related EEG/EOG signal changes. For pediatric populations, mismatches arise from distinct sleep patterns criteria, while elderly declines reflect unmodeled comorbidities beyond AHI/PLMI. Our bias-findings contrast with our earlier experiments^11^—where the sub-optimal inclusion of age seemed to offer no benefit, and we therefore did not quantify, e.g., the underlying age-related bias. (2) Apparent gender differences in N3/REM accuracy largely derive from clinical confounders—males in the cohort were older and had higher AHI/PLMI. When controlling for these factors, gender effects diminished but persisted in N3, suggesting residual biological or scoring biases. (3) High AHI/PLMI increased N2/N3/REM variability due to sleep fragmentation. Models partially “corrected" N3 underestimation in severe OSA populations—a consequence of training data oversampling disordered sleep. This may explain how data imbalances can create misleading compensatory behaviors. (4) No model architecture consistently outperformed others in bias mitigation. Ensembling reduced performance gaps but preserved systemic biases, confirming that architectural innovations alone cannot resolve data-driven disparities. A further source of potential bias may arise from the coexistence of different sleep scoring standards across datasets. While most modern recordings follow AASM rules, several of the older datasets were originally scored using R&K criteria. Biases arising from older guidelines may be also linked with scorer expertise and laboratory different practices. Frameworks such as GAMLSS could in principle help assess the role of scoring rules as a contributor to systematic bias. Exploring such multilevel effects represents a promising direction for future experiments, especially if expanded datasets include richer annotations of scorer expertise and/or confidence levels on the scored sleep stages.

Perhaps, the shortening of scoring periods from 30 seconds to, e.g., 5 seconds might better account for and reflect overall sleep physiology^29^. The AASM sleep staging rules have certain limitations to reflect sleep physiology, especially in persons with altered sleep as compared to healthy young adults, like children, elderly and sleep-disordered people. With automatic sleep staging, we have the opportunity to soften the strict historic rules for manual sleep staging, accounting better for actual sleep physiology. In parallel, a recent work has also highlighted the potential of self-supervised approaches for learning general-purpose sleep representations directly from large-scale raw data^25^. While their performance on sleep staging remains slightly below that of supervised models like SOMNUS, their independence from scorer-derived labels offers two key advantages: the ability to sidestep scorer-specific biases and enhanced generalization to downstream clinical prediction tasks. Together, these findings suggest that self-supervised learning represents an important frontier for future development in the field.

Looking ahead, several recommendations emerge:

1. Leverage *deep learning model-ensemble*, exploiting their higher performance, model-disagreement derived metrics to flag ambiguous epochs for expert review.
2. Develop *bias-aware training paradigms*, including explicit conditioning/weighting on demographic and clinical variables and/or underrepresented groups.
3. Expand *data diversity*, prioritizing massively expanded datasets with intentional diversity sampling.
4. Develop *hybrid training strategies*, combining large-scale pre-training on heterogeneous datasets with targeted fine-tuning for specific subgroups or scorer styles.
5. Integrate *human-in-the-loop workflows*, allowing clinicians to review and correct automated outputs, with the potential for model personalization, while carefully managing the risk of overfitting to scorer-specific biases.
6. Establish *standardized reporting*, i.e, noting prediction-derived clinical markers [e.g., SE] along with a flag [high AHI bias], ensuring transparency of model performance across subpopulations.

As the field moves toward broader clinical adoption, a central technical question remains: *will the combination of large-scale, diverse data, open-source tools, and adaptive bias-aware workflows be sufficient to achieve equitable and high-performance sleep staging in all patient populations?*

Is it then possible to start using these models in clinical routine? Obviously, not yet. As a next step, tools like SLEEPYLAND will need to be further integrated within PSG software platforms commonly used in clinical and research institutions. Certification through programs such as the 2024 AASM Autoscoring Certification Pilot Program, as well as regulatory approval as a biomedical product under the EU AI Act, will be essential to transition automatic scoring tools from research into clinical practice. Continued collaboration between researchers, clinicians, and regulatory bodies will be crucial to ensure robust validation, user trust, and safe deployment in real-world healthcare environments.

## Methods

SLEEPYLAND key features are the following: *fair training and benchmarking*, i.e., the DL models integrated within the tool are trained and validated on the same set of harmonized data from the NSRR repository; *modular architecture*, i.e, the modular design allows for flexibility and scalability through containerized services; *local privacy* for user data, i.e., recognizing the sensitive nature of medical/sleep data, the tool ensures that all analysis (e.g., evaluating the models on their own data) is performed locally on the user’s device.

### The architecture

**Fig. 4:**
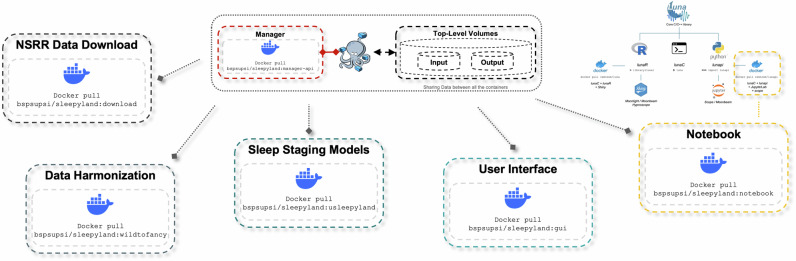
The SLEEPYLAND architecture. Overview of the modular architecture composed of multiple containerized services. The *manager-api* service coordinate communication across components. The *gui* provides a web interface for data management and visualization. The *nsrr-download* service automates data retrieval from NSRR repositories. The *wild-to-fancy* service standardizes inputs by converting and harmonizing sleep recordings and annotations. Sleep staging is performed by the *usleepyland* service, which integrates multiple ML/DL models. A dedicated Jupyter *notebook* container supports advanced and customizable analyses.

SLEEPYLAND has been developed using Docker Compose, with each key service running in a dedicated container. All Docker images and containers are stored in https://hub.docker.com/r/bspsupsi/sleepyland. By following the instructions in the available https://github.com/biomedical-signal-processingrepository, the users can easily execute the tool on Linux, Mac, or Windows, automatically setting up all components in the background. The SLEEPYLAND architecture integrates multiple containerized services, each designed to perform a specific function within a modular and scalable framework (see Fig. 4).

SLEEPYLAND is composed of multiple containerized services:

- The *manager-api* service: a central coordinating service that manages communication between all the implemented components.
- The *gui* service, implemented using Flask, provides an intuitive web-based interface for data upload/download, task execution, and interactive visualization of results.
- To facilitate the use of datasets from the NSRR repository mentioned in Table 1, the *nsrr-download* service automates the retrieval of sleep data and annotations, allowing users to select specific cohorts and subjects.
- Data harmonization is handled by the *wild-to-fancy* service, which converts sleep recordings from .edf to .h5, resamples data, and standardizes annotations across a range of formats.
- Sleep staging is performed by the *usleepyland* service, which integrates state-of-the-art ML and DL models, including YASA^23^, U-Sleep, DeepResNet, and SleepTransformer.
- A Jupyter *notebook* container is included for users who require customizable analyses or the ability to generate detailed reports.

SLEEPYLAND utilizes Docker volumes and mounted directories. Raw input data is stored in a shared input volume accessible to all services, while processed outputs are saved in an output volume. A dedicated directory is mounted for the Jupyter notebook container, allowing users to save and to generate custom analyses and reports. The choice of Docker Compose was driven by several key advantages. First, the use of containerization ensures isolation and modularity, allowing each service to operate independently. This modularity is particularly beneficial in contexts where components require different dependencies or need to be updated without affecting the overall system. Second, Docker Compose simplifies the deployment process by enabling the entire environment to be launched with a single command, reducing the complexity of managing multiple services. Third, Docker provides consistency across computing environments, eliminating potential discrepancies arising from differences in machine configurations or operating systems. It ensures cross-platform compatibility, making SLEEPYLAND accessible to a wide range of users.

### Sleep staging algorithms

The primary goal of SLEEPYLAND is to consolidate the top-performing sleep staging algorithms, trained and evaluated on a wide variety of datasets, including tens of thousands of PSG recordings from the publicly available NSRR data repository^13^, into a single open-source repository. To ensure a fair evaluation framework, a standardized approach is followed for data harmonization and the training procedures, including consistent splits into training, validation, and test sets across all models. In the following sections, we focus primarily on the core *usleepyland* service, which integrates well known high-performing sleep staging models. Below we provide deeper insights into the deep learning architectures exploited, the training procedures, and the data management/sampling strategies.

### Deep learning models

SLEEPYLAND includes a diverse selection of deep learning models for sleep staging, selected for their reported performance, robustness, architectural diversity—including convolutional, recurrent, and attention-based frameworks—and their ability to process different input representations, such as raw signals or spectrograms. Each model’s architecture and hyperparameters closely adhere to the original implementations, with only minimal modifications introduced to ensure compatibility with the proposed training procedure and to facilitate fair comparisons across architectures. These adjustments are strictly limited to aspects required for consistency, compatibility or numerical stability, without additional hyperparameter tuning. Moreover, while the original implementations were often tailored to specific signal channels or fixed derivations, SLEEPYLAND supports both single-channel and multi-channel configurations that can operate on any available derivation, enabling researchers to choose models that best align with their data and needs.

- *U-Sleep*^9^. The model is a purely feed-forward convolutional neural network developed for sleep staging, originally inspired by the U-Net architecture^30,31^. The architecture comprises three main sub-modules. An *encoder module*, composed of sequential blocks that alternate between convolution, ELU activation function, batch normalization, and max pooling. It is designed to extract abstract feature maps from the input signals, progressively reducing their temporal resolution to capture high-level features. The encoder is followed by a *decoder module*, which mirrors the structure of the encoder and gradually reconstructs the temporal resolution of the encoded features, producing a high-frequency sleep stage representation. Skip connections between corresponding encoder and decoder layers help preserve crucial temporal details. Finally, a *segment classifier* processes the high-frequency, intermediate representations generated by the decoder and maps them to a probability distribution across sleep stages, providing the final sleep stage predictions at a desired *τ* seconds interval. In all our experiments, U-Sleep is trained with hyperparameters in line with the original implementation, with the exception of an increased initial learning rate *η* = 10^−5^, adjusted from the original *η* = 10^−7^. We set *τ* = 30 during both training and evaluation.
- *DeepResNet*^10^. The model is based on an adaptation of a ResNet architecture^32^, designed for processing one-dimensional, time-dependent PSG data. The network is organized into four modules. The *mixing module* performs non-linear channel mixing, followed by a *feature extraction module*, composed of a sequence of convolutional residual blocks, capturing key features at intra-epoch level from the signal. The *temporal processing module* then leverages bidirectional GRUs to capture temporal dependencies, allowing the model to consider both preceding and following context for each time segment. Finally, a *classification module* computes the probability distribution for each epoch across sleep stage classes. Notably, in model instances designed for single-channel input, the mixing module is omitted. All SLEEPYLAND versions of DeepResNet closely follow the original architecture, with key adjustments limited to increasing learning rate scheduler patience from 5 to 50 epochs and lowering the initial learning rate to *η* = 10^−5^.
- *SleepTransformer*^16^. The model is a fully transformer-based architecture^33^ tailored for sequence-to-sequence sleep staging. The architecture comprises two main attention-based components. The *epoch transformer*, consisting of four encoder blocks, processes each 30-second sleep epoch by treating it as a sequence of spectral columns, capturing intra-epoch relationships. An additional attention mechanism at the end of this module produces a compact feature vector that encapsulates essential information from each epoch. Following this, the *sequence transformer*, composed of four additional transformer encoder blocks, models inter-epoch dependencies by processing the sequence of feature vectors derived from consecutive epochs. A standard fully connected classification module finally provides probabilistic sleep stage predictions across the sequence. Only minimal modifications were made to adapt SleepTransformer to the proposed framework and improve numerical stability. In both the epoch and sequence transformers *d*_*model*_ was adjusted to align with the number of channels processed in SLEEPYLAND; specifically, it was set to the product of the number of frequency bins and the number of input channels, allowing adaptability to various channel configurations. We also applied standard numerical stabilization strategies, including softmax output clipping, gradient clipping, and increasing the *ϵ* parameter of layer normalization to 10^−6^.

#### Training procedure and data sampling

The training procedure and the data sampling from multiple datasets draws extensively from the original approach proposed in ref.^9^. The ID datasets were divided into training (at least 75%), validation (up to 10%, capped at 50 subjects) and testing (up to 15%, capped at 100 subjects) subsets, with splits performed on a per-subject or per-family basis. The exact number of recordings allocated to each split for every ID dataset is provided in Supplementary Table 2. Notably, for some datasets, multiple PSG recordings were available for the same subject; therefore, the total number of recordings within a split may exceed the maximum number of unique subjects. A *training “epoch"* is defined differently from the conventional deep learning usage; in this context, it refers to the model processing 10^6^ sleep segments. Training is conducted for a maximum of 10000 epochs, with early stopping triggered by a patience of 200 epochs based on the mean validation F1 score across sleep stage classes. In practice, early stopping consistently occurred before reaching the maximum epoch limit. A training batch size of 64 is maintained across all models, with each batch generated as follows: *dataset sampling*, a training dataset is sampled based on a 50/50 probability split between discrete uniform sampling and size-based selection; *subject/recording and channel sampling*, from the chosen dataset, a PSG record is randomly selected, with channel(s) picked uniformly according to availability and model specifications; *segment sampling*, a sleep stage class is then sampled, and a corresponding sleep segment/epoch is chosen randomly within the PSG record. Finally, a segment of the desired length is created to include the selected epoch in a random position. The segment length is fixed at 35 sleep epochs (17.5 minutes of PSG data) for all models. When the model requires time-frequency images instead of raw signals, spectrograms are generated “on-the-fly" following the procedure described in Phan et al.^16^. Each channel undergoes robust scaling at the individual instance level within the batch. Data augmentation is applied for both raw and spectrogram inputs, with a 0.1 probability of replacing parts of sequences with random noise or dropping channels, except for single-channel models where channel dropout is omitted. All models were trained to minimize cross-entropy loss between predicted sleep stages and ground-truth labels. Model definition and training were carried out within a unified TensorFlow 2 framework, with all training conducted on a single NVIDIA L40S GPU. For more details refer to the *usleepyland* service repository https://github.com/biomedical-signal-processing/uSLEEPYLAND.

#### Inference procedure and evaluation

Test-time inference in SLEEPYLAND partially follows the approach outlined in^9^, with adaptations to accommodate architectural differences across the available models. All models predict sleep stages while excluding sleep epochs labeled with annotations unrelated to sleep stages or left unscored by human annotators. Predictions can be generated using all available EEG and/or EOG channel combinations and a majority voting scheme is applied to fuse predictions across channels. Evaluation is conducted comprehensively, assessing both overall performance and class-specific metrics. Global performance is measured using Accuracy, Macro F1, and Cohen’s Kappa^34^, while per-class F1 scores are computed for each of the five sleep stages. Due to architectural differences, inference procedures vary across models. U-Sleep, being fully convolutional, allows for one-shot evaluation, processing an entire PSG in a single forward pass, as in the original implementation. In contrast, DeepResNet, which includes recurrent components, and SleepTransformer, which relies on positional encoding, do not support this approach. Empirical results indicate that evaluating these models on extended sequences in a single forward pass leads to consistent performance degradation. Therefore, both DeepResNet and SleepTransformer are evaluated on fixed-length segments of 35 sleep epochs, mirroring their training setup. These segments are processed as independent instances within a batch, and the final sleep stage predictions are obtained by concatenating the outputs across segments. While overlapping window evaluation could theoretically be applied to refine predictions, the associated computational cost significantly outweighs the minor performance gains observed and is therefore not implemented in SLEEPYLAND.

#### Model-ensembling

To combine predictions from multiple automatic sleep staging algorithms within SLEEPYLAND, we adopt a soft-voting ensemble strategy. In this approach, each model *m* ∈ {1, …, *M*} produces a probability distribution ${\widehat{y}}_{m}^{t}\in {{\mathbb{R}}}^{5}$ over the five sleep stages for each sleep epoch *t*. The ensemble output is computed as the element-wise average across all constituent model predictions:

1 $${\widehat{y}}_{E}^{t}=\frac{1}{M}\mathop{\sum }\limits_{m=1}^{M}{\widehat{y}}_{m}^{t}$$

We refer to such ensembles as SOMNUS models, constructed by aggregating predictions across different subsets of network architectures and channel configurations available in SLEEPYLAND. These configurations may be restricted to a fixed model architecture or signal modality, or may span all available models in a heterogeneous ensemble. We choose soft-voting as the default ensembling strategy in SLEEPYLAND due to its flexibility and compatibility with the toolbox’s modular design. Provided that a model produces probabilistic output at the sleep-epoch level, it can be seamlessly integrated into a soft-voting ensemble.

### Bias on performance and clinical markers

To evaluate robustness of different sleep staging approaches, we adopted the GAMLSS framework^20,21,35^, which models the distribution of performance metrics and prediction-derived clinical markers as a function of external covariates *X*. This approach captures systematic shifts in algorithmic behavior—referred to as *biases*—and extends standard validation techniques (e.g., correlations with clinical variables, Bland-Altman plots). By enabling the estimation of arbitrary bias and performance quantiles conditional on subjects’ characteristics *X*, it provides detailed insights essential for the clinical adoption of sleep-scoring algorithms. Recording-level performance metrics, including the macro F1-score (MF1) and stage-specific F1-scores (F1_W_, F1_N1_, F1_N2_, F1_N3_, F1_REM_), bounded in [0, 1], were modeled using a zeros-and-ones-inflated Beta distribution^36^. The location (*μ*) and scale (*σ*) distributional parameters were modeled with a logit link, and the zero- and one-inflation parameters (*ν*, *τ*)—capturing completely incorrect (0%) and perfect (100%) sleep-scoring predictions—with a log link. Due to the limited number of cases with perfect or completely incorrect hypnogram predictions (e.g., ≤4 cases for MF1 across all models), covariate effects (cubic spline for age; linear terms for AHI, PLMI, and gender) were modeled only for the location (*μ*; expectation) and scale (*σ*; variability) distributional parameters. The parameters (*ν*, *τ*) of the inflated Beta distributions were treated as constants using intercept only. Prediction-derived -clinical markers were quantified by modeling the differences $\widehat{y}-{y}_{{\rm{ref.}}}$ between predicted and (expert-scored) reference hypnogram-derived sleep markers using a generalized Normal distribution, with *μ* and *σ* modeled by identity and log links, respectively. The markers considered included total sleep time (TST), wake after sleep onset (WASO), stage-specific sleep durations (N1, N2, N3, REM) [minutes], and REM latency (REML) [minutes], and the rates of awakenings (AwH) and transitions (TrH) per hour [N/hour]. Potential *bias-inducing covariates* (*X*) included age (in years above 50), gender (binary, female as reference), apnea-hypopnea index (AHI, divided by 10), and periodic limb movement index (PLMI, divided by 10). The effect of age was modeled using a penalized cubic spline to capture known non-linear associations^20^, while AHI and PLMI were modeled linearly, assuming proportional effects with event frequency. Gender was included as a binary covariate with female as the baseline. The optimal set of predictors for each distributional parameter was determined using forward stepwise regression based on the generalized Akaike information criterion (GAIC)^35^.

In Supplementary Notes: Calculation of expected values from GAMLSS, we report concrete examples of converting the table’s estimates into expected values. For derivation of conditional variance and quantiles, see ref. ^35^.

### Evaluation protocol for multi-annotated datasets

To investigate the well known challenge of inter-scorer variability in sleep staging^26^ and evaluate the robustness of SLEEPYLAND models with respect to it, we adopt the multi-annotator evaluation framework and the Dreem Open Datasets introduced by Guillot et al.^19^, allowing for comparison of automatic models against annotations from five expert scorers.

To assess statistical significance of median performance differences and consistency, quantified as the absolute deviation from the median Cohen’s *κ*, across recordings, we conduct one-sided Wilcoxon signed-rank tests with Bonferroni-Holm correction for multiple comparisons. Effect size are reported as $r=\frac{Z}{\sqrt{N}}$. Comparisons against human experts are made relative to the best-performing individual scorer for each metric, providing the most challenging baseline.

Given *S* human scorers, let ${y}_{s}^{t}\in \{0,1,2,3,4\}$ denote the label assigned by scorer *s* to epoch at time *t* ∈ {1, …, *T*}, corresponding respectively to the five sleep stages Wake, N1, N2, N3, and REM. Let ${\widehat{y}}_{s}^{t}\in {\{0,1\}}^{5}$ be the one-hot encoding of ${y}_{s}^{t}$. The probabilistic consensus for scorer *s* at epoch *t*, denoted ${\widehat{z}}_{s}^{t}\in {[0,1]}^{5}$, is defined as:

2 $${\widehat{z}}_{s}^{t}=\frac{\sum\nolimits_{i=1\,i\ne s}^{S}{\widehat{y}}_{i}^{t}}{\max\left(\sum\nolimits_{i=1\,i\ne s}^{S} {{{\widehat{y}}_{i}^{t}}}\right)}$$

This assigns each sleep stage a score proportional to the number of other scorers who selected it, normalized such that the most frequent stage(s) receive a value of 1. In the case of ties, multiple stages may share the maximum value. The *soft-agreement* ∈ [0, 1] of scorer *s* over a recording is then computed as:

3 $${{\rm{Soft-Agreement}}}_{s}=\frac{1}{T}\displaystyle \mathop{\sum }\limits_{t=1}^{T}{\hat{z}}_{s}^{t}[{y}_{s}^{t}]$$

This metric quantifies the extent to which a scorer’s annotations align with the collective judgment of the other scorers, weighted by the level of inter-scorer agreement at each timepoint. A value of 1 indicates perfect alignment with the majority at all epochs, while 0 indicates complete disagreement. Soft-agreement is computed for all human scorers across all recordings in the multi-scored datasets. The same formulation is also used to quantify agreement among SLEEPYLAND models, treating each model as an independent scorer. The *consensus hypnogram* for a recording is obtained via majority voting across scorers at each epoch, with ties resolved using the decision of the most reliable scorer, defined as the scorer with the highest soft-agreement for that recording. For human annotators, the consensus hypnogram is computed excluding the scorer being evaluated and is derived from the remaining four scorers. For automated methods, the consensus is computed from the set ${\mathfrak{C}}$ consisting of the four most reliable human scorers across the dataset.

In addition to the discrete consensus hypnogram, we compute a *soft-consensus* vector ${\widehat{y}}_{{\mathfrak{C}}}^{t}$ at each epoch *t*, as introduced in ref.^12^, defined as:

4 $${\widehat{y}}_{{\mathfrak{C}}}^{t}=\frac{1}{| {\mathfrak{C}}| }\mathop{\sum }\limits_{s\in {\mathfrak{C}}}{\widehat{y}}_{s}^{t}$$

This represents the empirical distribution of the annotations provided by scorers in ${\mathfrak{C}}$.

The *hypnodensity graph* is a visualization tool that represents the probability distribution over sleep stages for each epoch across time, providing a continuous depiction of uncertainty and transitions in sleep staging^22^. For human annotations, it is constructed from the soft-consensus vectors ${\widehat{y}}_{{\mathfrak{C}}}^{t}$ computed at each epoch *t*, offering a probabilistic summary of inter-scorer agreement over the course of a recording. Similarly, for automatic models, the hypnodensity graph is obtained by stacking the model’s predicted probability distributions ${\widehat{y}}_{m}^{t}$ across epochs. In both cases, hypnodensities capture the temporal dynamics and ambiguity in staging decisions, enabling comparison between models and human annotators beyond discrete labels.

The cosine similarity between two probability distributions over sleep stages, such as the predicted distribution ${\widehat{y}}_{m}^{t}$ from a model and the reference distribution ${\widehat{y}}_{{\mathfrak{C}}}^{t}$ from the soft-consensus, at epoch *t* is defined as:

5 $${\,{\rm{sim}}}_{m,{\mathfrak{C}}}^{t}=\frac{{\widehat{y}}_{m}^{t}\cdot {\widehat{y}}_{{\mathfrak{C}}}^{t}}{\parallel {\widehat{y}}_{m}^{t}\parallel \parallel {\widehat{y}}_{{\mathfrak{C}}}^{t}\parallel }$$

This metric quantifies the angular similarity between two vectors, with values closer to 1 indicating greater alignment. To summarize similarity across two hypnodensity graphs, the Averaged Cosine Similarity (ACS)^12^ is computed as the mean cosine similarity over all *T* epochs:

6 $${{\rm{ACS}}}_{m,{\mathfrak{C}}}=\frac{1}{T}\mathop{\sum }\limits_{t=1}^{T}{\,{\rm{sim}}}_{m,{\mathfrak{C}}}^{t}$$

In our work, ACS provides a single scalar value reflecting how closely a model’s hypnodensity output aligns with the soft-consensus of human scorers over the full recording.

To quantify variability within SOMNUS, we compute two complementary measures: *Shannon entropy* of the ensemble output and inter-model disagreement based on *pairwise cosine distance*. Given a probability distribution $p\in {{\mathbb{R}}}^{C}$ over *C* classes, the Shannon entropy *H* is defined as

7 $$H=-\mathop{\sum }\limits_{c=1}^{C}p[c]\cdot \log \left(p[c]\right)$$

Entropy reaches its maximum when the distribution is uniform and minimum when all probability mass is concentrated on a single class. In our work, we compute the entropy of SOMNUS at each epoch *t*, considering the probability distribution ${\widehat{y}}_{E}^{t}$ over the five sleep stages. Higher entropy indicates greater uncertainty in the ensemble’s output distribution. To characterize inter-model disagreement, we compute the set of pairwise cosine distances between all unique pairs of model predictions at each epoch *t*:

8 $${D}^{t}=\left\{1-{\,{\rm{sim}}}_{m,n}^{t}\,| \,m < n,\,m,n\in \{1,\ldots ,M\}\right\}$$

where ${\,{\rm{sim}}}_{m,n}^{t}$ denotes the cosine similarity between the predicted probability distributions of models *m* and *n*, components of the ensemble.

Human consensus disagreement prediction is framed as a binary classification problem, with the target indicating whether human scorers disagreed at a given epoch. Logistic regression classifiers are trained using features derived from ensemble variability, namely, the Shannon entropy of SOMNUS output and summary statistics (mean, standard deviation, and maximum values at each epoch) of the pairwise cosine distance set *D*^*t*^, capturing different facets of disagreement among ensemble constituents. Performance is evaluated separately on DOD-H and DOD-O using leave-one-recording-out cross-validation.

## Ethical approval

The secondary usage of the BSWR dataset was approved by the local ethics committee (Kantonale Ethikkommission Bern [KEK]-Nr. 2022-00415), ensuring compliance with the Human Research Act (HRA) and Ordinance on Human Research with the Exception of Clinical Trials (HRO). All methods were carried out in accordance with relevant guidelines and regulations. Written informed consent was obtained from all participants as of the introduction of the general consent process at Inselspital in 2015. Data were maintained with confidentiality throughout the study.

## Supplementary information

Supplementary Information

## Data Availability

Due to patient confidentiality and ethical restrictions, the BSWR dataset from Inselspital, University Hospital Bern, is not publicly available. However, de-identified data may be obtained upon reasonable request, subject to a data sharing agreement and approval by the relevant ethics committees. For all other dataset access, kindly refer to the specific access details associated with each of them.

## Code availability

SLEEPYLAND is an open-source toolbox publicly available at https://github.com/biomedical-signal-processing/sleepyland. The complete framework, including the pre-trained models, has been containerized and made accessible via Docker Hub at https://hub.docker.com/r/bspsupsi/sleepyland, ensuring reproducibility and ease of deployment. Additionally, we provide the uSLEEPYLAND repository https://github.com/biomedical-signal-processing/uSLEEPYLAND, which serves as an extension of the U-Time framework^9^. This revised version has been adapted for integration into the SLEEPYLAND project, incorporating additional ML models to enhance its utility in sleep staging analysis. uSLEEPYLAND expands the scope of available methods by exposing feature-based and deep learning models, which can be systematically evaluated within the SLEEPYLAND framework. Detailed installation instructions and a quick start tutorial for SLEEPYLAND are provided through an online video playlist, available at https://www.youtube.com/playlist?list=PLl7J6NiydmVKJ2SAh7fTKaSK-JoPTgvG-.
