# Supplementary material for: SLEEPYLAND: trust begins with fair evaluation of automatic sleep staging models
Source: NPJ Digit Med. 2025 Dec 16;9:55. doi: 10.1038/s41746-025-02237-2 (PMC12816009; doi:10.1038/s41746-025-02237-2)
Supplement: Supplementary file 1 — Supplementary Information [file 41746_2025_2237_MOESM1_ESM.pdf]

SLEEPYLAND: trust begins with fair evaluation of automatic sleep staging models

Alvise Dei Rossi[1,2], Matteo Metaldi[1], Michal Bechny[1,3], Irina Filchenko[4], Julia van der Meer[4], Markus H. Schmidt[4], Claudio L.A. Bassetti[4], Athina Tzovara[3,4], Francesca D. Faraci[1] and Luigi Fiorillo[1,5*]

[1*]Institute of Digital Technologies for Personalized Healthcare | MeDiTech, Department of Innovative Technologies, University of Applied Sciences and Arts of Southern Switzerland, Via la Santa 1, Lugano, 6962, Switzerland.
[2]Faculty of informatics, Università della Svizzera Italiana, Via Giuseppe Buffi 13, Lugano, 6900, Switzerland.
[3]Institute of Informatics, University of Bern, Neubrückstrasse 10, Bern, 3012, Switzerland.
[4]Sleep Wake Epilepsy Center | NeuroTec, Department of Neurology, Inselspital, Bern University Hospital, University of Bern, Freiburgstrasse, Bern, 3010, Switzerland.
[5*]Neurocenter of Southern Switzerland, Ente Ospedaliero Cantonale, Via Tesserete 46, Lugano, 6900, Switzerland.

*Corresponding author(s). E-mail(s): luigi.fiorillo@supsi.ch;
Contributing authors: alvise.dei.rossi@usi.ch; matteo.metaldi@supsi.ch; michal.bechny@supsi.ch; irina.filchenko@insel.ch; julia.vandermeer@insel.ch; markus.schmidt@insel.ch; claudio.bassetti@insel.ch; athina.tzovara@inf.unibe.ch; francesca.faraci@supsi.ch;

Supplementary notes

Dataset

We report a detailed description of all the datasets used in our experiments.

NSRR datasets

ABC. The Apnea, Bariatric surgery, and CPAP database consists of 132 recordings from 49 patients with severe obstructive sleep apnea and morbid obesity (BMI from 35 to 45) [1, 2]. EEG signals (F4-M1, F3-M2, C4-M1, C3-M2, O2-M1, O1-M2) and EOG signals (E2-M1, E1-M2) are considered in our experiments. The signals are recorded at 256Hz, and are hardware low-pass filtered at 105Hz and high-pass filtered at 0.16Hz. The recordings are manually scored by sleep experts according to the AASM rules. For more information we refer to https://doi.org/10.25822/nx52-bc11 and https://clinicaltrials.gov/ct2/show/NCT01187771.

APOE. The Sleep Disordered Breathing, apolipoprotein E (ApoE), and Lipid Metabolism dataset is an NIH-supported study investigating genetic associations in ApoE e4-positive and e4-negative individuals with varying degrees of sleep apnea [1, 3]. We consider 712 recordings from suspected but untreated sleep-disordered breathing participants. EEG signals (C3-M2, C4-M1, O2-M1, O1-M2, C3-M1, C4-M2, O2-M2, O1-M1, F1-M2, F2-C4, F2-T4, FP1-C3, FP1-C3, FP2-C4, Fz-M1, Fz-M2, T3-O1 T4-O2) and EOG signals (ROC-M1, LOC-M2) are included. The signals are recorded at 256Hz using the Sandman Elite system. The recordings are manually scored by sleep experts following the AASM rules. For more information we refer to https://doi.org/10.25822/6ssj-2157.

APPLES. The Apnea Positive Pressure Long-term Efficacy Study (APPLES) is a 6-month, multi-center, randomized, double-blind, sham-controlled trial across five U.S. sites [1, 4]. In our experiments we consider 1094 recordings from 1098 OSA participants. EEG signals (C3-M2, C4-M1, O2-M1, O1-M2) and EOG signals (ROC-M1, LOC-M2) are included. The signals are recorded at 128 Hz. The recordings are manually scored by sleep experts according to the Rechtschaffen and Kales rules, and re-aligned to the AASM rules. For more information we refer to https://doi.org/10.25822/63pr-a591 and https://clinicaltrials.gov/ct2/show/results/NCT00051363.

CCSHS. The Cleveland Childrend's Sleep and Health Study consists of children and adolescents recordings. In our experiments we consider 515 recordings from adolescents aged 16-19 years. A small percentage of the subjects suffers from sleep related movement disorders. The recordings are collected in three different hospitals around Cleveland, Ohio, US [1, 5]. EEG signals (C4-A1, C3-A2) and EOG signals (ROC-A1, LOC-A2) are considered in our experiments. The signals are recorded at 128Hz, and hardware

high-pass filtered at 0.15Hz. The recordings are manually scored by sleep experts according to the AASM rules. For more information we refer to https://doi.org/10.25822/cg2n-4y91.

CFS. The Cleveland Family Study is a family-based study on sleep apnea disordered subjects. The database consists of 2284 subjects from 361 families [1, 6]. We consider recordings of 730 subjects from 144 families (whence full whole-night PSG were available). For this specific database, the data split (train/val/test set) is done by considering subjects and family belonging (*i.e.*, all the family members appear in the same data split). More than half of the subjects are affected by sleep apnea disorder. EEG signals (C4-A1, C3-A2) and EOG signals (ROC-A1, LOC-A2) are considered in our experiments. The signals are recorded at 128Hz, and hardware low-pass filtered and high-pass filtered at 105Hz and 0.16 Hz respectively. The recordings are manually scored by sleep experts according to the AASM rules. For more information we refer to https://doi.org/10.25822/jmyx-mz90.

CHAT. The Childhood Adenotonsillectomy Trial database consists of 1638 recordings (452 baseline, 407 follow-up and 779 control) from 1232 children post-adenotonsillectomy-surgery aged 5-10 years with mild to moderate obstructive sleep apnea. The recordings are collected in six different sleep centers in Massachusetts, Missouri, New York, Ohio and Pennsylvania [1, 7, 8]. EEG signals (F4-M1, F3-M2, C4-M1, C3-M2, O2-M1, O1-M2, T4-M1, T3-M2) and EOG signals (E2-M1, E1-M2) are considered in our experiments. The signals are recorded at 200Hz (or higher in other sleep centers), and different hardware filtering given the different acquisition systems. One recording is excluded - EOG missing. The recordings are manually scored by sleep experts according to the AASM rules. For more information we refer to https://doi.org/10.25822/d68d-8g03 and https://clinicaltrials.gov/ct2/show/NCT00560859.

HOMEPAP. The Home Positive Airway Pressure database consists of 373 recordings (246 considered in our experiments) from obstructive sleep apnea patients aged over 18 years. The recordings are collected in seven different US sleep centers [1, 9]. EEG signals (F4-M1, F3-M2, C4-M1, C3-M2, O2-M1, O1-M2, T4-M1, T3-M2) and EOG signals (E2-M1, E1-M2) are considered in our experiments. The signals are recorded at 200Hz, no filtering applied. Nine recordings are excluded - EOG and/or reference channels missing. The recordings are manually scored by sleep experts according to the AASM rules. For more information we refer to https://doi.org/10.25822/xmwv-yz91 and https://clinicaltrials.gov/ct2/show/NCT00642486.

MESA. The Multi-Ethnic Study of Atherosclerosis consists of 2237 recordings (2056 considered in our experiments) from a cohort of black, white, Hispanic and Chinese-American subjects aged 45-84 years [1, 10]. About

15.0% of individuals have severe SDB, 30.9% short sleep duration, 6.5% poor sleep quality and 13.9% daytime sleepiness. EEG signals (Fz-Cz, C4-M1, Cz-Oz) and EOG signals (E2-Fpz, E1-Fpz) are considered in our experiments. The signals are recorded at 256Hz, and hardware low-pass filtered at 100Hz. The recordings are manually scored by sleep experts according to the AASM rules. For more information we refer to https://doi.org/10.25822/n7hq-c406.

MNC. The Mignot Nature Communications dataset contains raw polysomnography data first exploited in a neural-network–based automated sleep staging project [11]. We consider recordings from approximately 1000 normal and abnormal subjects. EEG signals (C3-M2, C3, C4-M1, C4, Cz, F3-M2, F3, F4-M1, F4, O1-M2, O1, O2-M1, O2) and EOG signals (E1-M2 E1 E2-M1 E2) are recorded at 128Hz. The recordings are manually scored by sleep experts according to the AASM rules. For more information we refer to https://doi.org/10.25822/00tc-zz78.

MROS. The database is a subset of the larger study Osteoporotic Fractures in Men, involving 5994 community-dwelling men aged over 65 years [1, 12, 13]. In our experiments we consider 3930 recordings from subjects which underwent in-home overnight PSG. Most of the subjects are sleep breathing disorders patients. EEG signals (C4-A1, C3-A2) and EOG signals (ROC-A1, LOC-A2) are considered in our experiments. The signals are recorded at 256Hz, and hardware high-pass filtered at 0.15Hz. Seven recordings are excluded - EOG channels and/or sleep stage annotation files missing. The recordings are manually scored by sleep experts according to the AASM rules. For more information we refer to https://doi.org/10.25822/kc27-0425.

MSP. The Maternal Sleep in Pregnancy and the Fetus (MSP) dataset includes 106 overnight laboratory-based PSG recordings from women in their 36th week of pregnancy [1, 14, 15]. Eligibility was restricted to non-smoking women with pre-pregnancy obesity (BMI > 30 kg/m^2), without previously identified sleep disorders or significant conditions compromising mother or fetus (hypertension and diabetes were not exclusion criteria). In our experiments we consider recordings from 105 subjects. EEG signals (C3-M2, C4-M1, F3-M2, F4-M1, O1-M2, O2-M1) and EOG signals (LOC, ROC) are included. Signals are recorded at 256Hz. Recordings are manually scored by sleep experts according to AASM criteria. For more information we refer to https://sleepdata.org/datasets/msp.

NCHSDB. The Nationwide Children's Hospital Sleep DataBank consists of 3984 pediatric sleep studies from 3673 patients aged 0–18 years, collected between 2017–2019 at Nationwide Children's Hospital, Columbus, Ohio [1, 16]. In our experiments we consider recordings from 3950 subjects. EEG signals (FP1, FP2, FZ, CZ, PZ, OZ, FPZ, P3-M2, P4-M1, F3-M2, F4-M1, F4-M2, C3-M2, C4-M1, C4-M2, T3-M2, T4-M1, O1-M2, O2-M1, F4, O1, O2)

and EOG signals (E1-M2, E2-M1, E1, E2) are included. Signals are recorded at 256Hz. Recordings are manually scored by sleep technicians following AASM criteria and include longitudinal clinical data. For more information we refer to https://sleepdata.org/datasets/nchsdb.

SHHS. The Sleep Heart Health Study consists of 8444 recordings (5793 from visit 1 and 2651 from visit 2) from 5797 subjects aged over 40 years [1, 17]. Most of the subjects suffer from OSA or other SDB. EEG signals (C4-A1, C3-A2) and EOG signals (ROC-A1, LOC-A2) are considered in our experiments. The EEG and EOG signals are recorded at 125Hz and 50Hz respectively, and hardware high-pass filtered at 0.15Hz. The recordings are manually scored by sleep experts according to the Rechtschaffen and Kales scoring rules, and re-aligned to the AASM rules. For more information we refer to https://clinicaltrials.gov/ct2/show/NCT00005275 and https://doi.org/10.25822/ghy8-ks59.

SOF. The database is a subset of the larger study Osteoporotic Fractures. In our experiments we consider 453 recordings (from visit 8), which underwent in-home overnight PSG [1, 18, 19]. EEG signals (C4-A1, C3-A2) and EOG signals (ROC-A1, LOC-A2) are considered in our experiments. The EEG and EOG signals are recorded at 128Hz, and hardware high-pass filtered at 0.15Hz. The recordings are manually scored by sleep experts according to the Rechtschaffen and Kales scoring rules, and re-aligned to the AASM rules. For more information we refer to https://doi.org/10.25822/e1cf-rx65.

WSC. The Wisconsin Sleep Cohort is a longitudinal study of 1500 Wisconsin state employees, assessed at four-year intervals, investigating sleep disorders, particularly obstructive sleep apnea [1, 20]. In our experiments we consider 2569 recordings (recordings from visit 1 to visit 4). EEG signals (F3-M1, F3-M2, F3-AVG, F4-M1, F4-M2, F4-AVG, Fz-M1, Fz-M2, Fz-AVG, Cz-M1, Cz-M2, Cz-AVG, C3-M1, C3-M2, C3-AVG, C4-M1, C4-M2, C4-AVG, Pz-M1, Pz-M2, Pz-AVG, Pz-Cz, O1-M1, O1-M2, O1-AVG, O2-M1, O2-M2, O2-AVG) and EOG signals (E1, E2) are included. Signals are recorded at 100Hz-200Hz during in-laboratory PSG. Recordings are manually scored by sleep experts following AASM criteria. For more information we refer to https://sleepdata.org/datasets/wsc.

Out-of-domain datasets

BSWR. The Bern Sleep-Wake Registry consists of 8950 recordings from 7985 subjects. A small percentage of the subjects is healthy (below 1%). The rest of the subjects are patients with a single or multiple sleep disorders or with an uncertain diagnosis. The most common class of sleep disorders is sleep related breathing disorders, followed by central disorders of hypersomnolence, insomnia, parasomnias and sleep related movement disorders. A smaller percentage of patients with circadian rhythm sleep-wake disorders and isolated

symptoms and normal variants is also present. EEG signals (F4-M1, F3-M2, C4-M1, C3-M2, O2-M1, O1-M2) and EOG signals (E2-M1, E1-M2) are considered in our experiments. The signals are recorded at 200Hz. The recordings are manually scored by sleep experts according to the AASM rules. Given the different scoring rules for infants (≤ 2 months) [21], it is important to specify, in the context of the following age analysis, that in the BSWR dataset there were no babies younger than two months.

DCSM. The Danish Centre for Sleep Medicine database consists of 255 recordings from patients with potential and non-specific sleep related disorders [22]. No demographic information is available for the database. EEG signals (F4-M1, F3-M2, C4-M1, C3-M2, O2-M1, O1-M2, T4-M1, T3-M2) and EOG signals (E2-M1, E1-M2) are considered in our experiments. The signals are recorded at 256Hz, and band-pass filtered between 0.3Hz and 70Hz. The recordings are manually scored by sleep experts according to the AASM rules. For more information we refer to https://sid.erda.dk/wsgi-bin/ls.py?share_id=fUH3xbOXv8.

DOD-H & DOD-O. The DOD-H dataset contains 25 recordings (19 males and 6 females) from healthy adult volunteers aged from 18 to 65 years. The recordings were collected at the French Armed Forces Biomedical Research Institute's (IRBA) Fatigue and Vigilance Unit (Bretigny-Sur-Orge, France). EEG signals (C3-M2, C4-M1, F3-F4, F3-M2, F3-O1, F4-O2, O1-M2, O2-M1) and left/right EOG signals are considered. The recordings are sampled at 512 Hz. The DOD-O dataset contains 55 recordings (35 males and 20 females) from patients suffering from obstructive sleep apnea aged from 39 to 62 years. The recordings were collected at the Stanford Sleep Medicine Center. EEG signals (C3-M2, C4-M1, F4-M1, F3-F4, F3-M2, F3-O1, F4-O2, FP1-F3, FP1-M2, FP1-O1, FP2-F4, FP2-M1, FP2-O2) and left/right EOG signlas are considered. The recordings are sampled at 250 Hz. As in [23], a band-pass Butterworth IIR filter is applied between [0.4, 18] Hz to remove residual PSG noise, and the signals are resampled at 100 Hz. The signals are then clipped and divided by 500 to remove extreme values. The recordings are scored by five physicians from three different sleep centers according to the AASM rules. For more information we refer to [23].

PHYS. The database from the 2018 PhysioNet/CinC Challenge consists of 1985 recordings (994 labelled considered in our experiments) from patients with potential sleep disorders [24, 25]. EEG signals (F4-M1, F3-M2, C4-M1, C3-M2, O2-M1, O1-M2) and one EOG signal (E1-M2) are considered in our experiments. The signals are recorded at 200Hz. The recordings are manually scored by sleep experts according to the AASM rules. For more information we refer to https://physionet.org/content/challenge-2018/1.0.0/.

SEDF-SC & SEDF-ST. The Sleep-EDF Expanded database consists of 197 recordings from two subset studies. The Sleep-EDF Sleep Cassette consists of 153 recordings from 78 healthy subjects aged 25-101 years. The Sleep-EDF Sleep Telemetry consists of 44 recordings from 22 healthy subjects with mild difficulty falling asleep (two recordings collected for each subject, *i.e.*, one after temazepam intake and one after placebo intake) [24, 26]. EEG signals (Fpz-Cz, Pz-Oz) and one EOG signal (ROC-LOC) are considered in our experiments. The signals are recorded at 100Hz. The recordings are manually scored by sleep experts according to the Rechtschaffen and Kales scoring rules, and re-aligned to the AASM rules. For more information we refer to https://doi.org/10.13026/C2C30J.

Calculation of expected values from GAMLSS

In GAMLSS, distributional parameters are modeled using additive predictors and link functions. That is, each parameter θ is expressed as $\eta_\theta = g_\theta(\theta)$, where $g_\theta(\cdot)$ is a link function specific to that parameter and distribution. To compute interpretable quantities such as expectations, we apply the inverse link function: $\theta = g_\theta^{-1}(\eta_\theta)$.

Expected value formulas

For the zeros-and-ones-inflated Beta (BEINF) distribution, used for bounded performance metrics such as MF1, the expected value is:

$$\mathbb{E}[Y] = \tau + (1 - \nu - \tau) \cdot \mu$$

where:

- μ is the mean of the continuous Beta part,
- ν is the probability mass at 0,
- τ is the probability mass at 1.

These parameters are modeled using the following link functions:

$$\begin{aligned} \eta_\mu &= \text{logit}(\mu) && \Rightarrow && \mu = \text{logit}^{-1}(\eta_\mu) = \frac{1}{1 + \exp(-\eta_\mu)} \\ \eta_\nu &= \log(\nu) && \Rightarrow && \nu = \exp(\eta_\nu) \\ \eta_\tau &= \log(\tau) && \Rightarrow && \tau = \exp(\eta_\tau) \end{aligned}$$

For Gaussian-distributed bias outcomes (e.g., TST $= \widehat{\text{TST}}_{\text{algorithm}} - \text{TST}_{\text{reference}}$), the expectation is straightforward:

$$\mathbb{E}[Y] = \mu$$

with

$$\eta_\mu = \mu \quad \text{(identity link)}, \quad \Rightarrow \mu = \eta_\mu$$

Worked examples

We provide two examples using the SOMNUS model: one for a baseline subject and one for a non-baseline case. Refer to Table 4 and Table 5 in the manuscript to retrieve the values.

Example 1: Baseline subject (female, age 50, AHI = 0, PLMI = 0)

MF1 (performance metric):

The intercepts for MF1 are:

$$\eta_\mu = 1.12, \quad \eta_\nu = -22.54, \quad \eta_\tau = -22.63$$

Transforming back via the inverse link functions:

$$\mu = \text{logit}^{-1}(1.12) = \frac{1}{1 + \exp(-1.12)} \approx 0.754,$$

$$\nu = \exp(-22.54) \approx 1.62 \times 10^{-10},$$

$$\tau = \exp(-22.63) \approx 1.48 \times 10^{-10}$$

Then the expected value is:

$$\mathbb{E}[Y] \approx 1.48 \times 10^{-10} + (1 - 1.62 \times 10^{-10} - 1.48 \times 10^{-10}) \cdot 0.754 \approx 0.754$$

TST bias (in minutes):

The intercept for TST (Normal distribution) is:

$$\eta_\mu = -9.05 \quad \text{(identity link)} \quad \Rightarrow \quad \mathbb{E}[Y] = -9.05$$

Example 2: Male, age 50, AHI = 30, PLMI = 10

MF1 (performance metric):

The relevant coefficients are:

$$\beta_\mu^{\text{gender}} = -0.06, \quad \beta_\mu^{\text{AHI}} = -0.05, \quad \beta_\mu^{\text{PLMI}} = -0.02$$

The linear predictor becomes:

$$\eta_\mu = 1.12 - 0.06 - 3 \cdot 0.05 - 1 \cdot 0.02 = 0.89$$

$$\mu = \text{logit}^{-1}(0.89) = \frac{1}{1+\exp(-0.89)} \approx 0.709$$

With $\eta_\nu = -22.54$, $\eta_\tau = -22.63$ as before:

$$\mathbb{E}[Y] \approx \exp(-22.63) + (1 - \exp(-22.54) - \exp(-22.63)) \cdot 0.709 \approx 0.709$$

TST bias (in minutes):

Coefficients for the linear predictor:

$$\beta_\mu^{\text{gender}} = -1.37, \quad \beta_\mu^{\text{AHI}} = -1.95, \quad \beta_\mu^{\text{PLMI}} = -0.47$$

$$\eta_\mu = -9.05 - 1.37 - 3 \cdot 1.95 - 1 \cdot 0.47 = -16.74 \quad \Rightarrow \quad \mathbb{E}[Y] = -16.74$$

References

[1] Zhang, G.-Q., Cui, L., Mueller, R., Tao, S., Kim, M., Rueschman, M., Mariani, S., Mobley, D., Redline, S.: The national sleep research resource: towards a sleep data commons. Journal of the American Medical Informatics Association **25**(10), 1351–1358 (2018)

[2] Bakker, J.P., Tavakkoli, A., Rueschman, M., Wang, W., Andrews, R., Malhotra, A., Owens, R.L., Anand, A., Dudley, K.A., Patel, S.R.: Gastric banding surgery versus continuous positive airway pressure for obstructive sleep apnea: a randomized controlled trial. American journal of respiratory and critical care medicine **197**(8), 1080–1083 (2018)

[3] Moore IV, H., Leary, E., Lee, S.-Y., Carrillo, O., Stubbs, R., Peppard, P., Young, T., Widrow, B., Mignot, E.: Design and validation of a periodic leg movement detector. PloS one **9**(12), 114565 (2014)

[4] Quan, S.F., Chan, C.S., Dement, W.C., Gevins, A., Goodwin, J.L., Gottlieb, D.J., Green, S., Guilleminault, C., Hirshkowitz, M., Hyde, P.R., *et al.*: The association between obstructive sleep apnea and neurocognitive performance—the apnea positive pressure long-term efficacy study (apples). Sleep **34**(3), 303–314 (2011)

[5] Rosen, C.L., Larkin, E.K., Kirchner, H.L., Emancipator, J.L., Bivins, S.F., Surovec, S.A., Martin, R.J., Redline, S.: Prevalence and risk factors for sleep-disordered breathing in 8-to 11-year-old children: association with race and prematurity. The Journal of pediatrics **142**(4), 383–389 (2003)

[6] Redline, S., Tishler, P.V., Tosteson, T.D., Williamson, J., Kump, K., Browner, I., Ferrette, V., Krejci, P.: The familial aggregation of obstructive sleep apnea. American journal of respiratory and critical care medicine **151**(3), 682–687 (1995)

[7] Marcus, C.L., Moore, R.H., Rosen, C.L., Giordani, B., Garetz, S.L., Taylor, H.G., Mitchell, R.B., Amin, R., Katz, E.S., Arens, R., *et al.*: A randomized trial of adenotonsillectomy for childhood sleep apnea. N Engl J Med **368**, 2366–2376 (2013)

[8] Redline, S., Amin, R., Beebe, D., Chervin, R.D., Garetz, S.L., Giordani, B., Marcus, C.L., Moore, R.H., Rosen, C.L., Arens, R., *et al.*: The childhood adenotonsillectomy trial (chat): rationale, design, and challenges of a randomized controlled trial evaluating a standard surgical procedure in a pediatric population. Sleep **34**(11), 1509–1517 (2011)

[9] Rosen, C.L., Auckley, D., Benca, R., Foldvary-Schaefer, N., Iber, C., Kapur, V., Rueschman, M., Zee, P., Redline, S.: A multisite randomized trial of portable sleep studies and positive airway pressure autotitration versus laboratory-based polysomnography for the diagnosis and treatment of obstructive sleep apnea: the homepap study. Sleep **35**(6), 757–767 (2012)

[10] Chen, X., Wang, R., Zee, P., Lutsey, P.L., Javaheri, S., Alcántara, C., Jackson, C.L., Williams, M.A., Redline, S.: Racial/ethnic differences in sleep disturbances: the multi-ethnic study of atherosclerosis (mesa). Sleep **38**(6), 877–888 (2015)

[11] Stephansen, J.B., Olesen, A.N., Olsen, M., Ambati, A., Leary, E.B., Moore, H.E., Carrillo, O., Lin, L., Han, F., Yan, H., *et al.*: Neural network analysis of sleep stages enables efficient diagnosis of narcolepsy. Nature communications **9**(1), 5229 (2018)

[12] Blackwell, T., Yaffe, K., Ancoli-Israel, S., Redline, S., Ensrud, K.E., Stefanick, M.L., Laffan, A., Stone, K.L., in Men Study Group, O.F.: Associations between sleep architecture and sleep-disordered breathing and cognition in older community-dwelling men: the osteoporotic fractures in men sleep study. Journal of the American Geriatrics Society **59**(12), 2217–2225 (2011)

[13] Relationships between sleep stages and changes in cognitive function in older men: the mros sleep study. Sleep **38**(3), 411–421 (2015)

[14] DiPietro, J.A., Raghunathan, R.S., Wu, H.-T., Bai, J., Watson, H., Sgambati, F.P., Henderson, J.L., Pien, G.W.: Fetal heart rate during maternal sleep. Developmental Psychobiology **63**(5), 945–959 (2021)

[15] DiPietro, J.A., Bai, J., Sgambati, F.P., Henderson, J.L., Watson, H., Raghunathan, R.S., Pien, G.W.: Fetal heart rate responses to maternal sleep-disordered breathing. American journal of obstetrics and gynecology **228**(4), 459–1 (2023)

[16] Lee, H., Li, B., DeForte, S., Splaingard, M.L., Huang, Y., Chi, Y., Linwood, S.L.: A large collection of real-world pediatric sleep studies. Scientific Data **9**(1), 421 (2022)

[17] Quan, S.F., Howard, B.V., Iber, C., Kiley, J.P., Nieto, F.J., O'Connor, G.T., Rapoport, D.M., Redline, S., Robbins, J., Samet, J.M., *et al.*: The sleep heart health study: design, rationale, and methods. Sleep **20**(12), 1077–1085 (1997)

[18] Cummings, S.R., Black, D.M., Nevitt, M.C., Browner, W.S., Cauley, J.A., Genant, H.K., Mascioli, S.R., Scott, J.C., Seeley, D.G., Steiger, P., *et al.*: Appendicular bone density and age predict hip fracture in women. Jama **263**(5), 665–668 (1990)

[19] Spira, A.P., Blackwell, T., Stone, K.L., Redline, S., Cauley, J.A., Ancoli-Israel, S., Yaffe, K.: Sleep-disordered breathing and cognition in older women. Journal of the American Geriatrics Society **56**(1), 45–50 (2008)

[20] Young, T., Palta, M., Dempsey, J., Peppard, P.E., Nieto, F.J., Hla, K.M.: Burden of sleep apnea: rationale, design, and major findings of the wisconsin sleep cohort study. WMJ: official publication of the State Medical Society of Wisconsin **108**(5), 246 (2009)

[21] Berry, R.B., Brooks, R., Gamaldo, C., Harding, S.M., Lloyd, R.M., Quan, S.F., Troester, M.T., Vaughn, B.V.: AASM scoring manual updates for 2017 (version 2.4). American Academy of Sleep Medicine (2017)

[22] Perslev, M., Darkner, S., Kempfner, L., Nikolic, M., Jennum, P.J., Igel, C.: U-sleep: resilient high-frequency sleep staging. NPJ digital medicine **4**(1), 1–12 (2021)

[23] Guillot, A., Sauvet, F., During, E.H., Thorey, V.: Dreem open datasets: Multi-scored sleep datasets to compare human and automated sleep staging. IEEE transactions on neural systems and rehabilitation engineering **28**(9), 1955–1965 (2020)

[24] Goldberger, A.L., Amaral, L.A., Glass, L., Hausdorff, J.M., Ivanov, P.C., Mark, R.G., Mietus, J.E., Moody, G.B., Peng, C.-K., Stanley, H.E.: Physiobank, physiotoolkit, and physionet: components of a new research resource for complex physiologic signals. circulation **101**(23), 215–220 (2000)

[25] Ghassemi, M.M., Moody, B.E., Lehman, L.-W.H., Song, C., Li, Q., Sun, H., Mark, R.G., Westover, M.B., Clifford, G.D.: You snooze, you win: the physionet/computing in cardiology challenge 2018. In: 2018 Computing in Cardiology Conference (CinC), vol. 45, pp. 1–4 (2018). IEEE

[26] Kemp, B., Zwinderman, A.H., Tuk, B., Kamphuisen, H.A., Oberye, J.J.: Analysis of a sleep-dependent neuronal feedback loop: the slow-wave microcontinuity of the eeg. IEEE Transactions on Biomedical Engineering **47**(9), 1185–1194 (2000)

List of Supplementary Tables

Supplementary Table 1 EEG and EOG derivations available within each in-domain (ID) datasets.

Datasets	Channel type	Channel derivations
ABC	EEG	F3-M2, F4-M1, C3-M2, C4-M1, O1-M2, O2-M1
	EOG	E1-M2, E2-M1
APOE	EEG	C3-A1, C3-A2, C3-O1, C4-A1, C4-A2, F1-A2, F2-C4, F2-T4, FP1-C3, FP1-C3, FP2-C4, FP2-C4, Fz-A1, Fz-A2, O1-A2, O1-A2, O2-A1, T3-O1, T4-O2
	EOG	LOC-A2, ROC-A1, LOC, ROC
APPLES	EEG	C3-M2, C4-M1, O1-M2, O2-M1
	EOG	LOC, ROC
CCSHS	EEG	C3-A2, C4-A1
	EOG	LOC-A2, ROC-A1
CFS	EEG	C3-A2, C4-A1
	EOG	LOC-A2, ROC-A1
CHAT	EEG	F3-M2, F4-M1, C3-M2, C4-M1, T3-M2, T4-M1, O1-M2, O2-M1
	EOG	E1-M2, E2-M1, E1, E2
HOMEPAP	EEG	F4-M1, C4-M1, O2-M1, C3-M2, F3-M2, O1-M2, F4, C4, O2, C3, F3, O1
	EOG	E1-M2, E2-M1, E1, E2, LOC, ROC, E1-E2
MESA	EEG	Fpz-Cz, Cz-Oz, C4-M1
	EOG	E1-Fpz, E2-Fpz
MNC	EEG	C3-M2, C3, C4-M1, C4, Cz, F3-M2, F3, F4-M1, F4, O1-M2, O1, O2-M1, O2
	EOG	E1-M2 E1 E2-M1 E2
MROS	EEG	C3-M2, C4-M1
	EOG	E1-M2, E2-M1, E1, E2
MSP	EEG	C3-M2, C4-M1, F3-M2, F4-M1, O1-M2, O2-M1
	EOG	LOC, ROC
NCHSDB	EEG	FP1, FP2, FZ, CZ, PZ, OZ, FPZ, P3-M2, P4-M1, F3-M2, F4-M1, F4-M2, C3-M2, C4-M1, C4-M2, T3-M2, T4-M1, O1-M2, O2-M1, F4, O1, O2
	EOG	E1-M2, E2-M1, E1-M1, E2-M2, E1, E2
SHHS	EEG	C4-A1, C3-A2
	EOG	EOGL-PG1, EOGR-PG1
SOF	EEG	C3-A2, C4-A1
	EOG	LOC-A2, ROC-A1
WSC	EEG	F3-M1, F3-M2, F3-AVG, F4-M1, F4-M2, F4-AVG, Fz-M1, Fz-M2, Fz-AVG, Cz-M1, Cz-M2, Cz-AVG, C3-M1, C3-M2, C3-AVG, C4-M1, C4-M2, C4-AVG, Pz-M1, Pz-M2, Pz-AVG, Pz-Cz, O1-M1, O1-M2, O1-AVG, O2-M1, O2-M2, O2-AVG
	EOG	E1, E2

Supplementary Table 2 Data split on the in-domain (ID) datasets. We report the total number of recordings, and the number of recordings used to train, validate and test all the models in SLEEPYLAND.

Datasets	Recordings	Train	Valid	Test
ABC	132	97	13	22
APOE	712	562	50	100
APPLES	1094	944	50	100
CCSHS	515	387	50	78
CFS	730	545	98	87
CHAT	1638	1439	60	139
HOMEPAP	246	184	25	37
MESA	2056	1906	50	100
MNC-CNC	78	58	8	12
MNC-DHC	83	61	9	13
MNC-SSC	767	617	50	100
MROS	3930	3735	67	128
MSP	105	78	11	16
NCHSDB	3950	3789	55	106
SHHS	8444	8223	75	146
SOF	453	339	46	68
WSC	2569	2222	119	228

Supplementary Table 3 SOMNUS dataset-wise performance overview. MF1, Accuracy and Cohen's κ, plus Class-Wise F1 score computed across all test recordings in each dataset (majority voting across channel derivations).

**ID Dataset**	**MF1**	**Acc**	κ	**W**	**N1**	**N2**	**N3**	**REM**
ABC	0.825	0.854	0.796	0.907	0.629	0.878	0.775	0.934
APOE	0.752	0.822	0.739	0.892	0.448	0.862	0.671	0.888
APPLES	0.731	0.835	0.761	0.923	0.516	0.868	0.443	0.906
CCSHS	0.875	0.927	0.899	0.974	0.655	0.921	0.887	0.939
CFS	0.827	0.906	0.865	0.964	0.522	0.895	0.836	0.916
CHAT	0.848	0.890	0.855	0.969	0.600	0.866	0.900	0.905
HOMEPAP	0.775	0.830	0.766	0.910	0.414	0.841	0.798	0.911
MESA	0.790	0.877	0.820	0.958	0.523	0.861	0.694	0.913
MNC-CNC	0.813	0.837	0.783	0.861	0.598	0.856	0.860	0.890
MNC-DHC	0.818	0.914	0.862	0.979	0.518	0.864	0.831	0.896
MNC-SSC	0.728	0.815	0.724	0.841	0.348	0.866	0.704	0.881
MROS	0.794	0.900	0.847	0.962	0.495	0.887	0.720	0.907
MSP	0.807	0.881	0.825	0.951	0.530	0.896	0.757	0.904
NCHSDB	0.789	0.874	0.827	0.904	0.382	0.875	0.909	0.876
SHHS	0.803	0.881	0.833	0.947	0.483	0.878	0.793	0.912
SOF	0.794	0.891	0.840	0.961	0.457	0.865	0.759	0.929
WSC	0.780	0.865	0.792	0.905	0.514	0.910	0.665	0.904
**OOD Dataset**	**MF1**	**Acc**	κ	**W**	**N1**	**N2**	**N3**	**REM**
BSWR	0.755	0.794	0.720	0.867	0.414	0.827	0.771	0.897
DCSM	0.818	0.928	0.872	0.984	0.506	0.865	0.824	0.908
DOD-H	0.857	0.903	0.861	0.923	0.617	0.917	0.881	0.949
DOD-O	0.816	0.878	0.821	0.929	0.526	0.897	0.795	0.935
PHYS	0.734	0.782	0.699	0.790	0.393	0.850	0.752	0.887
SEDF-SC	0.754	0.920	0.835	0.984	0.354	0.837	0.712	0.882
SEDF-ST	0.775	0.838	0.767	0.828	0.475	0.876	0.798	0.898
**Avg ID**	0.797	0.871	0.814	0.930	0.508	0.876	0.765	0.907
**Avg OOD**	0.787	0.863	0.796	0.901	0.469	0.867	0.790	0.908

Supplementary Table 4 SOMNUS$_{\textbf{U-Sleep}}$ benchmarking. MF1, Accuracy and Cohen's κ, plus Class-Wise F1 score on recording-level for U-Sleep considering both single and multi-channel configurations.

**ID Dataset**	**MF1**	**Acc**	κ	**W**	**N1**	**N2**	**N3**	**REM**
ABC	0.776 ± 0.094	0.847 ± 0.051	0.773 ± 0.063	0.888 ± 0.047	0.594 ± 0.084	0.862 ± 0.072	0.621 ± 0.306	0.895 ± 0.201
CCSHS	0.865 ± 0.052	0.923 ± 0.038	0.892 ± 0.053	0.969 ± 0.022	0.624 ± 0.151	0.916 ± 0.050	0.878 ± 0.099	0.936 ± 0.035
CFS	0.813 ± 0.081	0.904 ± 0.060	0.854 ± 0.098	0.963 ± 0.037	0.536 ± 0.153	0.885 ± 0.099	0.777 ± 0.222	0.905 ± 0.106
CHAT	0.830 ± 0.044	0.883 ± 0.047	0.842 ± 0.063	0.955 ± 0.033	0.551 ± 0.114	0.853 ± 0.080	0.895 ± 0.072	0.897 ± 0.060
HOMEPAP	0.750 ± 0.084	0.829 ± 0.084	0.747 ± 0.133	0.901 ± 0.082	0.406 ± 0.151	0.824 ± 0.095	0.750 ± 0.228	0.894 ± 0.125
MESA	0.743 ± 0.088	0.866 ± 0.074	0.798 ± 0.115	0.945 ± 0.075	0.492 ± 0.137	0.845 ± 0.109	0.528 ± 0.293	0.901 ± 0.077
MROS	0.749 ± 0.085	0.894 ± 0.041	0.831 ± 0.067	0.957 ± 0.037	0.450 ± 0.159	0.875 ± 0.063	0.575 ± 0.281	0.880 ± 0.142
SHHS	0.770 ± 0.087	0.873 ± 0.066	0.813 ± 0.096	0.934 ± 0.070	0.452 ± 0.197	0.867 ± 0.080	0.700 ± 0.222	0.903 ± 0.087
SOF	0.765 ± 0.071	0.882 ± 0.060	0.814 ± 0.094	0.951 ± 0.045	0.411 ± 0.154	0.849 ± 0.084	0.696 ± 0.184	0.920 ± 0.072
APOE	0.721 ± 0.104	0.816 ± 0.087	0.717 ± 0.127	0.869 ± 0.082	0.434 ± 0.178	0.849 ± 0.103	0.567 ± 0.316	0.862 ± 0.139
APPLES	0.739 ± 0.092	0.828 ± 0.085	0.743 ± 0.114	0.910 ± 0.051	0.493 ± 0.150	0.839 ± 0.138	0.413 ± 0.338	0.871 ± 0.155
MNC-SSC	0.683 ± 0.107	0.811 ± 0.076	0.704 ± 0.119	0.805 ± 0.125	0.309 ± 0.177	0.859 ± 0.065	0.581 ± 0.312	0.852 ± 0.169
MNC-DHC	0.815 ± 0.058	0.915 ± 0.039	0.861 ± 0.061	0.979 ± 0.015	0.524 ± 0.139	0.854 ± 0.074	0.813 ± 0.101	0.906 ± 0.043
MNC-CNC	0.768 ± 0.067	0.824 ± 0.054	0.757 ± 0.068	0.796 ± 0.154	0.496 ± 0.156	0.826 ± 0.109	0.847 ± 0.073	0.873 ± 0.048
MSP	0.779 ± 0.084	0.877 ± 0.039	0.803 ± 0.068	0.918 ± 0.070	0.494 ± 0.097	0.887 ± 0.054	0.688 ± 0.269	0.883 ± 0.138
WSC	0.724 ± 0.100	0.858 ± 0.073	0.771 ± 0.114	0.887 ± 0.112	0.473 ± 0.165	0.902 ± 0.058	0.450 ± 0.293	0.879 ± 0.137
NCHSDB	0.753 ± 0.076	0.869 ± 0.043	0.801 ± 0.098	0.856 ± 0.105	0.324 ± 0.167	0.853 ± 0.125	0.897 ± 0.095	0.840 ± 0.157
**OOD Dataset**	**MF1**	**Acc**	κ	**W**	**N1**	**N2**	**N3**	**REM**
DCSM	0.797 ± 0.082	0.924 ± 0.044	0.862 ± 0.077	0.983 ± 0.022	0.500 ± 0.149	0.852 ± 0.098	0.760 ± 0.205	0.895 ± 0.130
SEDF-ST	0.751 ± 0.073	0.834 ± 0.051	0.756 ± 0.073	0.788 ± 0.114	0.466 ± 0.143	0.868 ± 0.057	0.734 ± 0.237	0.899 ± 0.080
SEDF-SC	0.733 ± 0.083	0.917 ± 0.038	0.827 ± 0.074	0.982 ± 0.015	0.366 ± 0.136	0.829 ± 0.083	0.612 ± 0.276	0.861 ± 0.110
PHYS	0.688 ± 0.099	0.778 ± 0.089	0.672 ± 0.124	0.746 ± 0.161	0.391 ± 0.155	0.831 ± 0.103	0.630 ± 0.275	0.853 ± 0.167
DOD-H	0.819 ± 0.074	0.885 ± 0.046	0.828 ± 0.071	0.876 ± 0.133	0.566 ± 0.166	0.892 ± 0.053	0.823 ± 0.163	0.934 ± 0.057
DOD-O	0.783 ± 0.078	0.876 ± 0.058	0.805 ± 0.087	0.920 ± 0.055	0.534 ± 0.138	0.890 ± 0.061	0.649 ± 0.289	0.925 ± 0.071
BSWR	0.711 ± 0.112	0.788 ± 0.100	0.687 ± 0.139	0.834 ± 0.139	0.429 ± 0.167	0.803 ± 0.126	0.629 ± 0.290	0.863 ± 0.171
**Avg ID**	0.767 ± 0.044	0.865 ± 0.035	0.795 ± 0.052	0.911 ± 0.055	0.474 ± 0.084	0.861 ± 0.025	0.687 ± 0.153	0.888 ± 0.024
**Avg OOD**	0.755 ± 0.048	0.857 ± 0.059	0.777 ± 0.074	0.876 ± 0.092	0.465 ± 0.074	0.852 ± 0.033	0.691 ± 0.081	0.890 ± 0.032

Supplementary Table 5 **SOMNUS$_{\text{DeepResNet}}$ benchmarking.** MF1, Accuracy and Cohen's κ, plus Class-Wise F1 score on recording-level for DeepResNet considering both single and multi-channel configurations.

**ID Dataset**	**MF1**	**Acc**	κ	**W**	**N1**	**N2**	**N3**	**REM**
ABC	0.776 ± 0.094	0.844 ± 0.051	0.769 ± 0.062	0.887 ± 0.046	0.596 ± 0.088	0.857 ± 0.077	0.633 ± 0.298	0.886 ± 0.200
CCSHS	0.861 ± 0.049	0.921 ± 0.037	0.889 ± 0.051	0.970 ± 0.018	0.613 ± 0.141	0.914 ± 0.049	0.878 ± 0.091	0.930 ± 0.039
CFS	0.807 ± 0.085	0.903 ± 0.062	0.852 ± 0.101	0.962 ± 0.040	0.509 ± 0.180	0.885 ± 0.099	0.770 ± 0.236	0.912 ± 0.082
CHAT	0.827 ± 0.048	0.880 ± 0.048	0.838 ± 0.065	0.953 ± 0.039	0.546 ± 0.125	0.852 ± 0.080	0.897 ± 0.072	0.887 ± 0.063
HOMEPAP	0.734 ± 0.135	0.809 ± 0.127	0.722 ± 0.177	0.888 ± 0.117	0.402 ± 0.155	0.794 ± 0.158	0.720 ± 0.277	0.884 ± 0.170
MESA	0.743 ± 0.090	0.869 ± 0.069	0.801 ± 0.110	0.950 ± 0.061	0.504 ± 0.141	0.848 ± 0.108	0.512 ± 0.305	0.898 ± 0.072
MROS	0.740 ± 0.085	0.893 ± 0.041	0.829 ± 0.066	0.955 ± 0.036	0.414 ± 0.157	0.875 ± 0.058	0.576 ± 0.290	0.874 ± 0.144
SHHS	0.766 ± 0.089	0.872 ± 0.068	0.812 ± 0.099	0.931 ± 0.074	0.440 ± 0.203	0.866 ± 0.081	0.699 ± 0.233	0.900 ± 0.085
SOF	0.754 ± 0.073	0.879 ± 0.061	0.811 ± 0.094	0.949 ± 0.048	0.372 ± 0.159	0.848 ± 0.083	0.695 ± 0.185	0.913 ± 0.079
APOE	0.717 ± 0.104	0.814 ± 0.086	0.713 ± 0.127	0.869 ± 0.079	0.429 ± 0.173	0.847 ± 0.100	0.557 ± 0.315	0.860 ± 0.140
APPLES	0.730 ± 0.092	0.823 ± 0.091	0.735 ± 0.119	0.907 ± 0.054	0.478 ± 0.159	0.835 ± 0.144	0.373 ± 0.304	0.873 ± 0.145
MNC-SSC	0.677 ± 0.104	0.804 ± 0.079	0.693 ± 0.120	0.801 ± 0.125	0.302 ± 0.164	0.852 ± 0.070	0.570 ± 0.305	0.851 ± 0.165
MNC-DHC	0.800 ± 0.065	0.908 ± 0.044	0.849 ± 0.071	0.975 ± 0.021	0.479 ± 0.156	0.845 ± 0.076	0.815 ± 0.101	0.886 ± 0.064
MNC-CNC	0.759 ± 0.063	0.816 ± 0.051	0.747 ± 0.064	0.775 ± 0.160	0.484 ± 0.144	0.824 ± 0.098	0.842 ± 0.069	0.870 ± 0.052
MSP	0.776 ± 0.072	0.877 ± 0.030	0.804 ± 0.052	0.920 ± 0.059	0.496 ± 0.119	0.886 ± 0.053	0.660 ± 0.259	0.893 ± 0.113
WSC	0.728 ± 0.100	0.858 ± 0.073	0.771 ± 0.113	0.885 ± 0.108	0.480 ± 0.165	0.901 ± 0.058	0.467 ± 0.289	0.877 ± 0.142
NCHSDB	0.746 ± 0.077	0.864 ± 0.051	0.796 ± 0.106	0.847 ± 0.106	0.305 ± 0.166	0.851 ± 0.120	0.895 ± 0.095	0.839 ± 0.156
**OOD Dataset**	**MF1**	**Acc**	κ	**W**	**N1**	**N2**	**N3**	**REM**
DCSM	0.794 ± 0.084	0.922 ± 0.045	0.858 ± 0.079	0.982 ± 0.024	0.496 ± 0.149	0.845 ± 0.100	0.760 ± 0.212	0.892 ± 0.134
SEDF-ST	0.758 ± 0.072	0.836 ± 0.052	0.759 ± 0.073	0.792 ± 0.109	0.492 ± 0.153	0.869 ± 0.059	0.736 ± 0.236	0.898 ± 0.078
SEDF-SC	0.733 ± 0.082	0.911 ± 0.042	0.818 ± 0.076	0.976 ± 0.023	0.403 ± 0.143	0.833 ± 0.077	0.608 ± 0.277	0.826 ± 0.127
PHYS	0.687 ± 0.097	0.777 ± 0.086	0.671 ± 0.122	0.741 ± 0.161	0.385 ± 0.151	0.832 ± 0.101	0.640 ± 0.268	0.849 ± 0.168
DOD-H	0.820 ± 0.076	0.897 ± 0.041	0.844 ± 0.066	0.868 ± 0.138	0.539 ± 0.160	0.909 ± 0.043	0.857 ± 0.159	0.928 ± 0.072
DOD-O	0.784 ± 0.077	0.875 ± 0.058	0.805 ± 0.088	0.920 ± 0.055	0.520 ± 0.132	0.891 ± 0.060	0.668 ± 0.277	0.925 ± 0.071
BSWR	0.706 ± 0.111	0.785 ± 0.100	0.681 ± 0.140	0.829 ± 0.140	0.423 ± 0.161	0.802 ± 0.126	0.620 ± 0.286	0.862 ± 0.169
**Avg ID**	0.761 ± 0.044	0.861 ± 0.037	0.790 ± 0.055	0.907 ± 0.059	0.462 ± 0.087	0.858 ± 0.029	0.680 ± 0.157	0.884 ± 0.023
**Avg OOD**	0.755 ± 0.048	0.858 ± 0.059	0.777 ± 0.076	0.873 ± 0.092	0.465 ± 0.061	0.854 ± 0.037	0.698 ± 0.090	0.883 ± 0.039

Supplementary Table 6 SOMNUS$_{\text{SleepTransformer}}$ benchmarking. MF1, Accuracy and Cohen's κ, plus Class-Wise F1 score on recording-level for SleepTransformer considering both single and multi-channel configurations.

**ID Dataset**	**MF1**	**Acc**	κ	**W**	**N1**	**N2**	**N3**	**REM**
ABC	0.790 ± 0.084	0.851 ± 0.042	0.780 ± 0.048	0.892 ± 0.047	0.606 ± 0.090	0.862 ± 0.066	0.683 ± 0.272	0.891 ± 0.200
CCSHS	0.866 ± 0.049	0.923 ± 0.033	0.893 ± 0.047	0.973 ± 0.016	0.629 ± 0.148	0.915 ± 0.047	0.876 ± 0.097	0.935 ± 0.038
CFS	0.811 ± 0.081	0.901 ± 0.062	0.850 ± 0.101	0.962 ± 0.039	0.542 ± 0.154	0.880 ± 0.101	0.764 ± 0.228	0.910 ± 0.085
CHAT	0.848 ± 0.039	0.889 ± 0.042	0.850 ± 0.057	0.963 ± 0.030	0.622 ± 0.097	0.855 ± 0.076	0.897 ± 0.071	0.903 ± 0.054
HOMEPAP	0.760 ± 0.082	0.833 ± 0.079	0.750 ± 0.128	0.910 ± 0.073	0.452 ± 0.143	0.824 ± 0.097	0.734 ± 0.234	0.900 ± 0.119
MESA	0.751 ± 0.091	0.873 ± 0.069	0.807 ± 0.110	0.953 ± 0.061	0.509 ± 0.142	0.850 ± 0.105	0.540 ± 0.297	0.901 ± 0.079
MROS	0.751 ± 0.086	0.896 ± 0.037	0.835 ± 0.060	0.957 ± 0.036	0.464 ± 0.162	0.877 ± 0.054	0.570 ± 0.289	0.875 ± 0.150
SHHS	0.777 ± 0.095	0.878 ± 0.067	0.820 ± 0.105	0.932 ± 0.083	0.454 ± 0.210	0.869 ± 0.081	0.742 ± 0.223	0.899 ± 0.095
SOF	0.770 ± 0.075	0.884 ± 0.059	0.819 ± 0.094	0.948 ± 0.051	0.423 ± 0.164	0.851 ± 0.078	0.711 ± 0.209	0.925 ± 0.067
APOE	0.729 ± 0.097	0.826 ± 0.078	0.732 ± 0.117	0.873 ± 0.076	0.442 ± 0.167	0.859 ± 0.092	0.578 ± 0.330	0.870 ± 0.123
APPLES	0.740 ± 0.096	0.839 ± 0.067	0.757 ± 0.094	0.912 ± 0.053	0.529 ± 0.152	0.849 ± 0.131	0.306 ± 0.322	0.871 ± 0.152
MNC-SSC	0.692 ± 0.104	0.819 ± 0.073	0.717 ± 0.115	0.813 ± 0.124	0.320 ± 0.170	0.867 ± 0.063	0.598 ± 0.307	0.855 ± 0.166
MNC-DHC	0.811 ± 0.057	0.912 ± 0.042	0.857 ± 0.067	0.978 ± 0.014	0.513 ± 0.135	0.847 ± 0.093	0.822 ± 0.087	0.894 ± 0.055
MNC-CNC	0.783 ± 0.066	0.833 ± 0.060	0.772 ± 0.072	0.805 ± 0.151	0.550 ± 0.124	0.827 ± 0.106	0.855 ± 0.074	0.879 ± 0.063
MSP	0.778 ± 0.078	0.875 ± 0.036	0.803 ± 0.061	0.927 ± 0.061	0.502 ± 0.088	0.883 ± 0.052	0.664 ± 0.275	0.887 ± 0.101
WSC	0.743 ± 0.098	0.860 ± 0.070	0.777 ± 0.110	0.898 ± 0.102	0.519 ± 0.153	0.899 ± 0.056	0.489 ± 0.283	0.886 ± 0.140
NCHSDB	0.753 ± 0.077	0.868 ± 0.044	0.801 ± 0.096	0.853 ± 0.119	0.329 ± 0.165	0.850 ± 0.129	0.895 ± 0.076	0.845 ± 0.146
**OOD Dataset**	**MF1**	**Acc**	κ	**W**	**N1**	**N2**	**N3**	**REM**
DCSM	0.800 ± 0.083	0.927 ± 0.044	0.866 ± 0.079	0.982 ± 0.024	0.474 ± 0.154	0.857 ± 0.095	0.803 ± 0.189	0.890 ± 0.147
SEDF-ST	0.747 ± 0.080	0.833 ± 0.051	0.752 ± 0.076	0.789 ± 0.112	0.469 ± 0.152	0.868 ± 0.055	0.711 ± 0.251	0.898 ± 0.084
SEDF-SC	0.719 ± 0.088	0.913 ± 0.040	0.818 ± 0.076	0.982 ± 0.013	0.346 ± 0.147	0.813 ± 0.098	0.559 ± 0.288	0.874 ± 0.092
PHYS	0.691 ± 0.098	0.777 ± 0.089	0.673 ± 0.126	0.740 ± 0.162	0.379 ± 0.156	0.830 ± 0.105	0.673 ± 0.257	0.848 ± 0.169
DOD-H	0.833 ± 0.062	0.901 ± 0.034	0.852 ± 0.057	0.885 ± 0.086	0.570 ± 0.158	0.914 ± 0.038	0.858 ± 0.162	0.940 ± 0.047
DOD-O	0.721 ± 0.099	0.810 ± 0.085	0.710 ± 0.117	0.822 ± 0.126	0.469 ± 0.153	0.849 ± 0.078	0.699 ± 0.267	0.770 ± 0.178
BSWR	0.710 ± 0.116	0.790 ± 0.106	0.692 ± 0.147	0.827 ± 0.144	0.380 ± 0.181	0.806 ± 0.129	0.684 ± 0.289	0.862 ± 0.172
**Avg ID**	0.774 ± 0.043	0.868 ± 0.031	0.801 ± 0.048	0.915 ± 0.053	0.494 ± 0.088	0.863 ± 0.023	0.690 ± 0.162	0.890 ± 0.023
**Avg OOD**	0.746 ± 0.052	0.850 ± 0.062	0.766 ± 0.079	0.861 ± 0.093	0.441 ± 0.077	0.848 ± 0.037	0.712 ± 0.096	0.869 ± 0.053

Supplementary Table 7 SOMNUS benchmarking. MF1, Accuracy and Cohen's κ, plus Class-Wise F1 score on recording-level for soft unweighted voting ensemble considering all deep learning scorers in SLEEPYLAND.

**ID Dataset**	**MF1**	**Acc**	κ	**W**	**N1**	**N2**	**N3**	**REM**
ABC	0.789 ± 0.087	0.853 ± 0.046	0.783 ± 0.054	0.895 ± 0.045	0.605 ± 0.087	0.866 ± 0.068	0.666 ± 0.288	0.895 ± 0.201
CCSHS	0.872 ± 0.049	0.926 ± 0.036	0.896 ± 0.050	0.973 ± 0.017	0.649 ± 0.141	0.919 ± 0.048	0.881 ± 0.094	0.938 ± 0.035
CFS	0.817 ± 0.080	0.906 ± 0.061	0.857 ± 0.099	0.963 ± 0.039	0.539 ± 0.162	0.888 ± 0.099	0.781 ± 0.223	0.916 ± 0.082
CHAT	0.842 ± 0.043	0.889 ± 0.045	0.849 ± 0.061	0.960 ± 0.031	0.589 ± 0.114	0.858 ± 0.078	0.899 ± 0.072	0.901 ± 0.057
HOMEPAP	0.755 ± 0.083	0.833 ± 0.082	0.751 ± 0.131	0.908 ± 0.078	0.416 ± 0.155	0.827 ± 0.091	0.744 ± 0.253	0.904 ± 0.111
MESA	0.752 ± 0.092	0.875 ± 0.069	0.809 ± 0.112	0.953 ± 0.061	0.510 ± 0.146	0.852 ± 0.111	0.536 ± 0.297	0.906 ± 0.071
MROS	0.752 ± 0.084	0.898 ± 0.038	0.838 ± 0.061	0.958 ± 0.035	0.459 ± 0.161	0.880 ± 0.055	0.575 ± 0.291	0.880 ± 0.143
SHHS	0.779 ± 0.088	0.879 ± 0.065	0.822 ± 0.095	0.935 ± 0.075	0.461 ± 0.207	0.873 ± 0.076	0.725 ± 0.217	0.904 ± 0.089
SOF	0.771 ± 0.071	0.887 ± 0.059	0.823 ± 0.092	0.951 ± 0.047	0.416 ± 0.158	0.856 ± 0.080	0.710 ± 0.193	0.928 ± 0.071
APOE	0.727 ± 0.104	0.822 ± 0.084	0.726 ± 0.124	0.877 ± 0.076	0.438 ± 0.180	0.855 ± 0.099	0.576 ± 0.321	0.865 ± 0.136
APPLES	0.740 ± 0.093	0.835 ± 0.081	0.752 ± 0.110	0.912 ± 0.051	0.506 ± 0.157	0.846 ± 0.135	0.361 ± 0.325	0.874 ± 0.150
MNC-SSC	0.687 ± 0.107	0.816 ± 0.076	0.710 ± 0.119	0.807 ± 0.126	0.312 ± 0.173	0.863 ± 0.066	0.592 ± 0.314	0.855 ± 0.168
MNC-DHC	0.813 ± 0.061	0.914 ± 0.041	0.859 ± 0.065	0.978 ± 0.014	0.516 ± 0.144	0.851 ± 0.083	0.822 ± 0.091	0.897 ± 0.054
MNC-CNC	0.786 ± 0.061	0.839 ± 0.052	0.778 ± 0.064	0.805 ± 0.150	0.536 ± 0.130	0.838 ± 0.102	0.862 ± 0.067	0.891 ± 0.040
MSP	0.787 ± 0.076	0.881 ± 0.033	0.810 ± 0.058	0.928 ± 0.056	0.520 ± 0.097	0.888 ± 0.053	0.680 ± 0.264	0.894 ± 0.115
WSC	0.740 ± 0.098	0.864 ± 0.071	0.781 ± 0.111	0.895 ± 0.106	0.500 ± 0.166	0.905 ± 0.056	0.486 ± 0.290	0.884 ± 0.139
NCHSDB	0.759 ± 0.075	0.875 ± 0.042	0.810 ± 0.096	0.860 ± 0.106	0.335 ± 0.165	0.856 ± 0.126	0.898 ± 0.094	0.853 ± 0.146
**OOD Dataset**	**MF1**	**Acc**	κ	**W**	**N1**	**N2**	**N3**	**REM**
DCSM	0.803 ± 0.083	0.927 ± 0.044	0.867 ± 0.077	0.983 ± 0.024	0.500 ± 0.151	0.857 ± 0.097	0.786 ± 0.196	0.895 ± 0.144
SEDF-ST	0.756 ± 0.076	0.838 ± 0.053	0.761 ± 0.076	0.793 ± 0.108	0.481 ± 0.153	0.872 ± 0.057	0.733 ± 0.243	0.902 ± 0.079
SEDF-SC	0.740 ± 0.081	0.920 ± 0.036	0.834 ± 0.068	0.984 ± 0.012	0.375 ± 0.140	0.833 ± 0.082	0.610 ± 0.281	0.880 ± 0.085
PHYS	0.694 ± 0.097	0.783 ± 0.087	0.679 ± 0.123	0.744 ± 0.160	0.387 ± 0.156	0.837 ± 0.102	0.660 ± 0.267	0.855 ± 0.166
DOD-H	0.836 ± 0.066	0.905 ± 0.034	0.857 ± 0.056	0.893 ± 0.094	0.572 ± 0.171	0.915 ± 0.038	0.857 ± 0.163	0.942 ± 0.048
DOD-O	0.792 ± 0.079	0.879 ± 0.060	0.812 ± 0.091	0.921 ± 0.056	0.526 ± 0.135	0.892 ± 0.062	0.703 ± 0.276	0.927 ± 0.070
BSWR	0.716 ± 0.112	0.794 ± 0.101	0.696 ± 0.141	0.834 ± 0.140	0.410 ± 0.171	0.811 ± 0.124	0.667 ± 0.282	0.868 ± 0.169
**Avg ID**	0.775 ± 0.045	0.870 ± 0.033	0.803 ± 0.050	0.915 ± 0.054	0.489 ± 0.089	0.866 ± 0.024	0.694 ± 0.157	0.893 ± 0.023
**Avg OOD**	0.762 ± 0.051	0.864 ± 0.059	0.787 ± 0.076	0.879 ± 0.093	0.464 ± 0.075	0.860 ± 0.036	0.717 ± 0.084	0.896 ± 0.031

Supplementary Table 8 U-Sleep$_{\mathbf{EEG+EOG}}$ benchmarking. MF1, Accuracy and Cohen's κ, plus Class-Wise F1 score on recording-level for U-Sleep using both EEG and EOG channel derivations.

**ID Dataset**	**MF1**	**Acc**	κ	**W**	**N1**	**N2**	**N3**	**REM**
ABC	0.785 ± 0.092	0.848 ± 0.050	0.776 ± 0.061	0.887 ± 0.046	0.612 ± 0.079	0.863 ± 0.069	0.648 ± 0.285	0.895 ± 0.201
CCSHS	0.859 ± 0.050	0.919 ± 0.038	0.886 ± 0.053	0.965 ± 0.033	0.611 ± 0.141	0.912 ± 0.048	0.879 ± 0.093	0.930 ± 0.038
CFS	0.809 ± 0.079	0.901 ± 0.060	0.851 ± 0.097	0.962 ± 0.037	0.520 ± 0.156	0.883 ± 0.097	0.780 ± 0.215	0.902 ± 0.110
CHAT	0.822 ± 0.047	0.881 ± 0.047	0.838 ± 0.063	0.952 ± 0.036	0.520 ± 0.121	0.851 ± 0.078	0.896 ± 0.071	0.892 ± 0.063
HOMEPAP	0.739 ± 0.100	0.821 ± 0.092	0.737 ± 0.143	0.890 ± 0.095	0.417 ± 0.152	0.811 ± 0.117	0.726 ± 0.260	0.878 ± 0.167
MESA	0.742 ± 0.089	0.859 ± 0.079	0.788 ± 0.117	0.937 ± 0.095	0.511 ± 0.128	0.838 ± 0.103	0.531 ± 0.299	0.889 ± 0.095
MROS	0.752 ± 0.080	0.891 ± 0.040	0.826 ± 0.067	0.955 ± 0.038	0.445 ± 0.150	0.870 ± 0.067	0.605 ± 0.250	0.879 ± 0.140
SHHS	0.768 ± 0.086	0.871 ± 0.065	0.811 ± 0.094	0.933 ± 0.067	0.448 ± 0.187	0.861 ± 0.083	0.703 ± 0.226	0.901 ± 0.087
SOF	0.766 ± 0.068	0.880 ± 0.056	0.813 ± 0.090	0.945 ± 0.049	0.408 ± 0.147	0.849 ± 0.079	0.718 ± 0.176	0.918 ± 0.071
APOE	0.718 ± 0.102	0.812 ± 0.087	0.711 ± 0.126	0.867 ± 0.081	0.447 ± 0.171	0.844 ± 0.106	0.537 ± 0.318	0.867 ± 0.132
APPLES	0.740 ± 0.086	0.831 ± 0.077	0.746 ± 0.105	0.906 ± 0.053	0.509 ± 0.142	0.842 ± 0.135	0.386 ± 0.313	0.873 ± 0.149
MNC-SSC	0.675 ± 0.104	0.804 ± 0.080	0.693 ± 0.120	0.801 ± 0.126	0.321 ± 0.172	0.852 ± 0.075	0.538 ± 0.305	0.853 ± 0.165
MNC-DHC	0.815 ± 0.060	0.914 ± 0.039	0.861 ± 0.061	0.978 ± 0.016	0.535 ± 0.139	0.854 ± 0.072	0.806 ± 0.129	0.904 ± 0.041
MNC-CNC	0.762 ± 0.065	0.822 ± 0.054	0.754 ± 0.068	0.786 ± 0.153	0.480 ± 0.139	0.825 ± 0.112	0.846 ± 0.074	0.873 ± 0.054
MSP	0.773 ± 0.080	0.874 ± 0.036	0.797 ± 0.066	0.914 ± 0.072	0.488 ± 0.108	0.884 ± 0.058	0.680 ± 0.266	0.876 ± 0.146
WSC	0.720 ± 0.097	0.853 ± 0.074	0.764 ± 0.113	0.882 ± 0.112	0.488 ± 0.159	0.897 ± 0.062	0.415 ± 0.286	0.883 ± 0.133
NCHSDB	0.745 ± 0.074	0.861 ± 0.045	0.790 ± 0.094	0.851 ± 0.103	0.320 ± 0.160	0.843 ± 0.132	0.888 ± 0.104	0.827 ± 0.157
**OOD Dataset**	**MF1**	**Acc**	κ	**W**	**N1**	**N2**	**N3**	**REM**
DCSM	0.797 ± 0.082	0.924 ± 0.043	0.861 ± 0.076	0.982 ± 0.022	0.495 ± 0.143	0.850 ± 0.094	0.771 ± 0.205	0.892 ± 0.134
SEDF-ST	0.744 ± 0.076	0.828 ± 0.055	0.748 ± 0.077	0.777 ± 0.126	0.451 ± 0.140	0.861 ± 0.064	0.733 ± 0.239	0.894 ± 0.083
SEDF-SC	0.732 ± 0.088	0.914 ± 0.037	0.821 ± 0.073	0.979 ± 0.015	0.388 ± 0.130	0.824 ± 0.089	0.598 ± 0.289	0.855 ± 0.117
PHYS	0.683 ± 0.099	0.772 ± 0.088	0.663 ± 0.123	0.743 ± 0.160	0.406 ± 0.147	0.822 ± 0.107	0.606 ± 0.277	0.850 ± 0.171
DOD-H	0.798 ± 0.077	0.865 ± 0.052	0.801 ± 0.076	0.873 ± 0.134	0.545 ± 0.171	0.867 ± 0.064	0.780 ± 0.174	0.922 ± 0.068
DOD-O	0.775 ± 0.077	0.865 ± 0.060	0.792 ± 0.089	0.918 ± 0.053	0.535 ± 0.132	0.876 ± 0.072	0.636 ± 0.277	0.912 ± 0.139
BSWR	0.711 ± 0.109	0.787 ± 0.099	0.686 ± 0.139	0.827 ± 0.141	0.440 ± 0.161	0.802 ± 0.126	0.627 ± 0.289	0.864 ± 0.169
**Avg ID**	0.764 ± 0.045	0.861 ± 0.035	0.791 ± 0.053	0.907 ± 0.056	0.475 ± 0.081	0.858 ± 0.026	0.681 ± 0.161	0.885 ± 0.024
**Avg OOD**	0.749 ± 0.044	0.851 ± 0.059	0.767 ± 0.072	0.871 ± 0.094	0.466 ± 0.061	0.843 ± 0.027	0.679 ± 0.080	0.884 ± 0.028

Supplementary Table 9 DeepResNet$_{\mathbf{EEG+EOG}}$ benchmarking. MF1, Accuracy and Cohen's κ, plus Class-Wise F1 score on recording-level for DeepResNet using both EEG and EOG channel derivations.

**ID Dataset**	**MF1**	**Acc**	κ	**W**	**N1**	**N2**	**N3**	**REM**
ABC	0.764 ± 0.096	0.834 ± 0.053	0.756 ± 0.065	0.879 ± 0.050	0.569 ± 0.097	0.849 ± 0.082	0.616 ± 0.292	0.884 ± 0.199
CCSHS	0.856 ± 0.050	0.918 ± 0.039	0.884 ± 0.054	0.969 ± 0.019	0.600 ± 0.142	0.910 ± 0.050	0.875 ± 0.089	0.925 ± 0.045
CFS	0.804 ± 0.085	0.901 ± 0.061	0.849 ± 0.098	0.960 ± 0.039	0.501 ± 0.179	0.883 ± 0.096	0.775 ± 0.222	0.901 ± 0.115
CHAT	0.820 ± 0.048	0.877 ± 0.047	0.833 ± 0.063	0.950 ± 0.040	0.528 ± 0.129	0.847 ± 0.080	0.896 ± 0.071	0.880 ± 0.065
HOMEPAP	0.726 ± 0.138	0.803 ± 0.130	0.715 ± 0.182	0.885 ± 0.119	0.382 ± 0.157	0.785 ± 0.165	0.731 ± 0.275	0.875 ± 0.191
MESA	0.733 ± 0.087	0.861 ± 0.077	0.790 ± 0.113	0.942 ± 0.086	0.487 ± 0.135	0.841 ± 0.103	0.500 ± 0.309	0.889 ± 0.078
MROS	0.736 ± 0.084	0.889 ± 0.042	0.822 ± 0.068	0.951 ± 0.036	0.403 ± 0.148	0.870 ± 0.060	0.579 ± 0.278	0.870 ± 0.144
SHHS	0.763 ± 0.091	0.871 ± 0.067	0.810 ± 0.098	0.930 ± 0.073	0.421 ± 0.203	0.864 ± 0.079	0.709 ± 0.232	0.896 ± 0.085
SOF	0.747 ± 0.074	0.876 ± 0.058	0.806 ± 0.092	0.946 ± 0.047	0.353 ± 0.161	0.844 ± 0.079	0.694 ± 0.190	0.904 ± 0.083
APOE	0.709 ± 0.102	0.805 ± 0.086	0.702 ± 0.125	0.866 ± 0.082	0.416 ± 0.165	0.838 ± 0.102	0.542 ± 0.312	0.858 ± 0.146
APPLES	0.731 ± 0.091	0.821 ± 0.091	0.733 ± 0.118	0.905 ± 0.056	0.476 ± 0.153	0.832 ± 0.144	0.391 ± 0.297	0.875 ± 0.144
MNC-SSC	0.673 ± 0.103	0.797 ± 0.082	0.685 ± 0.123	0.801 ± 0.126	0.298 ± 0.161	0.842 ± 0.079	0.565 ± 0.300	0.852 ± 0.163
MNC-DHC	0.797 ± 0.063	0.905 ± 0.045	0.845 ± 0.073	0.973 ± 0.023	0.470 ± 0.150	0.842 ± 0.072	0.814 ± 0.105	0.883 ± 0.065
MNC-CNC	0.752 ± 0.057	0.811 ± 0.048	0.741 ± 0.058	0.773 ± 0.162	0.465 ± 0.123	0.820 ± 0.095	0.842 ± 0.060	0.861 ± 0.061
MSP	0.768 ± 0.077	0.873 ± 0.029	0.797 ± 0.055	0.912 ± 0.066	0.488 ± 0.111	0.880 ± 0.056	0.655 ± 0.262	0.878 ± 0.122
WSC	0.729 ± 0.099	0.854 ± 0.072	0.766 ± 0.112	0.883 ± 0.108	0.467 ± 0.163	0.896 ± 0.058	0.495 ± 0.279	0.878 ± 0.142
NCHSDB	0.739 ± 0.076	0.856 ± 0.055	0.785 ± 0.107	0.840 ± 0.109	0.297 ± 0.156	0.842 ± 0.120	0.890 ± 0.097	0.830 ± 0.157
**OOD Dataset**	**MF1**	**Acc**	κ	**W**	**N1**	**N2**	**N3**	**REM**
DCSM	0.784 ± 0.082	0.915 ± 0.046	0.846 ± 0.082	0.977 ± 0.025	0.468 ± 0.152	0.831 ± 0.105	0.759 ± 0.212	0.890 ± 0.126
SEDF-ST	0.754 ± 0.071	0.831 ± 0.050	0.753 ± 0.072	0.790 ± 0.110	0.483 ± 0.145	0.863 ± 0.057	0.743 ± 0.228	0.893 ± 0.076
SEDF-SC	0.722 ± 0.083	0.903 ± 0.045	0.804 ± 0.082	0.971 ± 0.025	0.391 ± 0.133	0.824 ± 0.081	0.599 ± 0.281	0.809 ± 0.145
PHYS	0.688 ± 0.098	0.777 ± 0.089	0.671 ± 0.124	0.739 ± 0.161	0.379 ± 0.156	0.830 ± 0.105	0.673 ± 0.257	0.848 ± 0.169
DOD-H	0.801 ± 0.081	0.880 ± 0.041	0.818 ± 0.071	0.867 ± 0.144	0.517 ± 0.155	0.890 ± 0.041	0.825 ± 0.158	0.902 ± 0.140
DOD-O	0.779 ± 0.081	0.872 ± 0.056	0.800 ± 0.087	0.918 ± 0.055	0.531 ± 0.132	0.887 ± 0.058	0.646 ± 0.283	0.915 ± 0.130
BSWR	0.705 ± 0.111	0.785 ± 0.101	0.681 ± 0.140	0.821 ± 0.143	0.404 ± 0.159	0.804 ± 0.127	0.643 ± 0.281	0.861 ± 0.165
**Avg ID**	0.756 ± 0.044	0.856 ± 0.038	0.783 ± 0.056	0.904 ± 0.059	0.448 ± 0.085	0.852 ± 0.030	0.681 ± 0.155	0.879 ± 0.022
**Avg OOD**	0.748 ± 0.043	0.852 ± 0.055	0.768 ± 0.068	0.869 ± 0.091	0.453 ± 0.062	0.847 ± 0.033	0.696 ± 0.081	0.874 ± 0.037

Supplementary Table 10 SleepTransformer$_{\mathbf{EEG+EOG}}$ benchmarking. MF1, Accuracy and Cohen's κ, plus Class-Wise F1 score on recording-level for SleepTransformer using both EEG and EOG channel derivations.

**ID Dataset**	**MF1**	**Acc**	κ	**W**	**N1**	**N2**	**N3**	**REM**
ABC	0.790 ± 0.084	0.851 ± 0.042	0.780 ± 0.048	0.892 ± 0.047	0.606 ± 0.090	0.862 ± 0.066	0.683 ± 0.272	0.891 ± 0.200
CCSHS	0.866 ± 0.049	0.923 ± 0.033	0.893 ± 0.047	0.973 ± 0.016	0.629 ± 0.148	0.915 ± 0.047	0.876 ± 0.097	0.935 ± 0.038
CFS	0.811 ± 0.081	0.901 ± 0.062	0.850 ± 0.101	0.962 ± 0.039	0.542 ± 0.154	0.880 ± 0.101	0.764 ± 0.228	0.910 ± 0.085
CHAT	0.848 ± 0.039	0.889 ± 0.042	0.850 ± 0.057	0.963 ± 0.030	0.622 ± 0.097	0.855 ± 0.076	0.897 ± 0.071	0.903 ± 0.054
HOMEPAP	0.760 ± 0.082	0.833 ± 0.079	0.750 ± 0.128	0.910 ± 0.073	0.452 ± 0.143	0.824 ± 0.097	0.734 ± 0.234	0.900 ± 0.119
MESA	0.751 ± 0.091	0.873 ± 0.069	0.807 ± 0.110	0.953 ± 0.061	0.509 ± 0.142	0.850 ± 0.105	0.540 ± 0.297	0.901 ± 0.079
MROS	0.751 ± 0.086	0.896 ± 0.037	0.835 ± 0.060	0.957 ± 0.036	0.464 ± 0.162	0.877 ± 0.054	0.570 ± 0.289	0.875 ± 0.150
SHHS	0.777 ± 0.095	0.878 ± 0.067	0.820 ± 0.105	0.932 ± 0.083	0.454 ± 0.210	0.869 ± 0.081	0.742 ± 0.223	0.899 ± 0.095
SOF	0.770 ± 0.075	0.884 ± 0.059	0.819 ± 0.094	0.948 ± 0.051	0.423 ± 0.164	0.851 ± 0.078	0.711 ± 0.209	0.925 ± 0.067
APOE	0.729 ± 0.097	0.826 ± 0.078	0.732 ± 0.117	0.873 ± 0.076	0.442 ± 0.167	0.859 ± 0.092	0.578 ± 0.330	0.870 ± 0.123
APPLES	0.740 ± 0.096	0.839 ± 0.067	0.757 ± 0.094	0.912 ± 0.053	0.529 ± 0.152	0.849 ± 0.131	0.306 ± 0.322	0.871 ± 0.152
MNC-SSC	0.692 ± 0.104	0.819 ± 0.073	0.717 ± 0.115	0.813 ± 0.124	0.320 ± 0.170	0.867 ± 0.063	0.598 ± 0.307	0.855 ± 0.166
MNC-DHC	0.811 ± 0.057	0.912 ± 0.042	0.857 ± 0.067	0.978 ± 0.014	0.513 ± 0.135	0.847 ± 0.093	0.822 ± 0.087	0.894 ± 0.055
MNC-CNC	0.783 ± 0.066	0.833 ± 0.060	0.772 ± 0.072	0.805 ± 0.151	0.550 ± 0.124	0.827 ± 0.106	0.855 ± 0.074	0.879 ± 0.063
MSP	0.778 ± 0.078	0.875 ± 0.036	0.803 ± 0.061	0.927 ± 0.061	0.502 ± 0.088	0.883 ± 0.052	0.664 ± 0.275	0.887 ± 0.101
WSC	0.743 ± 0.098	0.860 ± 0.070	0.777 ± 0.110	0.898 ± 0.102	0.519 ± 0.153	0.899 ± 0.056	0.489 ± 0.283	0.886 ± 0.140
NCHSDB	0.753 ± 0.077	0.868 ± 0.044	0.801 ± 0.096	0.853 ± 0.119	0.329 ± 0.165	0.850 ± 0.129	0.895 ± 0.076	0.845 ± 0.146
**OOD Dataset**	**MF1**	**Acc**	κ	**W**	**N1**	**N2**	**N3**	**REM**
DCSM	0.800 ± 0.083	0.927 ± 0.044	0.866 ± 0.079	0.982 ± 0.024	0.474 ± 0.154	0.857 ± 0.095	0.803 ± 0.189	0.890 ± 0.147
SEDF-ST	0.747 ± 0.080	0.833 ± 0.051	0.752 ± 0.076	0.789 ± 0.112	0.469 ± 0.152	0.868 ± 0.055	0.711 ± 0.251	0.898 ± 0.084
SEDF-SC	0.719 ± 0.088	0.913 ± 0.040	0.818 ± 0.076	0.982 ± 0.013	0.346 ± 0.147	0.813 ± 0.098	0.559 ± 0.288	0.874 ± 0.092
PHYS	0.691 ± 0.098	0.777 ± 0.089	0.673 ± 0.126	0.740 ± 0.162	0.379 ± 0.156	0.830 ± 0.105	0.673 ± 0.257	0.848 ± 0.169
DOD-H	0.833 ± 0.062	0.901 ± 0.034	0.852 ± 0.057	0.885 ± 0.086	0.570 ± 0.158	0.914 ± 0.038	0.858 ± 0.162	0.940 ± 0.047
DOD-O	0.721 ± 0.099	0.810 ± 0.085	0.710 ± 0.117	0.822 ± 0.126	0.469 ± 0.153	0.849 ± 0.078	0.699 ± 0.267	0.770 ± 0.178
BSWR	0.710 ± 0.116	0.790 ± 0.106	0.692 ± 0.147	0.827 ± 0.144	0.380 ± 0.181	0.806 ± 0.129	0.684 ± 0.289	0.862 ± 0.172
**Avg ID**	0.774 ± 0.043	0.868 ± 0.031	0.801 ± 0.048	0.915 ± 0.053	0.494 ± 0.088	0.863 ± 0.023	0.690 ± 0.162	0.890 ± 0.023
**Avg OOD**	0.746 ± 0.052	0.850 ± 0.062	0.766 ± 0.079	0.861 ± 0.093	0.441 ± 0.077	0.848 ± 0.037	0.712 ± 0.096	0.869 ± 0.053

Supplementary Table 11 **SOMNUS$_{\mathbf{EEG+EOG}}$ benchmarking.** MF1, Accuracy and Cohen's κ, plus Class-Wise F1 score on recording-level for soft unweighted voting ensemble considering all models using both EEG and EOG channel derivations.

**ID Dataset**	**MF1**	**Acc**	κ	**W**	**N1**	**N2**	**N3**	**REM**
ABC	0.785 ± 0.088	0.850 ± 0.046	0.778 ± 0.055	0.890 ± 0.048	0.596 ± 0.085	0.864 ± 0.067	0.667 ± 0.277	0.893 ± 0.200
CCSHS	0.871 ± 0.047	0.925 ± 0.035	0.895 ± 0.049	0.972 ± 0.017	0.650 ± 0.139	0.918 ± 0.047	0.880 ± 0.091	0.936 ± 0.037
CFS	0.818 ± 0.079	0.907 ± 0.059	0.858 ± 0.097	0.964 ± 0.038	0.539 ± 0.157	0.889 ± 0.096	0.787 ± 0.207	0.913 ± 0.089
CHAT	0.838 ± 0.045	0.887 ± 0.045	0.847 ± 0.061	0.960 ± 0.031	0.572 ± 0.122	0.857 ± 0.076	0.899 ± 0.071	0.901 ± 0.057
HOMEPAP	0.745 ± 0.102	0.826 ± 0.089	0.742 ± 0.141	0.899 ± 0.087	0.413 ± 0.156	0.822 ± 0.095	0.730 ± 0.271	0.883 ± 0.167
MESA	0.749 ± 0.088	0.871 ± 0.072	0.805 ± 0.113	0.949 ± 0.073	0.503 ± 0.138	0.850 ± 0.105	0.539 ± 0.300	0.903 ± 0.072
MROS	0.755 ± 0.083	0.898 ± 0.039	0.836 ± 0.063	0.957 ± 0.035	0.452 ± 0.156	0.879 ± 0.059	0.597 ± 0.275	0.881 ± 0.143
SHHS	0.779 ± 0.089	0.879 ± 0.064	0.822 ± 0.094	0.936 ± 0.072	0.464 ± 0.205	0.871 ± 0.078	0.729 ± 0.220	0.903 ± 0.088
SOF	0.773 ± 0.072	0.887 ± 0.057	0.822 ± 0.092	0.950 ± 0.047	0.414 ± 0.156	0.855 ± 0.078	0.724 ± 0.194	0.928 ± 0.067
APOE	0.724 ± 0.102	0.822 ± 0.082	0.726 ± 0.122	0.872 ± 0.082	0.437 ± 0.168	0.854 ± 0.099	0.560 ± 0.323	0.869 ± 0.131
APPLES	0.743 ± 0.089	0.834 ± 0.079	0.750 ± 0.107	0.912 ± 0.052	0.510 ± 0.151	0.844 ± 0.135	0.387 ± 0.327	0.873 ± 0.151
MNC-SSC	0.686 ± 0.106	0.814 ± 0.076	0.708 ± 0.119	0.809 ± 0.125	0.314 ± 0.173	0.861 ± 0.068	0.580 ± 0.307	0.857 ± 0.166
MNC-DHC	0.814 ± 0.062	0.916 ± 0.040	0.863 ± 0.064	0.978 ± 0.015	0.506 ± 0.158	0.858 ± 0.077	0.829 ± 0.100	0.899 ± 0.053
MNC-CNC	0.780 ± 0.062	0.836 ± 0.050	0.775 ± 0.061	0.796 ± 0.161	0.521 ± 0.130	0.836 ± 0.101	0.860 ± 0.064	0.887 ± 0.043
MSP	0.784 ± 0.074	0.881 ± 0.032	0.809 ± 0.057	0.923 ± 0.057	0.514 ± 0.104	0.889 ± 0.055	0.675 ± 0.268	0.895 ± 0.108
WSC	0.740 ± 0.099	0.865 ± 0.071	0.783 ± 0.110	0.894 ± 0.107	0.515 ± 0.163	0.906 ± 0.057	0.470 ± 0.293	0.887 ± 0.138
NCHSDB	0.756 ± 0.071	0.872 ± 0.043	0.805 ± 0.092	0.860 ± 0.104	0.329 ± 0.165	0.852 ± 0.127	0.895 ± 0.094	0.848 ± 0.153
**OOD Dataset**	**MF1**	**Acc**	κ	**W**	**N1**	**N2**	**N3**	**REM**
DCSM	0.803 ± 0.080	0.928 ± 0.043	0.868 ± 0.076	0.983 ± 0.023	0.488 ± 0.149	0.859 ± 0.094	0.793 ± 0.194	0.897 ± 0.139
SEDF-ST	0.753 ± 0.077	0.835 ± 0.051	0.757 ± 0.075	0.790 ± 0.113	0.466 ± 0.152	0.868 ± 0.056	0.739 ± 0.241	0.900 ± 0.082
SEDF-SC	0.738 ± 0.085	0.918 ± 0.036	0.830 ± 0.070	0.983 ± 0.012	0.387 ± 0.140	0.827 ± 0.084	0.597 ± 0.292	0.876 ± 0.112
PHYS	0.694 ± 0.097	0.782 ± 0.086	0.677 ± 0.122	0.745 ± 0.160	0.395 ± 0.154	0.834 ± 0.103	0.650 ± 0.269	0.857 ± 0.164
DOD-H	0.827 ± 0.074	0.898 ± 0.039	0.847 ± 0.062	0.881 ± 0.133	0.557 ± 0.175	0.906 ± 0.043	0.843 ± 0.160	0.943 ± 0.053
DOD-O	0.789 ± 0.082	0.873 ± 0.062	0.804 ± 0.094	0.922 ± 0.055	0.537 ± 0.131	0.886 ± 0.063	0.687 ± 0.279	0.919 ± 0.126
BSWR	0.717 ± 0.111	0.795 ± 0.101	0.697 ± 0.141	0.830 ± 0.141	0.405 ± 0.168	0.812 ± 0.123	0.675 ± 0.279	0.872 ± 0.162
**Avg ID**	0.773 ± 0.045	0.869 ± 0.034	0.801 ± 0.051	0.913 ± 0.055	0.485 ± 0.087	0.865 ± 0.025	0.695 ± 0.155	0.892 ± 0.023
**Avg OOD**	0.760 ± 0.048	0.861 ± 0.058	0.783 ± 0.074	0.876 ± 0.093	0.462 ± 0.069	0.856 ± 0.034	0.712 ± 0.085	0.895 ± 0.030

Supplementary Table 12 U-Sleep$_{\mathbf{EEG}}$ benchmarking. MF1, Accuracy and Cohen's κ, plus Class-Wise F1 score on recording-level for U-Sleep using EEG channel derivations.

**ID Dataset**	**MF1**	**Acc**	κ	**W**	**N1**	**N2**	**N3**	**REM**
ABC	0.759 ± 0.093	0.838 ± 0.051	0.758 ± 0.060	0.882 ± 0.048	0.566 ± 0.095	0.853 ± 0.076	0.597 ± 0.323	0.878 ± 0.201
CCSHS	0.855 ± 0.060	0.916 ± 0.048	0.882 ± 0.066	0.964 ± 0.039	0.607 ± 0.154	0.910 ± 0.057	0.869 ± 0.108	0.927 ± 0.049
CFS	0.795 ± 0.091	0.893 ± 0.063	0.838 ± 0.103	0.959 ± 0.040	0.527 ± 0.161	0.875 ± 0.101	0.729 ± 0.229	0.883 ± 0.137
CHAT	0.820 ± 0.047	0.873 ± 0.050	0.828 ± 0.067	0.946 ± 0.040	0.545 ± 0.120	0.845 ± 0.087	0.883 ± 0.076	0.883 ± 0.067
HOMEPAP	0.742 ± 0.070	0.826 ± 0.082	0.742 ± 0.130	0.897 ± 0.084	0.375 ± 0.132	0.820 ± 0.095	0.757 ± 0.227	0.891 ± 0.120
MESA	0.718 ± 0.098	0.856 ± 0.078	0.780 ± 0.122	0.936 ± 0.092	0.453 ± 0.153	0.841 ± 0.101	0.483 ± 0.275	0.868 ± 0.123
MROS	0.738 ± 0.086	0.887 ± 0.047	0.821 ± 0.073	0.954 ± 0.041	0.448 ± 0.161	0.869 ± 0.062	0.558 ± 0.277	0.856 ± 0.165
SHHS	0.752 ± 0.093	0.860 ± 0.072	0.793 ± 0.103	0.930 ± 0.071	0.442 ± 0.208	0.855 ± 0.086	0.653 ± 0.239	0.882 ± 0.109
SOF	0.745 ± 0.077	0.868 ± 0.070	0.794 ± 0.108	0.950 ± 0.048	0.428 ± 0.152	0.834 ± 0.095	0.638 ± 0.211	0.887 ± 0.125
APOE	0.697 ± 0.104	0.793 ± 0.093	0.685 ± 0.134	0.866 ± 0.082	0.384 ± 0.171	0.826 ± 0.110	0.548 ± 0.310	0.840 ± 0.144
APPLES	0.721 ± 0.091	0.807 ± 0.089	0.712 ± 0.118	0.907 ± 0.052	0.449 ± 0.152	0.814 ± 0.146	0.423 ± 0.324	0.857 ± 0.159
MNC-SSC	0.665 ± 0.105	0.795 ± 0.080	0.680 ± 0.123	0.795 ± 0.128	0.285 ± 0.169	0.844 ± 0.072	0.558 ± 0.300	0.832 ± 0.171
MNC-DHC	0.794 ± 0.060	0.904 ± 0.043	0.844 ± 0.068	0.976 ± 0.016	0.505 ± 0.130	0.834 ± 0.095	0.770 ± 0.113	0.887 ± 0.050
MNC-CNC	0.739 ± 0.066	0.801 ± 0.060	0.725 ± 0.075	0.773 ± 0.172	0.451 ± 0.116	0.808 ± 0.118	0.818 ± 0.078	0.847 ± 0.053
MSP	0.767 ± 0.074	0.870 ± 0.040	0.793 ± 0.064	0.919 ± 0.070	0.495 ± 0.071	0.880 ± 0.057	0.659 ± 0.259	0.861 ± 0.130
WSC	0.713 ± 0.098	0.843 ± 0.074	0.749 ± 0.115	0.878 ± 0.110	0.433 ± 0.167	0.889 ± 0.063	0.483 ± 0.280	0.856 ± 0.140
NCHSDB	0.739 ± 0.082	0.853 ± 0.063	0.783 ± 0.109	0.844 ± 0.101	0.306 ± 0.165	0.848 ± 0.119	0.884 ± 0.101	0.822 ± 0.170
**OOD Dataset**	**MF1**	**Acc**	κ	**W**	**N1**	**N2**	**N3**	**REM**
DCSM	0.783 ± 0.086	0.920 ± 0.046	0.854 ± 0.080	0.981 ± 0.022	0.480 ± 0.148	0.846 ± 0.102	0.736 ± 0.230	0.873 ± 0.143
SEDF-ST	0.758 ± 0.075	0.839 ± 0.054	0.761 ± 0.076	0.810 ± 0.110	0.475 ± 0.151	0.870 ± 0.061	0.740 ± 0.234	0.895 ± 0.082
SEDF-SC	0.720 ± 0.090	0.912 ± 0.040	0.817 ± 0.081	0.981 ± 0.014	0.342 ± 0.131	0.814 ± 0.097	0.602 ± 0.288	0.845 ± 0.115
PHYS	0.687 ± 0.101	0.776 ± 0.096	0.671 ± 0.133	0.746 ± 0.162	0.342 ± 0.155	0.828 ± 0.112	0.698 ± 0.240	0.835 ± 0.173
DOD-H	0.816 ± 0.074	0.890 ± 0.051	0.834 ± 0.081	0.878 ± 0.087	0.526 ± 0.169	0.907 ± 0.052	0.851 ± 0.175	0.916 ± 0.075
DOD-O	0.775 ± 0.084	0.865 ± 0.071	0.792 ± 0.102	0.906 ± 0.078	0.495 ± 0.147	0.881 ± 0.071	0.696 ± 0.269	0.904 ± 0.101
BSWR	0.700 ± 0.112	0.781 ± 0.103	0.676 ± 0.144	0.828 ± 0.141	0.384 ± 0.165	0.798 ± 0.129	0.652 ± 0.265	0.847 ± 0.179
**Avg ID**	0.751 ± 0.046	0.852 ± 0.038	0.777 ± 0.057	0.904 ± 0.059	0.453 ± 0.085	0.850 ± 0.028	0.665 ± 0.148	0.868 ± 0.026
**Avg OOD**	0.748 ± 0.047	0.855 ± 0.059	0.772 ± 0.074	0.876 ± 0.088	0.435 ± 0.077	0.849 ± 0.039	0.711 ± 0.078	0.874 ± 0.032

Supplementary Table 13 DeepResNet$_{\mathbf{EEG}}$ benchmarking. MF1, Accuracy and Cohen's κ, plus Class-Wise F1 score on recording-level for DeepResNet using EEG channel derivations.

**ID Dataset**	**MF1**	**Acc**	κ	**W**	**N1**	**N2**	**N3**	**REM**
ABC	0.785 ± 0.095	0.846 ± 0.049	0.773 ± 0.061	0.890 ± 0.047	0.615 ± 0.103	0.858 ± 0.071	0.667 ± 0.261	0.882 ± 0.200
CCSHS	0.857 ± 0.051	0.920 ± 0.038	0.887 ± 0.052	0.969 ± 0.023	0.599 ± 0.148	0.914 ± 0.049	0.877 ± 0.090	0.926 ± 0.044
CFS	0.802 ± 0.088	0.898 ± 0.063	0.845 ± 0.101	0.959 ± 0.041	0.516 ± 0.159	0.881 ± 0.098	0.759 ± 0.239	0.893 ± 0.120
CHAT	0.827 ± 0.044	0.878 ± 0.047	0.834 ± 0.063	0.952 ± 0.036	0.556 ± 0.116	0.849 ± 0.078	0.892 ± 0.073	0.884 ± 0.064
HOMEPAP	0.754 ± 0.085	0.829 ± 0.082	0.748 ± 0.133	0.904 ± 0.079	0.421 ± 0.144	0.817 ± 0.109	0.756 ± 0.240	0.902 ± 0.078
MESA	0.731 ± 0.091	0.860 ± 0.074	0.788 ± 0.112	0.939 ± 0.087	0.483 ± 0.148	0.844 ± 0.099	0.500 ± 0.298	0.880 ± 0.090
MROS	0.738 ± 0.085	0.888 ± 0.046	0.821 ± 0.074	0.953 ± 0.039	0.422 ± 0.153	0.870 ± 0.063	0.581 ± 0.275	0.855 ± 0.167
SHHS	0.762 ± 0.095	0.868 ± 0.068	0.805 ± 0.101	0.928 ± 0.076	0.443 ± 0.208	0.863 ± 0.080	0.699 ± 0.232	0.883 ± 0.119
SOF	0.752 ± 0.078	0.876 ± 0.066	0.806 ± 0.104	0.949 ± 0.052	0.403 ± 0.146	0.847 ± 0.080	0.675 ± 0.208	0.897 ± 0.096
APOE	0.701 ± 0.105	0.797 ± 0.093	0.690 ± 0.134	0.865 ± 0.083	0.396 ± 0.172	0.830 ± 0.110	0.555 ± 0.311	0.838 ± 0.155
APPLES	0.720 ± 0.098	0.810 ± 0.101	0.719 ± 0.129	0.908 ± 0.052	0.451 ± 0.156	0.822 ± 0.148	0.382 ± 0.312	0.862 ± 0.155
MNC-SSC	0.670 ± 0.104	0.798 ± 0.079	0.683 ± 0.122	0.797 ± 0.125	0.290 ± 0.166	0.846 ± 0.073	0.571 ± 0.297	0.837 ± 0.166
MNC-DHC	0.799 ± 0.066	0.905 ± 0.045	0.846 ± 0.071	0.975 ± 0.019	0.490 ± 0.151	0.839 ± 0.088	0.814 ± 0.097	0.878 ± 0.065
MNC-CNC	0.755 ± 0.068	0.813 ± 0.060	0.743 ± 0.075	0.776 ± 0.169	0.477 ± 0.132	0.819 ± 0.111	0.837 ± 0.073	0.866 ± 0.063
MSP	0.778 ± 0.071	0.877 ± 0.038	0.804 ± 0.061	0.927 ± 0.059	0.513 ± 0.087	0.887 ± 0.048	0.665 ± 0.265	0.880 ± 0.121
WSC	0.727 ± 0.098	0.851 ± 0.073	0.762 ± 0.113	0.885 ± 0.109	0.469 ± 0.163	0.894 ± 0.059	0.497 ± 0.275	0.865 ± 0.141
NCHSDB	0.741 ± 0.085	0.858 ± 0.069	0.789 ± 0.119	0.846 ± 0.113	0.300 ± 0.169	0.847 ± 0.129	0.893 ± 0.098	0.825 ± 0.168
**OOD Dataset**	**MF1**	**Acc**	κ	**W**	**N1**	**N2**	**N3**	**REM**
DCSM	0.797 ± 0.086	0.923 ± 0.047	0.859 ± 0.082	0.981 ± 0.027	0.507 ± 0.148	0.849 ± 0.096	0.779 ± 0.207	0.874 ± 0.149
SEDF-ST	0.764 ± 0.075	0.835 ± 0.055	0.758 ± 0.079	0.814 ± 0.106	0.508 ± 0.160	0.863 ± 0.063	0.746 ± 0.235	0.895 ± 0.082
SEDF-SC	0.711 ± 0.088	0.898 ± 0.052	0.794 ± 0.091	0.969 ± 0.033	0.349 ± 0.143	0.800 ± 0.096	0.599 ± 0.278	0.822 ± 0.125
PHYS	0.687 ± 0.097	0.777 ± 0.091	0.672 ± 0.126	0.744 ± 0.159	0.358 ± 0.153	0.832 ± 0.106	0.682 ± 0.247	0.837 ± 0.173
DOD-H	0.806 ± 0.068	0.886 ± 0.034	0.825 ± 0.058	0.875 ± 0.099	0.536 ± 0.161	0.905 ± 0.036	0.813 ± 0.192	0.901 ± 0.084
DOD-O	0.763 ± 0.084	0.853 ± 0.075	0.771 ± 0.112	0.902 ± 0.085	0.474 ± 0.144	0.870 ± 0.077	0.702 ± 0.272	0.874 ± 0.107
BSWR	0.702 ± 0.112	0.781 ± 0.103	0.675 ± 0.144	0.828 ± 0.141	0.384 ± 0.165	0.798 ± 0.127	0.652 ± 0.265	0.847 ± 0.179
**Avg ID**	0.759 ± 0.046	0.857 ± 0.037	0.785 ± 0.056	0.907 ± 0.058	0.461 ± 0.089	0.855 ± 0.027	0.684 ± 0.153	0.874 ± 0.026
**Avg OOD**	0.747 ± 0.047	0.850 ± 0.057	0.765 ± 0.071	0.873 ± 0.088	0.435 ± 0.077	0.849 ± 0.039	0.711 ± 0.078	0.864 ± 0.029

Supplementary Table 14 SleepTransformer$_{\mathbf{EEG}}$ benchmarking. MF1, Accuracy and Cohen's κ, plus Class-Wise F1 score on recording-level for SleepTransformer using EEG channel derivations.

**ID Dataset**	**MF1**	**Acc**	κ	**W**	**N1**	**N2**	**N3**	**REM**
ABC	0.789 ± 0.065	0.846 ± 0.046	0.772 ± 0.054	0.893 ± 0.049	0.594 ± 0.097	0.858 ± 0.067	0.682 ± 0.246	0.904 ± 0.119
CCSHS	0.861 ± 0.049	0.919 ± 0.036	0.886 ± 0.050	0.971 ± 0.019	0.623 ± 0.143	0.911 ± 0.049	0.875 ± 0.089	0.925 ± 0.045
CFS	0.803 ± 0.081	0.897 ± 0.061	0.843 ± 0.100	0.958 ± 0.041	0.532 ± 0.153	0.878 ± 0.094	0.749 ± 0.227	0.902 ± 0.091
CHAT	0.838 ± 0.042	0.883 ± 0.044	0.841 ± 0.059	0.961 ± 0.029	0.594 ± 0.108	0.850 ± 0.074	0.892 ± 0.071	0.895 ± 0.059
HOMEPAP	0.744 ± 0.085	0.820 ± 0.084	0.734 ± 0.136	0.905 ± 0.081	0.412 ± 0.136	0.806 ± 0.117	0.757 ± 0.241	0.869 ± 0.130
MESA	0.744 ± 0.076	0.872 ± 0.055	0.806 ± 0.084	0.953 ± 0.052	0.488 ± 0.139	0.857 ± 0.076	0.532 ± 0.290	0.885 ± 0.088
MROS	0.743 ± 0.083	0.890 ± 0.042	0.825 ± 0.069	0.954 ± 0.039	0.448 ± 0.160	0.870 ± 0.064	0.570 ± 0.278	0.864 ± 0.150
SHHS	0.764 ± 0.092	0.875 ± 0.060	0.815 ± 0.092	0.931 ± 0.078	0.417 ± 0.209	0.866 ± 0.073	0.726 ± 0.226	0.884 ± 0.120
SOF	0.762 ± 0.074	0.883 ± 0.058	0.816 ± 0.089	0.951 ± 0.044	0.420 ± 0.165	0.850 ± 0.077	0.687 ± 0.217	0.907 ± 0.079
APOE	0.703 ± 0.100	0.801 ± 0.089	0.695 ± 0.133	0.869 ± 0.081	0.391 ± 0.156	0.834 ± 0.100	0.559 ± 0.318	0.838 ± 0.162
APPLES	0.728 ± 0.093	0.827 ± 0.082	0.740 ± 0.107	0.910 ± 0.052	0.496 ± 0.152	0.841 ± 0.132	0.310 ± 0.305	0.860 ± 0.151
MNC-SSC	0.670 ± 0.103	0.797 ± 0.081	0.685 ± 0.122	0.809 ± 0.120	0.278 ± 0.154	0.842 ± 0.080	0.571 ± 0.297	0.840 ± 0.168
MNC-DHC	0.803 ± 0.057	0.908 ± 0.043	0.851 ± 0.069	0.977 ± 0.017	0.488 ± 0.134	0.847 ± 0.085	0.826 ± 0.085	0.876 ± 0.070
MNC-CNC	0.772 ± 0.066	0.822 ± 0.065	0.754 ± 0.083	0.815 ± 0.147	0.537 ± 0.105	0.813 ± 0.112	0.844 ± 0.084	0.852 ± 0.065
MSP	0.759 ± 0.069	0.870 ± 0.033	0.795 ± 0.052	0.927 ± 0.066	0.501 ± 0.076	0.879 ± 0.049	0.573 ± 0.278	0.889 ± 0.098
WSC	0.727 ± 0.095	0.840 ± 0.073	0.748 ± 0.114	0.895 ± 0.100	0.461 ± 0.152	0.879 ± 0.061	0.510 ± 0.272	0.867 ± 0.135
NCHSDB	0.742 ± 0.084	0.864 ± 0.050	0.795 ± 0.108	0.842 ± 0.127	0.299 ± 0.179	0.847 ± 0.132	0.890 ± 0.098	0.840 ± 0.138
**OOD Dataset**	**MF1**	**Acc**	κ	**W**	**N1**	**N2**	**N3**	**REM**
DCSM	0.793 ± 0.087	0.925 ± 0.044	0.863 ± 0.078	0.982 ± 0.022	0.474 ± 0.153	0.855 ± 0.093	0.789 ± 0.200	0.874 ± 0.158
SEDF-ST	0.751 ± 0.070	0.831 ± 0.052	0.750 ± 0.073	0.799 ± 0.110	0.483 ± 0.149	0.865 ± 0.060	0.723 ± 0.224	0.885 ± 0.087
SEDF-SC	0.706 ± 0.092	0.898 ± 0.052	0.794 ± 0.091	0.969 ± 0.033	0.349 ± 0.143	0.800 ± 0.096	0.599 ± 0.278	0.822 ± 0.125
PHYS	0.674 ± 0.102	0.766 ± 0.099	0.658 ± 0.137	0.729 ± 0.163	0.307 ± 0.150	0.819 ± 0.116	0.702 ± 0.244	0.833 ± 0.178
DOD-H	0.765 ± 0.080	0.833 ± 0.073	0.749 ± 0.110	0.862 ± 0.100	0.475 ± 0.171	0.849 ± 0.084	0.810 ± 0.167	0.831 ± 0.139
DOD-O	0.557 ± 0.126	0.655 ± 0.118	0.473 ± 0.171	0.718 ± 0.193	0.397 ± 0.171	0.710 ± 0.121	0.621 ± 0.257	0.339 ± 0.284
BSWR	0.697 ± 0.120	0.782 ± 0.109	0.679 ± 0.150	0.820 ± 0.148	0.366 ± 0.175	0.800 ± 0.131	0.658 ± 0.308	0.846 ± 0.183
**Avg ID**	0.762 ± 0.047	0.860 ± 0.037	0.788 ± 0.056	0.913 ± 0.053	0.469 ± 0.096	0.855 ± 0.025	0.680 ± 0.163	0.876 ± 0.026
**Avg OOD**	0.706 ± 0.078	0.814 ± 0.091	0.711 ± 0.126	0.841 ± 0.107	0.404 ± 0.074	0.814 ± 0.053	0.696 ± 0.087	0.837 ± 0.176

Supplementary Table 15 SOMNUS$_{\mathbf{EEG}}$ benchmarking. MF1, Accuracy and Cohen's κ, plus Class-Wise F1 score on recording-level for soft unweighted voting ensembling considering all models using EEG channel derivations.

**ID Dataset**	**MF1**	**Acc**	κ	**W**	**N1**	**N2**	**N3**	**REM**
ABC	0.785 ± 0.092	0.852 ± 0.047	0.780 ± 0.056	0.895 ± 0.048	0.602 ± 0.094	0.866 ± 0.066	0.651 ± 0.300	0.892 ± 0.201
CCSHS	0.870 ± 0.050	0.925 ± 0.038	0.895 ± 0.053	0.972 ± 0.019	0.645 ± 0.145	0.918 ± 0.050	0.881 ± 0.092	0.934 ± 0.041
CFS	0.812 ± 0.082	0.904 ± 0.060	0.853 ± 0.098	0.963 ± 0.039	0.545 ± 0.164	0.886 ± 0.096	0.760 ± 0.222	0.906 ± 0.094
CHAT	0.838 ± 0.042	0.885 ± 0.045	0.844 ± 0.061	0.958 ± 0.032	0.584 ± 0.114	0.856 ± 0.078	0.893 ± 0.072	0.898 ± 0.059
HOMEPAP	0.758 ± 0.079	0.833 ± 0.083	0.752 ± 0.133	0.907 ± 0.082	0.410 ± 0.141	0.821 ± 0.104	0.784 ± 0.217	0.900 ± 0.116
MESA	0.741 ± 0.084	0.871 ± 0.066	0.803 ± 0.098	0.947 ± 0.074	0.486 ± 0.152	0.857 ± 0.087	0.517 ± 0.291	0.894 ± 0.080
MROS	0.749 ± 0.084	0.896 ± 0.041	0.834 ± 0.065	0.957 ± 0.037	0.456 ± 0.167	0.878 ± 0.057	0.575 ± 0.285	0.868 ± 0.155
SHHS	0.773 ± 0.092	0.878 ± 0.064	0.819 ± 0.094	0.935 ± 0.070	0.449 ± 0.216	0.872 ± 0.076	0.720 ± 0.219	0.893 ± 0.116
SOF	0.769 ± 0.074	0.885 ± 0.060	0.819 ± 0.094	0.953 ± 0.045	0.441 ± 0.160	0.854 ± 0.081	0.687 ± 0.202	0.913 ± 0.085
APOE	0.707 ± 0.104	0.803 ± 0.092	0.699 ± 0.135	0.873 ± 0.081	0.394 ± 0.169	0.834 ± 0.108	0.569 ± 0.313	0.846 ± 0.155
APPLES	0.729 ± 0.094	0.822 ± 0.090	0.733 ± 0.118	0.912 ± 0.050	0.471 ± 0.159	0.833 ± 0.140	0.384 ± 0.322	0.864 ± 0.158
MNC-SSC	0.674 ± 0.106	0.803 ± 0.082	0.692 ± 0.125	0.801 ± 0.128	0.290 ± 0.171	0.849 ± 0.075	0.581 ± 0.305	0.842 ± 0.170
MNC-DHC	0.806 ± 0.063	0.910 ± 0.043	0.854 ± 0.068	0.978 ± 0.015	0.507 ± 0.144	0.845 ± 0.089	0.814 ± 0.095	0.887 ± 0.057
MNC-CNC	0.774 ± 0.066	0.828 ± 0.061	0.763 ± 0.076	0.798 ± 0.165	0.517 ± 0.111	0.825 ± 0.110	0.850 ± 0.076	0.878 ± 0.057
MSP	0.776 ± 0.072	0.877 ± 0.037	0.804 ± 0.061	0.930 ± 0.060	0.512 ± 0.102	0.886 ± 0.051	0.648 ± 0.262	0.882 ± 0.119
WSC	0.735 ± 0.096	0.854 ± 0.072	0.767 ± 0.112	0.896 ± 0.103	0.468 ± 0.168	0.894 ± 0.058	0.522 ± 0.272	0.873 ± 0.136
NCHSDB	0.753 ± 0.077	0.873 ± 0.043	0.807 ± 0.103	0.859 ± 0.105	0.310 ± 0.167	0.858 ± 0.126	0.897 ± 0.097	0.845 ± 0.153

**OOD Dataset**	**MF1**	**Acc**	κ	**W**	**N1**	**N2**	**N3**	**REM**
DCSM	0.801 ± 0.085	0.927 ± 0.043	0.868 ± 0.076	0.984 ± 0.022	0.499 ± 0.154	0.858 ± 0.097	0.782 ± 0.206	0.886 ± 0.151
SEDF-ST	0.767 ± 0.076	0.842 ± 0.054	0.767 ± 0.077	0.813 ± 0.108	0.508 ± 0.161	0.872 ± 0.061	0.744 ± 0.233	0.897 ± 0.085
SEDF-SC	0.726 ± 0.086	0.915 ± 0.042	0.823 ± 0.079	0.981 ± 0.019	0.343 ± 0.140	0.822 ± 0.089	0.606 ± 0.284	0.862 ± 0.100
PHYS	0.690 ± 0.100	0.779 ± 0.094	0.676 ± 0.131	0.742 ± 0.161	0.337 ± 0.156	0.834 ± 0.110	0.711 ± 0.243	0.843 ± 0.172
DOD-H	0.822 ± 0.066	0.895 ± 0.040	0.840 ± 0.067	0.884 ± 0.091	0.531 ± 0.168	0.909 ± 0.045	0.862 ± 0.167	0.923 ± 0.063
DOD-O	0.771 ± 0.094	0.852 ± 0.085	0.772 ± 0.125	0.902 ± 0.094	0.494 ± 0.165	0.864 ± 0.087	0.725 ± 0.280	0.878 ± 0.097
BSWR	0.707 ± 0.114	0.788 ± 0.103	0.687 ± 0.143	0.830 ± 0.141	0.384 ± 0.171	0.807 ± 0.126	0.664 ± 0.279	0.857 ± 0.177
**Avg ID**	0.768 ± 0.047	0.865 ± 0.037	0.795 ± 0.056	0.914 ± 0.055	0.476 ± 0.094	0.861 ± 0.026	0.690 ± 0.152	0.883 ± 0.025
**Avg OOD**	0.755 ± 0.049	0.857 ± 0.059	0.776 ± 0.074	0.877 ± 0.089	0.442 ± 0.084	0.852 ± 0.034	0.728 ± 0.082	0.878 ± 0.027

Supplementary Table 16 U-Sleep$_{\mathbf{EOG}}$ benchmarking. MF1, Accuracy and Cohen's κ, plus Class-Wise F1 score on recording-level for U-Sleep using EOG channel derivations.

**ID Dataset**	**MF1**	**Acc**	κ	**W**	**N1**	**N2**	**N3**	**REM**
ABC	0.766 ± 0.097	0.838 ± 0.058	0.758 ± 0.075	0.884 ± 0.046	0.581 ± 0.079	0.846 ± 0.094	0.612 ± 0.300	0.888 ± 0.200
CCSHS	0.844 ± 0.050	0.910 ± 0.038	0.873 ± 0.052	0.965 ± 0.028	0.571 ± 0.143	0.901 ± 0.051	0.864 ± 0.103	0.919 ± 0.045
CFS	0.791 ± 0.082	0.891 ± 0.058	0.835 ± 0.094	0.959 ± 0.035	0.488 ± 0.158	0.868 ± 0.098	0.753 ± 0.236	0.890 ± 0.112
CHAT	0.824 ± 0.046	0.877 ± 0.048	0.832 ± 0.065	0.951 ± 0.040	0.544 ± 0.115	0.843 ± 0.081	0.891 ± 0.074	0.891 ± 0.067
HOMEPAP	0.726 ± 0.122	0.809 ± 0.100	0.711 ± 0.177	0.873 ± 0.149	0.390 ± 0.160	0.812 ± 0.090	0.681 ± 0.263	0.884 ± 0.171
MESA	0.728 ± 0.094	0.855 ± 0.078	0.781 ± 0.123	0.941 ± 0.070	0.466 ± 0.147	0.833 ± 0.116	0.504 ± 0.299	0.894 ± 0.080
MROS	0.730 ± 0.090	0.885 ± 0.043	0.817 ± 0.069	0.953 ± 0.036	0.411 ± 0.157	0.864 ± 0.063	0.540 ± 0.293	0.870 ± 0.158
SHHS	0.751 ± 0.093	0.860 ± 0.075	0.794 ± 0.112	0.919 ± 0.094	0.410 ± 0.184	0.853 ± 0.083	0.686 ± 0.237	0.894 ± 0.089
SOF	0.733 ± 0.075	0.867 ± 0.061	0.790 ± 0.097	0.941 ± 0.050	0.319 ± 0.169	0.831 ± 0.089	0.670 ± 0.204	0.905 ± 0.089
APOE	0.717 ± 0.101	0.814 ± 0.079	0.713 ± 0.119	0.850 ± 0.086	0.440 ± 0.172	0.853 ± 0.091	0.562 ± 0.317	0.855 ± 0.146
APPLES	0.723 ± 0.093	0.818 ± 0.086	0.729 ± 0.113	0.897 ± 0.058	0.478 ± 0.143	0.834 ± 0.140	0.340 ± 0.317	0.864 ± 0.154
MNC-SSC	0.684 ± 0.107	0.809 ± 0.073	0.701 ± 0.117	0.807 ± 0.123	0.310 ± 0.176	0.857 ± 0.060	0.586 ± 0.314	0.853 ± 0.159
MNC-DHC	0.798 ± 0.066	0.902 ± 0.049	0.842 ± 0.075	0.972 ± 0.024	0.473 ± 0.140	0.843 ± 0.070	0.824 ± 0.076	0.876 ± 0.089
MNC-CNC	0.765 ± 0.074	0.817 ± 0.060	0.747 ± 0.079	0.799 ± 0.142	0.495 ± 0.182	0.822 ± 0.113	0.846 ± 0.071	0.879 ± 0.138
MSP	0.756 ± 0.091	0.858 ± 0.044	0.775 ± 0.075	0.898 ± 0.076	0.439 ± 0.143	0.874 ± 0.052	0.669 ± 0.283	0.875 ± 0.128
WSC	0.710 ± 0.098	0.847 ± 0.072	0.753 ± 0.115	0.870 ± 0.116	0.452 ± 0.150	0.893 ± 0.058	0.423 ± 0.281	0.879 ± 0.138
NCHSDB	0.746 ± 0.080	0.857 ± 0.049	0.785 ± 0.105	0.844 ± 0.115	0.329 ± 0.176	0.841 ± 0.128	0.891 ± 0.085	0.830 ± 0.152
**OOD Dataset**	**MF1**	**Acc**	κ	**W**	**N1**	**N2**	**N3**	**REM**
DCSM	0.778 ± 0.085	0.914 ± 0.048	0.843 ± 0.083	0.980 ± 0.024	0.471 ± 0.143	0.833 ± 0.104	0.733 ± 0.213	0.878 ± 0.136
SEDF-ST	0.712 ± 0.068	0.803 ± 0.049	0.711 ± 0.072	0.748 ± 0.131	0.410 ± 0.123	0.846 ± 0.057	0.688 ± 0.240	0.869 ± 0.078
SEDF-SC	0.641 ± 0.095	0.846 ± 0.073	0.708 ± 0.119	0.938 ± 0.051	0.292 ± 0.127	0.756 ± 0.105	0.529 ± 0.285	0.677 ± 0.179
PHYS	0.658 ± 0.105	0.753 ± 0.093	0.635 ± 0.129	0.737 ± 0.164	0.401 ± 0.149	0.804 ± 0.112	0.526 ± 0.299	0.826 ± 0.178
DOD-H	0.749 ± 0.161	0.824 ± 0.130	0.742 ± 0.180	0.785 ± 0.171	0.513 ± 0.186	0.829 ± 0.180	0.750 ± 0.231	0.868 ± 0.201
DOD-O	0.736 ± 0.087	0.846 ± 0.063	0.760 ± 0.097	0.894 ± 0.068	0.490 ± 0.129	0.868 ± 0.065	0.514 ± 0.340	0.907 ± 0.096
BSWR	0.691 ± 0.118	0.770 ± 0.106	0.661 ± 0.146	0.817 ± 0.148	0.440 ± 0.164	0.785 ± 0.131	0.580 ± 0.307	0.835 ± 0.186
**Avg ID**	0.752 ± 0.042	0.854 ± 0.033	0.779 ± 0.050	0.901 ± 0.055	0.447 ± 0.081	0.851 ± 0.024	0.667 ± 0.165	0.878 ± 0.021
**Avg OOD**	0.709 ± 0.049	0.822 ± 0.054	0.723 ± 0.068	0.843 ± 0.095	0.431 ± 0.074	0.817 ± 0.038	0.617 ± 0.103	0.837 ± 0.076

Supplementary Table 17 DeepResNet$_{\mathbf{EOG}}$ benchmarking. MF1, Accuracy and Cohen's κ, plus Class-Wise F1 score on recording-level for DeepResNet using EOG channel derivations.

**ID Dataset**	**MF1**	**Acc**	κ	**W**	**N1**	**N2**	**N3**	**REM**
ABC	0.762 ± 0.095	0.834 ± 0.059	0.755 ± 0.073	0.879 ± 0.044	0.587 ± 0.081	0.846 ± 0.092	0.595 ± 0.316	0.880 ± 0.199
CCSHS	0.841 ± 0.048	0.909 ± 0.037	0.872 ± 0.051	0.967 ± 0.020	0.562 ± 0.133	0.899 ± 0.051	0.865 ± 0.093	0.914 ± 0.052
CFS	0.786 ± 0.085	0.889 ± 0.064	0.833 ± 0.102	0.956 ± 0.044	0.474 ± 0.172	0.869 ± 0.100	0.732 ± 0.254	0.902 ± 0.089
CHAT	0.813 ± 0.052	0.870 ± 0.050	0.824 ± 0.068	0.943 ± 0.048	0.517 ± 0.125	0.841 ± 0.082	0.893 ± 0.073	0.871 ± 0.078
HOMEPAP	0.720 ± 0.135	0.797 ± 0.129	0.704 ± 0.177	0.881 ± 0.115	0.405 ± 0.155	0.788 ± 0.159	0.656 ± 0.290	0.877 ± 0.178
MESA	0.731 ± 0.092	0.858 ± 0.072	0.785 ± 0.116	0.943 ± 0.064	0.485 ± 0.145	0.837 ± 0.112	0.499 ± 0.300	0.888 ± 0.076
MROS	0.724 ± 0.090	0.882 ± 0.044	0.812 ± 0.069	0.950 ± 0.036	0.396 ± 0.149	0.861 ± 0.060	0.532 ± 0.302	0.869 ± 0.145
SHHS	0.739 ± 0.093	0.853 ± 0.079	0.783 ± 0.118	0.913 ± 0.100	0.397 ± 0.180	0.846 ± 0.090	0.656 ± 0.255	0.892 ± 0.085
SOF	0.729 ± 0.080	0.861 ± 0.067	0.782 ± 0.102	0.938 ± 0.056	0.334 ± 0.143	0.823 ± 0.095	0.645 ± 0.218	0.906 ± 0.077
APOE	0.713 ± 0.106	0.814 ± 0.080	0.713 ± 0.120	0.858 ± 0.079	0.449 ± 0.169	0.852 ± 0.090	0.510 ± 0.334	0.860 ± 0.136
APPLES	0.718 ± 0.090	0.820 ± 0.078	0.729 ± 0.106	0.896 ± 0.059	0.476 ± 0.144	0.833 ± 0.141	0.295 ± 0.297	0.869 ± 0.141
MNC-SSC	0.670 ± 0.103	0.800 ± 0.077	0.684 ± 0.119	0.795 ± 0.128	0.313 ± 0.162	0.851 ± 0.066	0.536 ± 0.304	0.845 ± 0.167
MNC-DHC	0.793 ± 0.070	0.902 ± 0.051	0.839 ± 0.084	0.969 ± 0.033	0.468 ± 0.133	0.838 ± 0.079	0.803 ± 0.126	0.886 ± 0.070
MNC-CNC	0.755 ± 0.071	0.807 ± 0.056	0.735 ± 0.070	0.763 ± 0.168	0.500 ± 0.168	0.811 ± 0.095	0.829 ± 0.068	0.871 ± 0.054
MSP	0.749 ± 0.083	0.857 ± 0.034	0.775 ± 0.057	0.904 ± 0.073	0.459 ± 0.156	0.869 ± 0.060	0.625 ± 0.269	0.863 ± 0.122
WSC	0.696 ± 0.101	0.842 ± 0.078	0.744 ± 0.121	0.866 ± 0.117	0.466 ± 0.153	0.889 ± 0.066	0.347 ± 0.289	0.873 ± 0.142
NCHSDB	0.733 ± 0.084	0.851 ± 0.049	0.777 ± 0.104	0.825 ± 0.129	0.295 ± 0.166	0.839 ± 0.120	0.886 ± 0.097	0.825 ± 0.149
**OOD Dataset**	**MF1**	**Acc**	κ	**W**	**N1**	**N2**	**N3**	**REM**
DCSM	0.777 ± 0.086	0.912 ± 0.048	0.840 ± 0.082	0.979 ± 0.024	0.471 ± 0.143	0.825 ± 0.106	0.721 ± 0.223	0.888 ± 0.123
SEDF-ST	0.698 ± 0.070	0.794 ± 0.056	0.697 ± 0.074	0.737 ± 0.132	0.405 ± 0.131	0.835 ± 0.069	0.643 ± 0.272	0.868 ± 0.075
SEDF-SC	0.676 ± 0.095	0.855 ± 0.076	0.729 ± 0.113	0.935 ± 0.064	0.410 ± 0.133	0.797 ± 0.093	0.534 ± 0.292	0.680 ± 0.172
PHYS	0.660 ± 0.102	0.752 ± 0.090	0.635 ± 0.127	0.731 ± 0.164	0.404 ± 0.146	0.801 ± 0.115	0.533 ± 0.293	0.836 ± 0.178
DOD-H	0.791 ± 0.081	0.874 ± 0.050	0.812 ± 0.080	0.824 ± 0.148	0.496 ± 0.172	0.892 ± 0.052	0.833 ± 0.155	0.914 ± 0.088
DOD-O	0.744 ± 0.080	0.845 ± 0.062	0.760 ± 0.092	0.897 ± 0.062	0.482 ± 0.134	0.864 ± 0.072	0.562 ± 0.300	0.912 ± 0.076
BSWR	0.689 ± 0.115	0.768 ± 0.103	0.657 ± 0.143	0.819 ± 0.143	0.450 ± 0.156	0.783 ± 0.131	0.559 ± 0.306	0.834 ± 0.190
**Avg ID**	0.745 ± 0.043	0.850 ± 0.035	0.773 ± 0.052	0.897 ± 0.061	0.446 ± 0.081	0.847 ± 0.027	0.641 ± 0.179	0.876 ± 0.022
**Avg OOD**	0.719 ± 0.051	0.829 ± 0.059	0.733 ± 0.076	0.846 ± 0.095	0.445 ± 0.039	0.828 ± 0.039	0.626 ± 0.114	0.847 ± 0.081

Supplementary Table 18 SleepTransformer$_{\mathbf{EOG}}$ benchmarking. MF1, Accuracy and Cohen's κ, plus Class-Wise F1 score on recording-level for SleepTransformer using EOG channel derivations.

**Dataset**	**MF1**	**Acc**	κ	**W**	**N1**	**N2**	**N3**	**REM**
ABC	0.772 ± 0.090	0.831 ± 0.053	0.754 ± 0.066	0.882 ± 0.043	0.609 ± 0.080	0.827 ± 0.118	0.646 ± 0.277	0.881 ± 0.199
CCSHS	0.840 ± 0.052	0.905 ± 0.040	0.867 ± 0.055	0.967 ± 0.022	0.572 ± 0.142	0.892 ± 0.055	0.853 ± 0.115	0.913 ± 0.058
CFS	0.787 ± 0.085	0.883 ± 0.066	0.825 ± 0.106	0.956 ± 0.043	0.483 ± 0.166	0.856 ± 0.106	0.747 ± 0.239	0.897 ± 0.093
CHAT	0.840 ± 0.041	0.880 ± 0.043	0.838 ± 0.058	0.957 ± 0.035	0.614 ± 0.096	0.842 ± 0.079	0.891 ± 0.073	0.895 ± 0.054
HOMEPAP	0.758 ± 0.086	0.828 ± 0.072	0.741 ± 0.122	0.904 ± 0.068	0.482 ± 0.156	0.819 ± 0.100	0.695 ± 0.258	0.908 ± 0.076
MESA	0.737 ± 0.095	0.855 ± 0.082	0.784 ± 0.119	0.945 ± 0.074	0.504 ± 0.139	0.829 ± 0.110	0.512 ± 0.296	0.891 ± 0.089
MROS	0.727 ± 0.089	0.880 ± 0.044	0.810 ± 0.070	0.951 ± 0.039	0.426 ± 0.157	0.856 ± 0.062	0.514 ± 0.298	0.874 ± 0.144
SHHS	0.753 ± 0.094	0.859 ± 0.071	0.792 ± 0.113	0.918 ± 0.087	0.412 ± 0.190	0.846 ± 0.092	0.709 ± 0.232	0.888 ± 0.097
SOF	0.735 ± 0.080	0.860 ± 0.073	0.782 ± 0.112	0.937 ± 0.061	0.358 ± 0.164	0.818 ± 0.095	0.661 ± 0.231	0.909 ± 0.069
APOE	0.727 ± 0.099	0.821 ± 0.075	0.727 ± 0.113	0.858 ± 0.081	0.469 ± 0.165	0.856 ± 0.090	0.560 ± 0.336	0.866 ± 0.126
APPLES	0.724 ± 0.101	0.827 ± 0.071	0.739 ± 0.096	0.901 ± 0.057	0.517 ± 0.156	0.833 ± 0.140	0.247 ± 0.305	0.866 ± 0.154
MNC-SSC	0.686 ± 0.108	0.810 ± 0.074	0.704 ± 0.118	0.795 ± 0.132	0.331 ± 0.171	0.860 ± 0.062	0.591 ± 0.313	0.846 ± 0.167
MNC-DHC	0.796 ± 0.050	0.901 ± 0.040	0.840 ± 0.065	0.976 ± 0.014	0.504 ± 0.123	0.815 ± 0.119	0.799 ± 0.096	0.888 ± 0.049
MNC-CNC	0.770 ± 0.066	0.819 ± 0.059	0.753 ± 0.072	0.807 ± 0.145	0.519 ± 0.134	0.814 ± 0.118	0.846 ± 0.064	0.865 ± 0.069
MSP	0.750 ± 0.090	0.848 ± 0.060	0.766 ± 0.089	0.908 ± 0.076	0.465 ± 0.133	0.853 ± 0.063	0.635 ± 0.272	0.861 ± 0.163
WSC	0.720 ± 0.097	0.843 ± 0.072	0.751 ± 0.112	0.876 ± 0.110	0.496 ± 0.139	0.884 ± 0.067	0.433 ± 0.282	0.881 ± 0.137
NCHSDB	0.743 ± 0.085	0.850 ± 0.052	0.780 ± 0.107	0.838 ± 0.137	0.330 ± 0.167	0.835 ± 0.128	0.886 ± 0.072	0.834 ± 0.146
**OOD Dataset**	**MF1**	**Acc**	κ	**W**	**N1**	**N2**	**N3**	**REM**
DCSM	0.785 ± 0.085	0.916 ± 0.051	0.848 ± 0.088	0.979 ± 0.029	0.454 ± 0.147	0.830 ± 0.107	0.787 ± 0.185	0.882 ± 0.147
SEDF-ST	0.711 ± 0.079	0.811 ± 0.051	0.720 ± 0.072	0.749 ± 0.124	0.417 ± 0.150	0.852 ± 0.061	0.646 ± 0.265	0.888 ± 0.071
SEDF-SC	0.670 ± 0.102	0.892 ± 0.057	0.778 ± 0.103	0.975 ± 0.026	0.331 ± 0.147	0.773 ± 0.126	0.404 ± 0.287	0.832 ± 0.130
PHYS	0.663 ± 0.102	0.745 ± 0.090	0.629 ± 0.127	0.733 ± 0.165	0.408 ± 0.148	0.790 ± 0.111	0.563 ± 0.276	0.828 ± 0.179
DOD-H	0.796 ± 0.160	0.862 ± 0.131	0.798 ± 0.177	0.844 ± 0.122	0.567 ± 0.190	0.865 ± 0.184	0.803 ± 0.233	0.899 ± 0.192
DOD-O	0.592 ± 0.113	0.751 ± 0.078	0.608 ± 0.117	0.721 ± 0.132	0.227 ± 0.144	0.812 ± 0.080	0.524 ± 0.323	0.674 ± 0.244
BSWR	0.699 ± 0.119	0.774 ± 0.109	0.670 ± 0.151	0.817 ± 0.148	0.397 ± 0.177	0.784 ± 0.134	0.660 ± 0.279	0.845 ± 0.186
**Avg ID**	0.757 ± 0.041	0.853 ± 0.029	0.780 ± 0.045	0.904 ± 0.055	0.476 ± 0.085	0.843 ± 0.023	0.660 ± 0.175	0.880 ± 0.022
**Avg OOD**	0.702 ± 0.071	0.822 ± 0.069	0.722 ± 0.090	0.831 ± 0.109	0.400 ± 0.105	0.815 ± 0.035	0.627 ± 0.143	0.835 ± 0.077

Supplementary Table 19 SOMNUS$_{\mathbf{EOG}}$ benchmarking. MF1, Accuracy and Cohen's κ, plus Class-Wise F1 score on recording-level for soft unweighted voting ensembling using EOG channel derivations.

**Dataset**	**MF1**	**Acc**	κ	**W**	**N1**	**N2**	**N3**	**REM**
ABC	0.783 ± 0.091	0.847 ± 0.053	0.775 ± 0.066	0.892 ± 0.042	0.611 ± 0.082	0.854 ± 0.096	0.650 ± 0.298	0.890 ± 0.200
CCSHS	0.854 ± 0.050	0.915 ± 0.036	0.881 ± 0.050	0.970 ± 0.019	0.602 ± 0.141	0.906 ± 0.051	0.869 ± 0.100	0.924 ± 0.048
CFS	0.801 ± 0.081	0.896 ± 0.062	0.842 ± 0.099	0.960 ± 0.039	0.506 ± 0.162	0.874 ± 0.099	0.760 ± 0.239	0.908 ± 0.087
CHAT	0.839 ± 0.044	0.885 ± 0.046	0.844 ± 0.062	0.957 ± 0.036	0.591 ± 0.108	0.852 ± 0.081	0.897 ± 0.073	0.898 ± 0.060
HOMEPAP	0.751 ± 0.095	0.826 ± 0.083	0.740 ± 0.134	0.903 ± 0.075	0.433 ± 0.161	0.825 ± 0.087	0.708 ± 0.257	0.899 ± 0.122
MESA	0.741 ± 0.094	0.864 ± 0.075	0.794 ± 0.119	0.947 ± 0.065	0.498 ± 0.149	0.841 ± 0.113	0.515 ± 0.305	0.900 ± 0.077
MROS	0.735 ± 0.088	0.889 ± 0.042	0.823 ± 0.066	0.955 ± 0.036	0.433 ± 0.158	0.868 ± 0.059	0.534 ± 0.302	0.876 ± 0.149
SHHS	0.759 ± 0.091	0.866 ± 0.072	0.803 ± 0.111	0.924 ± 0.088	0.424 ± 0.194	0.858 ± 0.084	0.697 ± 0.236	0.898 ± 0.088
SOF	0.749 ± 0.074	0.874 ± 0.065	0.803 ± 0.099	0.944 ± 0.052	0.363 ± 0.147	0.839 ± 0.090	0.680 ± 0.213	0.925 ± 0.065
APOE	0.734 ± 0.102	0.829 ± 0.077	0.736 ± 0.117	0.872 ± 0.075	0.471 ± 0.174	0.863 ± 0.090	0.567 ± 0.327	0.872 ± 0.131
APPLES	0.731 ± 0.097	0.834 ± 0.074	0.749 ± 0.101	0.905 ± 0.055	0.508 ± 0.151	0.846 ± 0.133	0.289 ± 0.319	0.873 ± 0.147
MNC-SSC	0.691 ± 0.106	0.818 ± 0.071	0.713 ± 0.115	0.804 ± 0.127	0.324 ± 0.174	0.866 ± 0.059	0.597 ± 0.312	0.858 ± 0.166
MNC-DHC	0.811 ± 0.059	0.909 ± 0.043	0.853 ± 0.067	0.974 ± 0.023	0.512 ± 0.149	0.845 ± 0.078	0.819 ± 0.088	0.904 ± 0.054
MNC-CNC	0.780 ± 0.066	0.829 ± 0.055	0.766 ± 0.069	0.801 ± 0.147	0.530 ± 0.144	0.831 ± 0.102	0.857 ± 0.067	0.880 ± 0.057
MSP	0.774 ± 0.092	0.871 ± 0.043	0.795 ± 0.075	0.915 ± 0.060	0.493 ± 0.144	0.880 ± 0.056	0.680 ± 0.276	0.877 ± 0.143
WSC	0.720 ± 0.098	0.854 ± 0.071	0.765 ± 0.112	0.881 ± 0.110	0.488 ± 0.150	0.898 ± 0.057	0.415 ± 0.292	0.885 ± 0.138
NCHSDB	0.754 ± 0.080	0.866 ± 0.042	0.798 ± 0.098	0.850 ± 0.122	0.336 ± 0.170	0.850 ± 0.125	0.896 ± 0.076	0.847 ± 0.142
**OOD Dataset**	**MF1**	**Acc**	κ	**W**	**N1**	**N2**	**N3**	**REM**
DCSM	0.794 ± 0.084	0.921 ± 0.047	0.856 ± 0.082	0.981 ± 0.026	0.487 ± 0.149	0.844 ± 0.105	0.769 ± 0.200	0.892 ± 0.139
SEDF-ST	0.722 ± 0.073	0.817 ± 0.047	0.729 ± 0.067	0.758 ± 0.130	0.436 ± 0.150	0.855 ± 0.055	0.664 ± 0.266	0.894 ± 0.072
SEDF-SC	0.712 ± 0.093	0.901 ± 0.053	0.802 ± 0.092	0.974 ± 0.027	0.379 ± 0.136	0.811 ± 0.100	0.547 ± 0.284	0.827 ± 0.133
PHYS	0.673 ± 0.103	0.764 ± 0.089	0.652 ± 0.124	0.740 ± 0.164	0.412 ± 0.152	0.815 ± 0.108	0.562 ± 0.293	0.846 ± 0.173
DOD-H	0.810 ± 0.087	0.881 ± 0.063	0.824 ± 0.090	0.838 ± 0.140	0.554 ± 0.203	0.894 ± 0.066	0.835 ± 0.157	0.927 ± 0.061
DOD-O	0.744 ± 0.086	0.855 ± 0.059	0.773 ± 0.089	0.896 ± 0.062	0.484 ± 0.141	0.874 ± 0.064	0.548 ± 0.336	0.916 ± 0.080
BSWR	0.709 ± 0.116	0.786 ± 0.103	0.685 ± 0.143	0.830 ± 0.142	0.435 ± 0.170	0.800 ± 0.127	0.629 ± 0.298	0.857 ± 0.180
**Avg ID**	0.765 ± 0.043	0.863 ± 0.030	0.793 ± 0.046	0.909 ± 0.054	0.478 ± 0.085	0.859 ± 0.022	0.672 ± 0.173	0.889 ± 0.021
**Avg OOD**	0.738 ± 0.049	0.846 ± 0.059	0.760 ± 0.075	0.860 ± 0.096	0.455 ± 0.058	0.842 ± 0.035	0.651 ± 0.114	0.880 ± 0.037

Supplementary Table 20 Performance SleepTransformer SHHS. MF1, Accuracy and Cohen's κ, plus Class-Wise F1 score on recording-level for SleepTransformer making use of EEG channel derivations (majority voting) and trained only on the SHHS dataset, as opposed to the entire NSRR repository.

**Dataset**	**MF1**	**Acc**	κ	**W**	**N1**	**N2**	**N3**	**REM**
ABC	0.674 ± 0.083	0.756 ± 0.065	0.643 ± 0.089	0.831 ± 0.077	0.307 ± 0.109	0.765 ± 0.107	0.616 ± 0.236	0.850 ± 0.194
CCSHS	0.716 ± 0.086	0.810 ± 0.085	0.738 ± 0.113	0.902 ± 0.098	0.344 ± 0.151	0.774 ± 0.120	0.746 ± 0.156	0.815 ± 0.098
CFS	0.693 ± 0.116	0.822 ± 0.099	0.737 ± 0.140	0.910 ± 0.097	0.320 ± 0.179	0.775 ± 0.145	0.694 ± 0.225	0.762 ± 0.222
CHAT	0.418 ± 0.120	0.522 ± 0.127	0.352 ± 0.167	0.673 ± 0.186	0.021 ± 0.035	0.315 ± 0.198	0.631 ± 0.127	0.450 ± 0.272
HOMEPAP	0.615 ± 0.088	0.722 ± 0.109	0.599 ± 0.139	0.851 ± 0.097	0.149 ± 0.119	0.676 ± 0.131	0.630 ± 0.233	0.789 ± 0.235
MESA	0.573 ± 0.117	0.739 ± 0.096	0.611 ± 0.150	0.860 ± 0.112	0.145 ± 0.111	0.704 ± 0.157	0.463 ± 0.310	0.694 ± 0.238
MROS	0.637 ± 0.093	0.811 ± 0.069	0.706 ± 0.102	0.931 ± 0.048	0.228 ± 0.137	0.744 ± 0.107	0.509 ± 0.246	0.761 ± 0.199
SHHS	0.710 ± 0.121	0.835 ± 0.100	0.758 ± 0.146	0.906 ± 0.111	0.303 ± 0.175	0.821 ± 0.121	0.721 ± 0.236	0.807 ± 0.198
SOF	0.682 ± 0.086	0.829 ± 0.082	0.736 ± 0.122	0.923 ± 0.064	0.220 ± 0.148	0.769 ± 0.129	0.709 ± 0.188	0.793 ± 0.178
APOE	0.564 ± 0.112	0.625 ± 0.132	0.483 ± 0.162	0.817 ± 0.134	0.200 ± 0.147	0.621 ± 0.170	0.385 ± 0.251	0.767 ± 0.194
APPLES	0.625 ± 0.112	0.721 ± 0.122	0.598 ± 0.151	0.874 ± 0.092	0.239 ± 0.139	0.728 ± 0.164	0.364 ± 0.318	0.791 ± 0.192
MNC-SSC	0.583 ± 0.114	0.695 ± 0.117	0.550 ± 0.159	0.756 ± 0.153	0.178 ± 0.136	0.723 ± 0.133	0.471 ± 0.275	0.775 ± 0.192
MNC-DHC	0.661 ± 0.082	0.776 ± 0.115	0.663 ± 0.157	0.857 ± 0.120	0.275 ± 0.117	0.742 ± 0.117	0.793 ± 0.095	0.638 ± 0.190
MNC-CNC	0.593 ± 0.122	0.678 ± 0.094	0.565 ± 0.121	0.717 ± 0.172	0.175 ± 0.146	0.659 ± 0.161	0.725 ± 0.074	0.690 ± 0.226
MSP	0.588 ± 0.111	0.754 ± 0.068	0.615 ± 0.101	0.791 ± 0.116	0.265 ± 0.148	0.770 ± 0.105	0.386 ± 0.291	0.694 ± 0.251
WSC	0.589 ± 0.104	0.682 ± 0.112	0.537 ± 0.153	0.846 ± 0.127	0.226 ± 0.152	0.706 ± 0.123	0.362 ± 0.242	0.778 ± 0.181
NCHSDB	0.464 ± 0.170	0.590 ± 0.179	0.423 ± 0.238	0.619 ± 0.254	0.071 ± 0.110	0.443 ± 0.287	0.671 ± 0.153	0.523 ± 0.295
**OOD Dataset**	**MF1**	**Acc**	κ	**W**	**N1**	**N2**	**N3**	**REM**
DCSM	0.611 ± 0.110	0.760 ± 0.117	0.617 ± 0.156	0.837 ± 0.128	0.229 ± 0.143	0.690 ± 0.144	0.743 ± 0.195	0.569 ± 0.238
SEDF-ST	0.612 ± 0.100	0.709 ± 0.110	0.588 ± 0.149	0.701 ± 0.148	0.229 ± 0.139	0.745 ± 0.126	0.661 ± 0.237	0.729 ± 0.159
SEDF-SC	0.551 ± 0.126	0.813 ± 0.093	0.633 ± 0.159	0.954 ± 0.049	0.143 ± 0.116	0.535 ± 0.229	0.413 ± 0.269	0.697 ± 0.202
PHYS	0.621 ± 0.118	0.715 ± 0.123	0.596 ± 0.161	0.728 ± 0.166	0.203 ± 0.146	0.747 ± 0.150	0.667 ± 0.236	0.782 ± 0.207
DOD-H	0.432 ± 0.103	0.496 ± 0.116	0.297 ± 0.149	0.775 ± 0.134	0.082 ± 0.097	0.436 ± 0.200	0.431 ± 0.149	0.427 ± 0.289
DOD-O	0.086 ± 0.074	0.166 ± 0.097	-0.043 ± 0.090	0.038 ± 0.124	0.001 ± 0.008	0.135 ± 0.112	0.251 ± 0.141	0.015 ± 0.108
BSWR	0.615 ± 0.154	0.712 ± 0.158	0.591 ± 0.197	0.764 ± 0.192	0.176 ± 0.141	0.715 ± 0.201	0.705 ± 0.240	0.753 ± 0.255
**Avg ID**	0.611 ± 0.081	0.727 ± 0.089	0.607 ± 0.115	0.827 ± 0.090	0.216 ± 0.087	0.690 ± 0.129	0.581 ± 0.150	0.728 ± 0.106
**Avg OOD**	0.504 ± 0.196	0.624 ± 0.225	0.468 ± 0.254	0.685 ± 0.297	0.152 ± 0.084	0.572 ± 0.226	0.553 ± 0.187	0.567 ± 0.273

Supplementary Table 21 Agreement, expressed by Cohen's κ (mean $\pm$ std), of models with human scorers and consensus on DOD-O and DOD-H datasets. The models are ordered by their agreement with respect to the consensus hypnogram. In the last columns averaged cosine similarity (ACS) quantifies the similarity between the hypnodensities implied by the soft-consensus and the predictions of the models.

	Model	Expert 1	Expert 2	Expert 3	Expert 4	Expert 5	Consensus	ACS
DOD-H	SOMNUS	78.8 ± 17.0	81.7 ± 6.1	81.0 ± 10.2	74.7 ± 12.1	82.1 ± 8.7	**88.9 ± 4.7**	**0.947 ± 0.021**
	$\text{SOMNUS}_{\text{SleepTransformer}}$	78.1 ± 17.1	81.2 ± 6.3	81.4 ± 9.7	74.3 ± 11.6	81.9 ± 8.5	88.4 ± 4.7	0.945 ± 0.021
	$\text{SOMNUS}_{\text{EEG/EOG}}$	77.9 ± 16.7	81.0 ± 6.4	80.0 ± 10.6	74.2 ± 12.6	81.5 ± 9.2	87.9 ± 5.6	0.942 ± 0.023
	$\text{SOMNUS}_{\text{DeepResNet}}$	78.0 ± 16.8	80.6 ± 6.8	80.1 ± 10.8	73.8 ± 12.6	81.3 ± 9.7	87.7 ± 5.9	0.943 ± 0.023
	$\text{SOMNUS}_{\text{EEG}}$	78.3 ± 17.0	80.7 ± 6.4	80.6 ± 10.7	72.9 ± 11.8	80.8 ± 9.6	87.4 ± 5.8	0.938 ± 0.023
	Single Model (Best)	76.3 ± 16.5	79.6 ± 6.2	80.6 ± 9.0	72.4 ± 11.2	80.2 ± 7.6	86.9 ± 4.7	0.939 ± 0.026
	$\text{SOMNUS}_{\text{U-Sleep}}$	77.1 ± 16.5	80.2 ± 7.1	78.5 ± 11.3	73.1 ± 13.2	80.0 ± 10.1	86.0 ± 6.7	0.936 ± 0.028
	$\text{SOMNUS}_{\text{EOG}}$	76.0 ± 17.3	78.8 ± 8.7	78.2 ± 11.4	73.0 ± 13.4	79.3 ± 10.7	85.5 ± 8.5	0.931 ± 0.047
	Single Model (Avg)	74.5 ± 17.9	77.5 ± 9.7	76.8 ± 12.8	70.7 ± 14.3	77.6 ± 11.8	83.4 ± 9.4	0.924 ± 0.036
	Single Model (Worst)	69.1 ± 21.9	72.5 ± 17.3	69.7 ± 18.9	66.7 ± 21.6	71.9 ± 18.7	77.1 ± 18.3	0.899 ± 0.059
DOD-O	SOMNUS	74.9 ± 13.6	77.5 ± 10.3	73.0 ± 12.7	78.9 ± 9.7	79.6 ± 9.6	**85.4 ± 8.0**	0.939 ± 0.024
	$\text{SOMNUS}_{\text{U-Sleep}}$	73.7 ± 14.1	76.1 ± 10.4	73.4 ± 12.8	78.9 ± 9.8	79.6 ± 9.3	84.7 ± 7.7	**0.941 ± 0.026**
	$\text{SOMNUS}_{\text{EEG/EOG}}$	73.7 ± 14.1	77.4 ± 11.0	72.4 ± 12.7	79.2 ± 9.0	79.3 ± 9.6	84.6 ± 8.2	0.935 ± 0.026
	$\text{SOMNUS}_{\text{DeepResNet}}$	74.1 ± 13.9	76.1 ± 10.6	72.9 ± 12.8	78.5 ± 9.4	79.0 ± 9.5	84.6 ± 7.9	0.939 ± 0.024
	Single Model (Best)	73.4 ± 14.1	75.9 ± 10.8	72.7 ± 12.6	78.6 ± 8.9	78.7 ± 9.3	84.2 ± 7.6	0.939 ± 0.023
	$\text{SOMNUS}_{\text{EOG}}$	71.6 ± 14.0	72.6 ± 11.7	72.1 ± 13.3	77.5 ± 9.0	77.4 ± 9.0	81.7 ± 8.2	0.922 ± 0.028
	$\text{SOMNUS}_{\text{EEG}}$	73.3 ± 13.3	77.3 ± 9.9	69.4 ± 14.0	73.5 ± 13.4	75.2 ± 12.3	81.1 ± 12.0	0.915 ± 0.044
	Single Model (Avg)	66.7 ± 14.2	69.2 ± 12.2	65.1 ± 13.8	70.2 ± 11.4	70.8 ± 11.7	74.9 ± 10.9	0.892 ± 0.048
	$\text{SOMNUS}_{\text{SleepTransformer}}$	66.7 ± 13.3	70.3 ± 11.9	63.4 ± 13.0	69.5 ± 11.1	70.0 ± 12.1	74.7 ± 11.8	0.881 ± 0.052
	Single Model (Worst)	47.9 ± 14.6	52.8 ± 15.1	41.3 ± 17.2	44.7 ± 17.3	45.6 ± 18.2	49.3 ± 17.5	0.768 ± 0.091

Supplementary Table 22 Per-sleep-stage comparison of SOMNUS versus individual models in SLEEPYLAND. Percentage of cases where SOMNUS achieves higher recording-wise F1-scores (% Better) on a dataset with respect to individual models, the percentage of cases where this improvement is statistically significant (% Statistically Better), and the percentage of cases where SOMNUS performs significantly worse (% Statistically Worse). Significance is based on Wilcoxon one-sided paired tests adjusted for multiple comparisons.

**Sleep Stage**	**% Better**	**% Statistically Better**	**% Statistically Worse**
Wake	88.0	60.6	1.4
N1	79.6	45.4	4.2
N2	95.8	69.0	0.0
N3	84.3	38.4	2.8
REM	96.3	59.7	0.0

List of Supplementary Figures

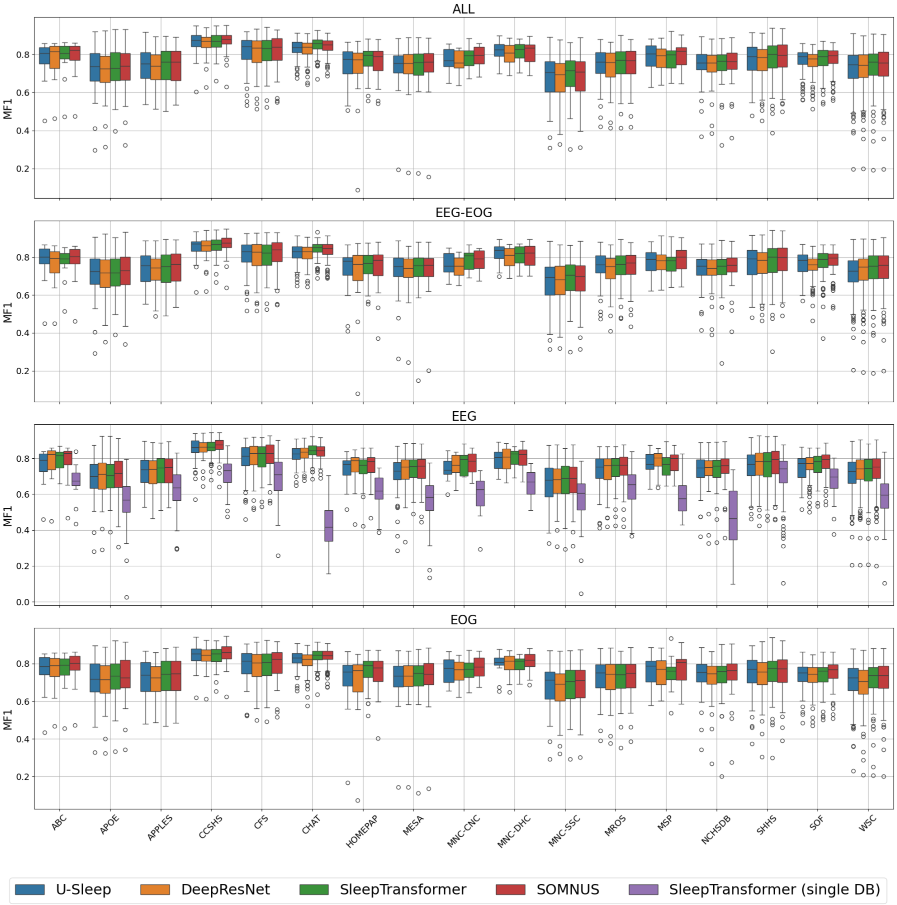

Supplementary Figure 1 Recording-wise Macro-F1 distributions for all models in SLEEPYLAND on all the test set partitions of in-domain (ID) datasets.

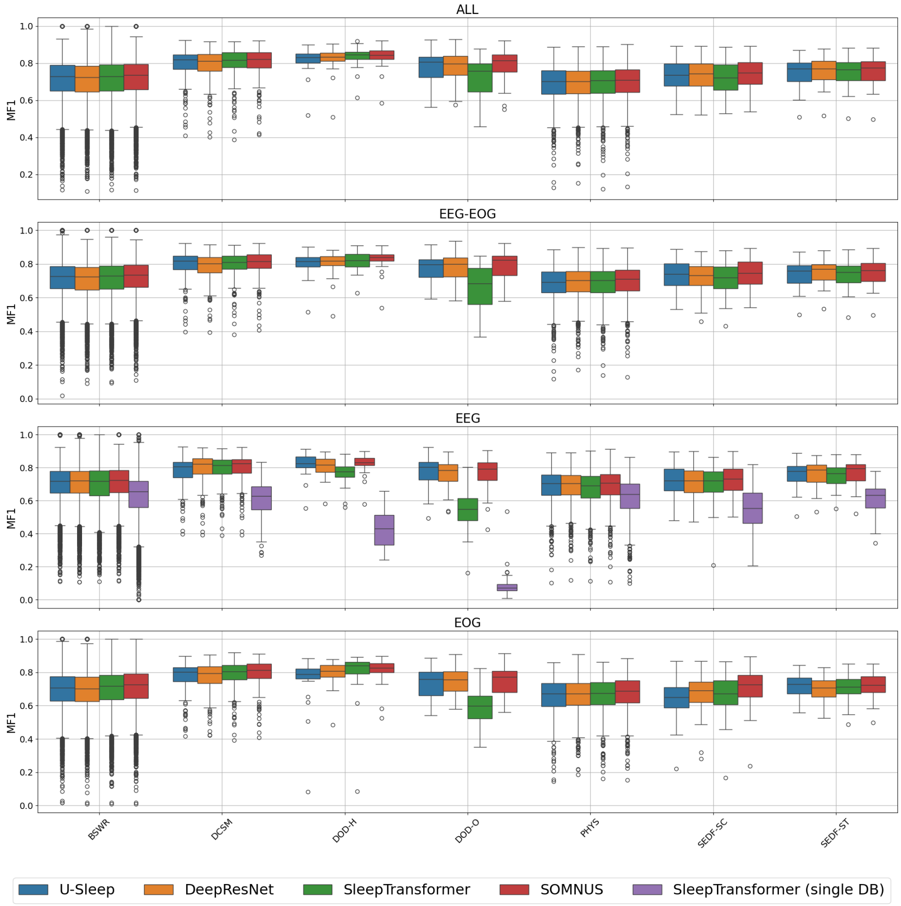

Supplementary Figure 2 Recording-wise Macro-F1 distributions for all models in SLEEPYLAND on out-of-domain (OOD) datasets.

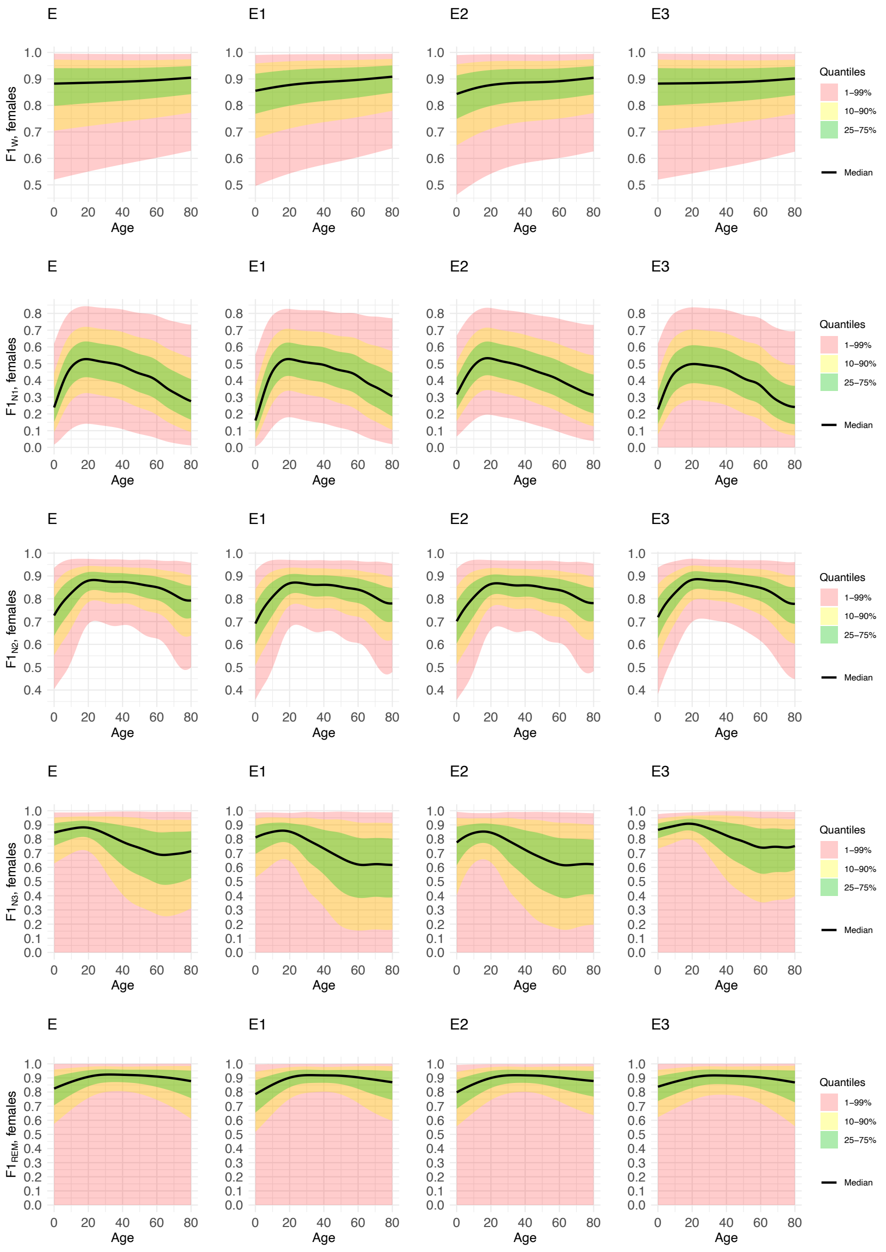

Supplementary Figure 3 Age-conditioned expected distributions of stage-specific F1-scores quantiles for females (under an optimistic scenario of AHI = PLMI = 0) across four sleep scoring models, i.e., SOMNUS (Model E), SOMNUS$_{\text{U-Sleep}}$ (Model E1), SOMNUS$_{\text{DeepResNet}}$ (Model E2), and SOMNUS$_{\text{SleepTransformer}}$ (Model E3).

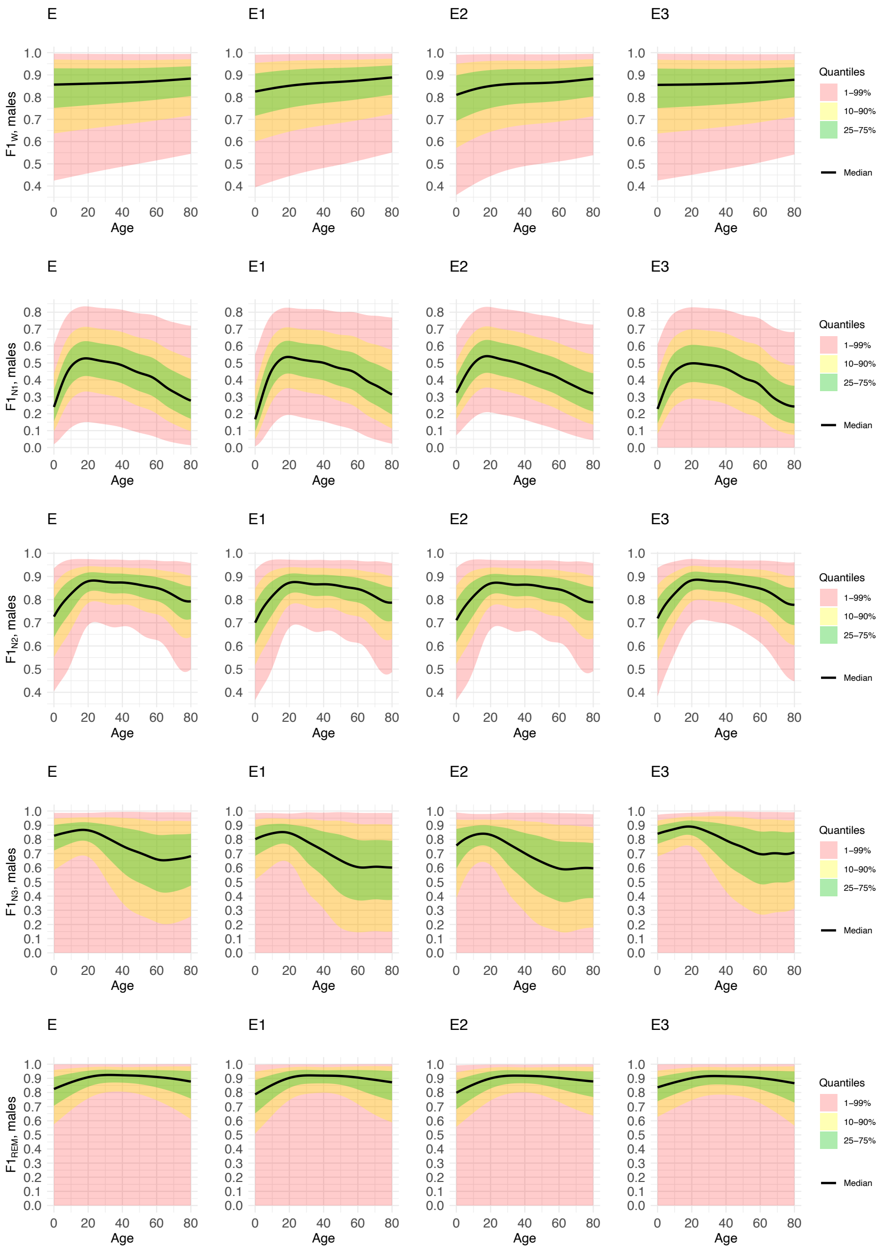

Supplementary Figure 4 Age-conditioned expected distributions of stage-specific F1-scores quantiles for males (under an optimistic scenario of AHI = PLMI = 0) across four sleep scoring models, i.e., SOMNUS (Model E), SOMNUS$_{\text{U-Sleep}}$ (Model E1), SOMNUS$_{\text{DeepResNet}}$ (Model E2), and SOMNUS$_{\text{SleepTransformer}}$ (Model E3).

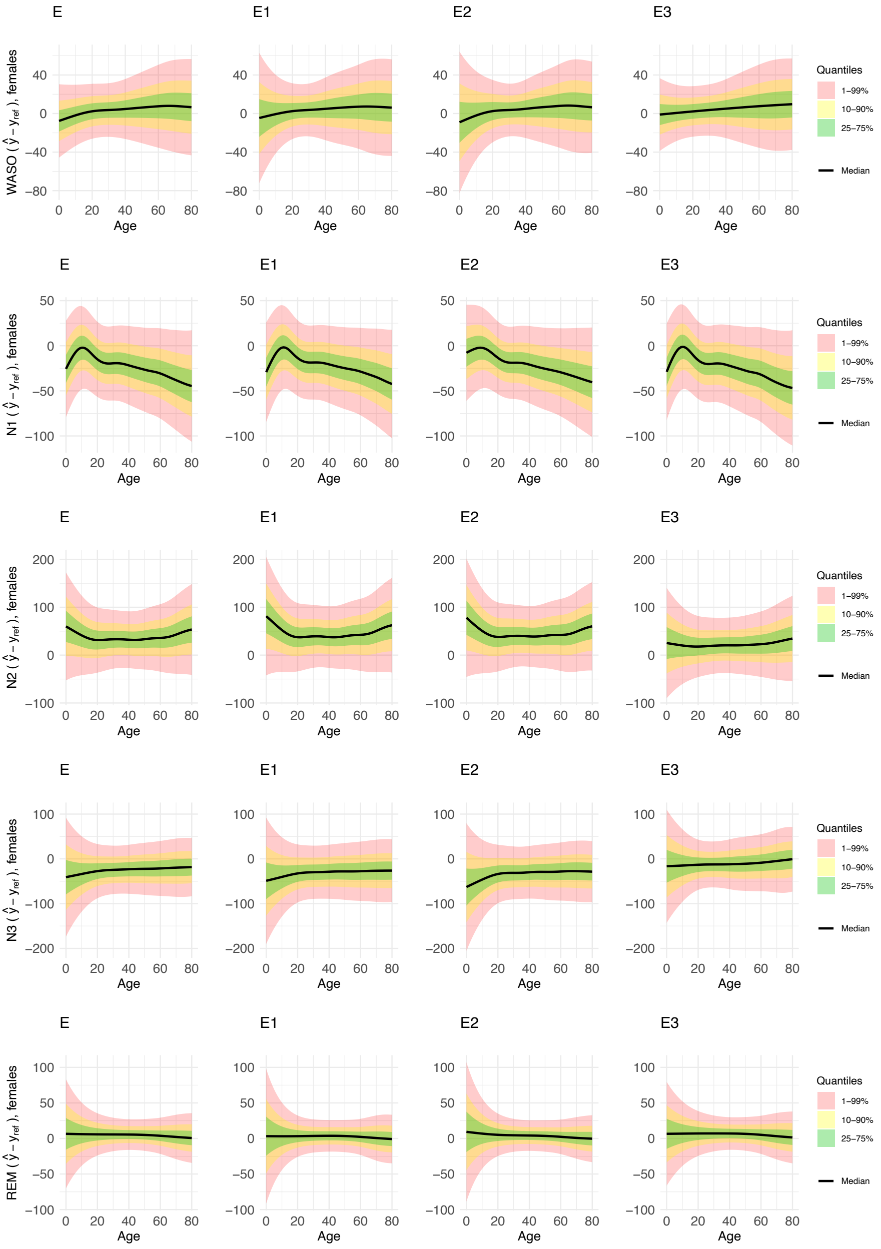

Supplementary Figure 5 Age-conditioned expected distributions of stage-durations quantiles for females (under an optimistic scenario of AHI = PLMI = 0) across four sleep scoring models, i.e., SOMNUS (Model E), $\text{SOMNUS}_{\text{U-Sleep}}$ (Model E1), $\text{SOMNUS}_{\text{DeepResNet}}$ (Model E2), and $\text{SOMNUS}_{\text{SleepTransformer}}$ (Model E3).

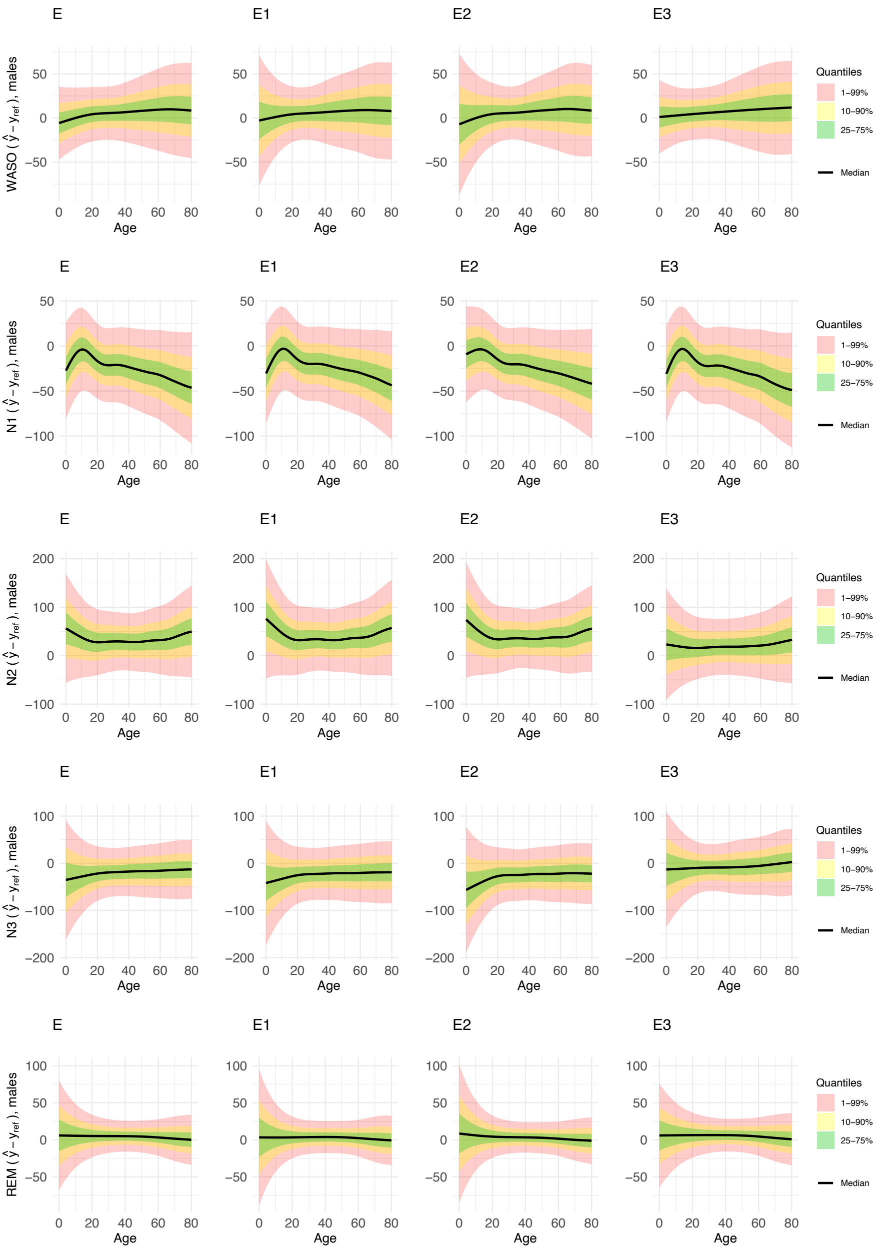

Supplementary Figure 6 Age-conditioned expected distributions of stage-durations quantiles for males (under an optimistic scenario of AHI = PLMI = 0) across four sleep scoring models, i.e., SOMNUS (Model E), SOMNUS$_{\text{U-Sleep}}$ (Model E1), SOMNUS$_{\text{DeepResNet}}$ (Model E2), and SOMNUS$_{\text{SleepTransformer}}$ (Model E3).

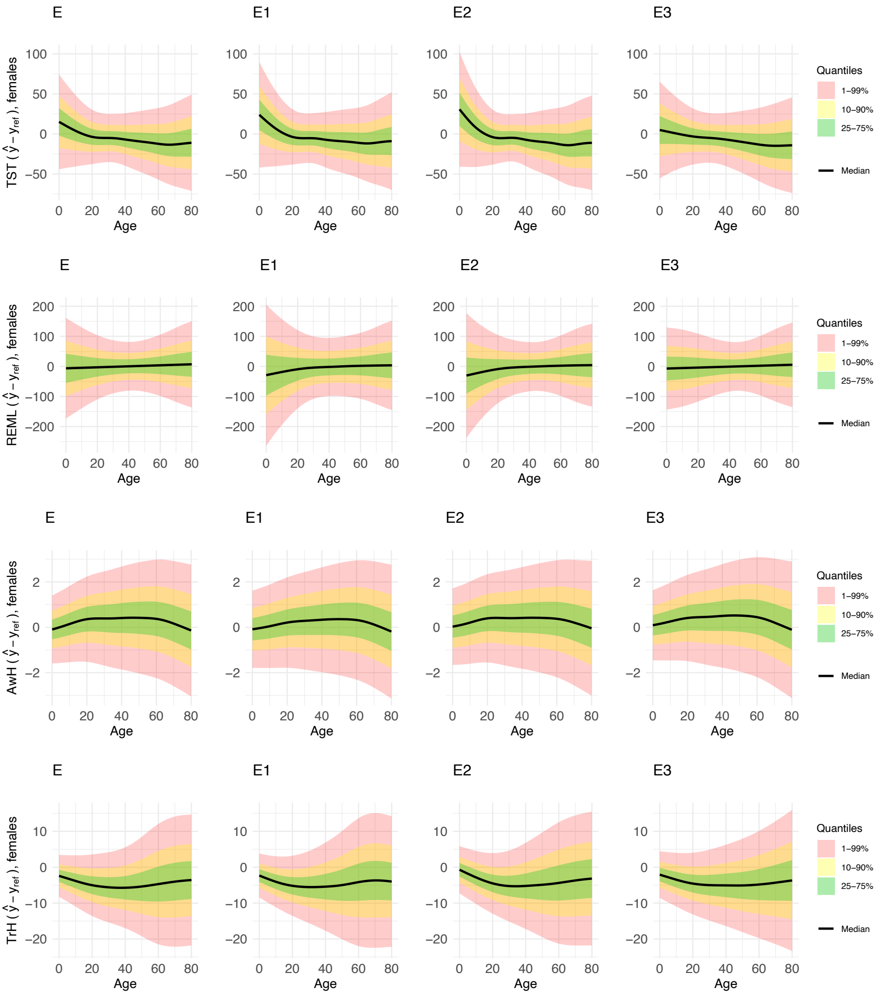

Supplementary Figure 7 Age-conditioned expected distributions of Total Sleep Time (TST), REM latency (REML), Awakenings per Hour (AwH), and Tranistions per Hour (TrH) quantiles for females (under an optimistic scenario of AHI = PLMI = 0) across four sleep scoring models, i.e., SOMNUS (Model E), $\mathrm{SOMNUS}_{\text{U-Sleep}}$ (Model E1), $\mathrm{SOMNUS}_{\text{DeepResNet}}$ (Model E2), and $\mathrm{SOMNUS}_{\text{SleepTransformer}}$ (Model E3).

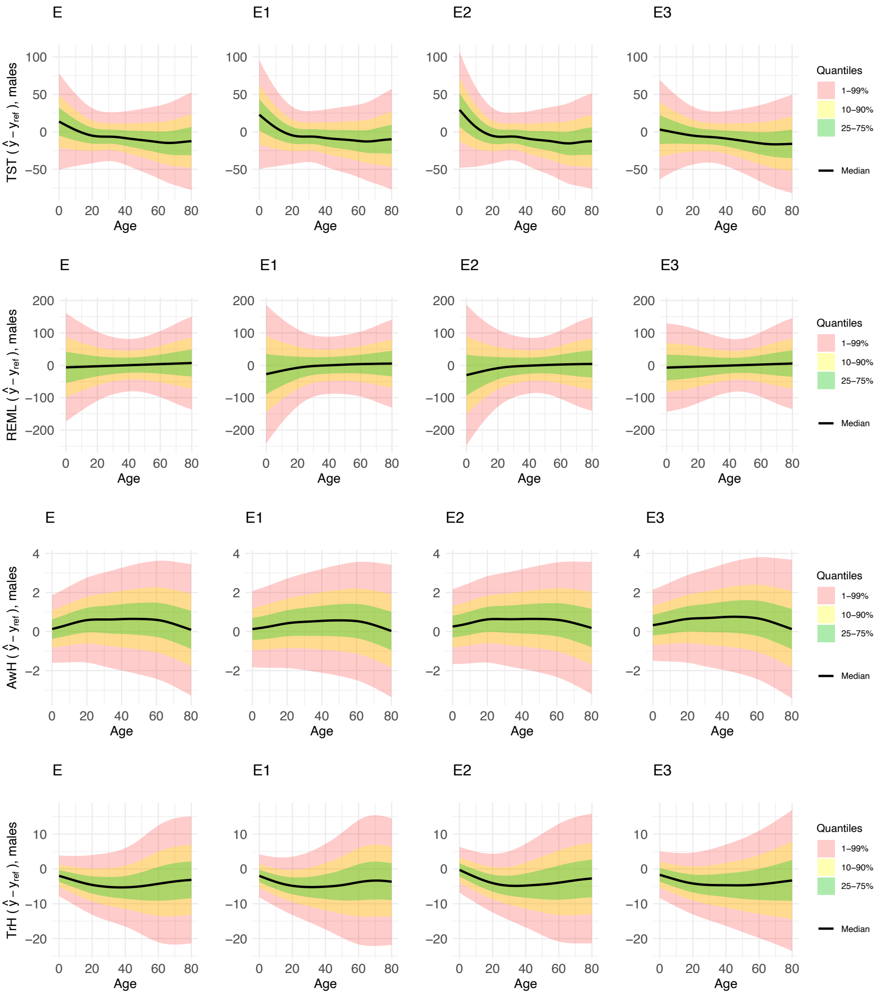

Supplementary Figure 8 Age-conditioned expected distributions of Total Sleep Time (TST), REM latency (REML), Awakenings per Hour (AwH), and Tranistions per Hour (TrH) quantiles for males (under an optimistic scenario of AHI = PLMI = 0) across four sleep scoring models, i.e., SOMNUS (Model E), SOMNUS$_{\text{U-Sleep}}$ (Model E1), SOMNUS$_{\text{DeepResNet}}$ (Model E2), and SOMNUS$_{\text{SleepTransformer}}$ (Model E3).

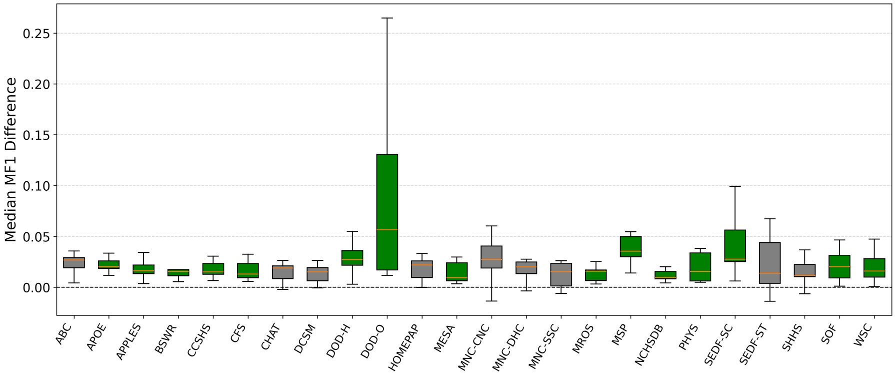

Supplementary Figure 9 Distribution of the differences in median macro-F1 performance between SOMNUS and individual models (specific architecture and channel configuration) for each dataset. Each boxplot represents the variability in median performance differences across all model comparisons for a given dataset. Green boxes indicate datasets where SOMNUS consistently outperformed all individual models, while gray boxes indicate datasets with mixed outcomes. The dashed horizontal line at zero serves as a reference for equal performance.

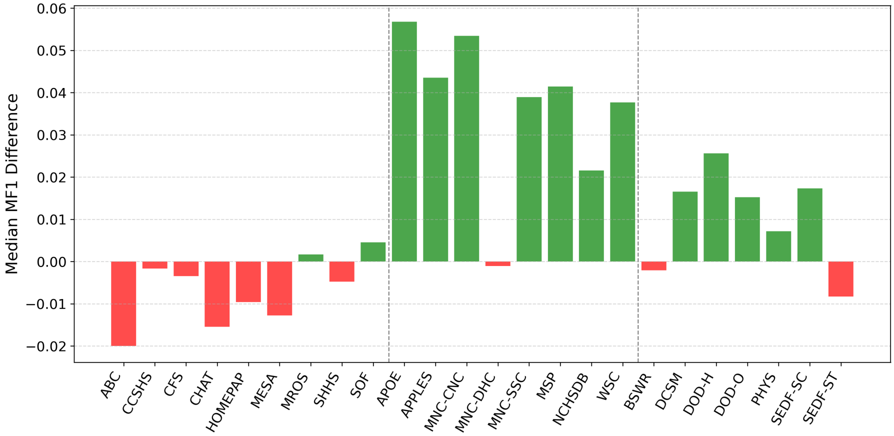

Supplementary Figure 10 Difference in median macro-F1 performance between two SOMNUS configurations: one trained on all 17 NSRR datasets, and another trained on a reduced subset excluding APOE, APPLES, MNC, MSP, NCHSDB, and WSC. Green bars indicate higher performance for the more comprehensively trained model, while red bars indicate decreased performance. Vertical dashed lines delineate: (from left to right) datasets that are in-domain for both models, datasets in-domain only for the version trained on the full dataset collection, and datasets that are out-of-domain for both models.
